# Prognostic biomarkers of malignant patients with pleural effusion: a systematic review and meta-analysis

**DOI:** 10.1186/s12935-022-02518-w

**Published:** 2022-02-24

**Authors:** Peng Peng, Yuan Yang, Juan Du, Kan Zhai, Huan-Zhong Shi

**Affiliations:** 1 Department of Respiratory and Critical Care Medicine, Wuhan Pulmonary Hospital, Wuhan, 430030 China; 2 Department of Respiratory and Critical Care Medicine, Beijing Institute of Respiratory Medicine and Beijing Chao-Yang Hospital, Capital Medical University, 8 Gongti Nanlu, Chao-Yang District, Beijing, 100020 China

**Keywords:** Pleural effusion, Cancer, Prognosis, Systematic review, Meta-analysis

## Abstract

**Background:**

Pleural effusion is a common clinical problem in patients with cancer. We aimed to summarize all the known prognostic indicators of malignant pleural effusion.

**Methods:**

We did a systematic review and meta-analysis with a systematic literature search. All prospective or retrospective cohort studies that estimated the prognostic factors of malignant pleural effusion were enrolled. Mantel–Haenszel method was used to calculate the pooled hazard ratio (HR) and 95% confidence interval (CI).

**Results:**

Eventually, we identified 82 studies with a total of 10,748 patients that met our inclusion criteria. The LENT score showed a good prognostic value (HR 1.97, 95% CI 1.67–2.31) so did the LENT score item. In addition, clinical parameters like stage (HR 1.68, 95% CI 1.25–2.25), distant metastasis (HR 1.62, 95% CI 1.38–1.89), EGFR mutation (HR 0.65, 95% CI 0.56–0.74), serum biological parameters like hemoglobin (HR 1.56, 95% CI 1.17–2.06), albumin (HR 1.71, 95% CI 1.25–2.34), C-reaction protein (HR 1.84, 95% CI 1.49–2.29), VEGF (HR 1.70, 95% CI 1.18–2.43) and pleural effusion biological parameters like PH (HR 1.95, 95% CI 1.46–2.60), glucose (HR 1.75, 95% CI 1.18–2.61), VEGF (HR 1.99, 95% CI 1.67–2.37), and survivin (HR 2.90, 95% CI 1.17–7.20) are also prognostic factors for malignant pleural effusion.

**Conclusions:**

For malignant pleural effusion, LENT score and its items are valuable prognostic biomarkers, so do the clinical parameters like stage, distant metastasis, EGFR mutation, the serum biological parameters like hemoglobin, albumin, C-reaction protein, VEGF and the pleural effusion biological parameters like PH, glucose, VEGF and survivin.

**Supplementary Information:**

The online version contains supplementary material available at 10.1186/s12935-022-02518-w.

## Introduction

Pleural effusion is a common problem in many diseases especially in cancer. It occurs as a result of in situ pleural involvement and/or metastatic malignancy in the pleural cavity resulting in increased vascular permeability, production of excess fluid in excess of lymphatic reabsorption capacity, and/or disruption of lymphatic reabsorption capacity causing fluid accumulation in the pleural cavity [1]. It accounts for greater than 125,000 hospital admissions per year in the United States and estimated inpatient costs of greater than $5 billion per year [2]. The occurrence of pleural effusion in patients with malignancy always indicates disseminated or advanced disease [3]. In lung cancer, it upstages the severity of illness to stage IV [4] and significantly reduces life expectancy in non-small cell lung cancer [5]. The average survival of malignant pleural effusions (MPE) ranges from 4 to 7 months and is dependent on the stage and type of the underlying malignancy [6]. Increasing importance is placed on slowing down disease progression by improving risk factors. However, the factors that define malignant progression and mortality in MPE are poorly understood.

The LENT scoring system is the first validated prognostic score in MPE. It predicts patients’ survival on the basis of tumor type, pleural fluid lactate dehydrogenase (LDH), Eastern Cooperative Oncology Group Performance Status (ECOG PS), and blood neutrophil-to-lymphocyte ratio (NLR) and predicts survival with significantly better accuracy than ECOG PS alone [7]. Another prognostic model for MPE is the PROMISE score which combines biological and clinical parameters to accurately estimate 3-month mortality [8]. The modified LENT score was based on LENT score replacing the "tumor type" score of 2 with 0 in patients with lung adenocarcinoma to illustrated that the actual survival in patients having MPE from lung adenocarcinoma was higher than predicted by the LENT score [9]. A new prognostic model—SELECT prognostication model was proposed recently with high accuracy at identifying patients with high probability of survival at 90 days an Asian population [10]. Notably, EGFR mutations were included in prediction model for the first time. These findings are consistent with our meta-analysis that EGFR is a protective factor for lung cancer. In addition, minimal pleural effusion itself is also an important prognostic factor of worse survival, especially in early-stage malignant disease [11]. Our group has systematically studied the prognostic role of pleural effusion in malignancy and found that whether malignant effusion is clearly diagnosed with cytological or histological examination, pleural effusion is a prognostic factor associated with a poor prognosis for cancer patients. Thus, capturing clinical parameter biomarkers, plasma biomarkers and pleural effusion biomarkers are increasingly important.

We aim to systematically synthesize the published evidence on the associations between the prognostic biomarkers and clinical outcomes in patients with malignant pleural effusion to provide a new insight for development of scoring systems. To our knowledge, no published study has thoroughly and systematically summarized these evidences.

## Methods

### Search strategy and selection criteria

**Fig. 1:**
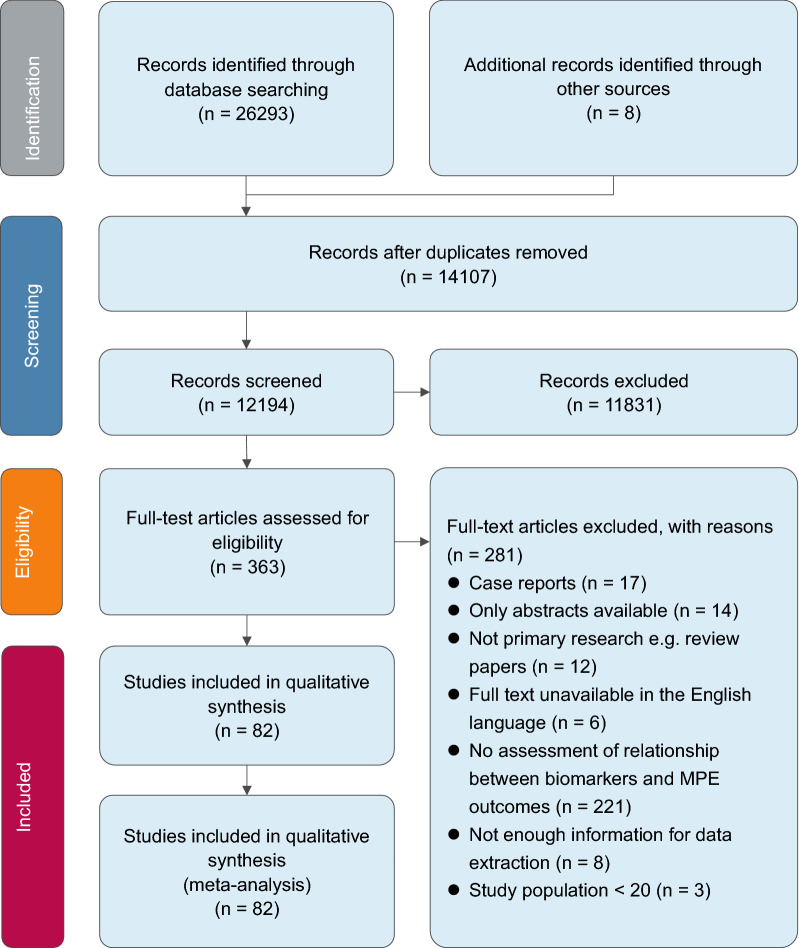
The PRISMA diagram for study selection. PRISMA: Preferred Reporting Items for Systematic Reviews and Meta-Analyses

We conducted a systematic review and meta-analysis to assess the associations between the prognostic factors and clinical outcomes in malignant patients with pleural effusion. The search flow diagram for this meta-analysis is shown in Fig. 1. Databases searched included PubMed, Cochrane Library, Medline (accessed via OVID), Embase, and Web of Science, covering all dates from the creation of each database up to April 2, 2020. The index terms included “pleural effusion”, “malignant”, and “prognosis”, as well as the related words. Additional file 1: eTable 1 presents the detailed search strategy. Additional studies were identified by searching the list of references of included studies, as well as previous relevant meta-analysis and systematic reviews. This meta-analysis was carried out following Preferred Reporting Items for Systematic Reviews and Meta-Analysis (PRISMA) guidelines [12].

### Eligibility criteria

We enrolled prospective or retrospective cohort studies in English that estimated the association between prognostic factors and clinical outcomes in malignant patients with pleural effusion. There were no restrictions on studies with respect to settings, tumor types, or comorbidity types. Inclusion criteria were as follows: (1) study population: patients diagnosed with any type of malignancy and pleural effusion; (2) target: assessing a relevant biomarker; (3) outcomes: overall survival (OS), progression-free survival (PFS); (4) study type: prospective or retrospective cohort studies. The exclusion criteria included (1) studies involving infants, children and adolescents (2) no clearly reported diagnostic criteria for malignant tumor or pleural effusion; (3) narrative reviews, comments, editorials, case reports, meeting abstracts, guidelines or corresponding letters; and (4) full-text paper unavailable in English; (5) sample size < 20. The methods were defined in advance in the original study protocol (Additional file 1, pp 1–2).

### Data extraction and quality assessment

Two authors (YY and DJ) independently screened the title and abstract of the literature retrieved from the databases by the search strategy. Then YY and DJ independently reviewed the full text of articles and assessed articles for eligibility according to the inclusion criteria. Disagreements between the two authors were settled by of arbitration of the principal investigator. Two authors extracted data from included studies using a standardized form based on the Cochrane Consumers and Communication Review Group’s data extraction template. The data extracted by the two authors was cross-checked and differences were resolved by checking the original article. Where data were not enough to extract, the corresponding authors were contacted and asked to provide data.

Data extracted included: (1) clinical characteristics (including age, gender, country, publication year, sample size, and primary tumor type); (2) all kinds of prognostic factors; (3) clinical outcomes (OS and PFS). Cox proportional hazards modeling results of hazard ratio (HR) and 95% confidence interval (CI) of prognostic factors were extracted and we applied the software Origin (version 2020; https://www.originlab.com/) to digitize and extract key data from the published Kaplan–Meier curves. When HR, 95% CI and Kaplan–Meier curves were not given directly, the data extract method was based on the method of Parmar et al. [13].

The quality of each study was assessed in accordance with the Newcastle–Ottawa Scale (NOS) [14]. As all included studies are cohort studies, the scoring was based on the following items: (1) selection: representativeness of exposed cohort, selection of non-exposed cohort, ascertainment of exposure, demonstration that outcome of interest was not present at start of study; (2) comparability: comparability of cohorts on the basis of the design or analysis; (3) outcome: assessment of outcome, was follow-up long enough for outcomes to occur and adequacy of follow up of cohorts. Two reviews (YY and DJ) independently assessed the risk of bias of each trial. They cross checked the data and settled discrepancies by discussion.

### Statistical analysis

The pooled HR and 95% CIs were calculated using Mantel–Haenszel method. A random-effects model was used when significant heterogeneity was observed (I^2^ > 50%); otherwise we used a fixed-effects mode. We further performed sensitivity analysis by omitting one study at a time and examining the influence of each study on the pooled estimates of the primary outcome. In addition, we generated contour-enhanced meta-analysis funnel plots to assess potential publication bias or other biases associated with trial size. A two-sided P value < 0**·**05 was considered statistically significant. The data analyses were performed using software Stata (version 15; https://www.stata.com/).

## Results

### Study selection and characteristics

The systematic review yielded 14,107 references from five electronic databases. Eventually, we identified 82 studies with a total of 10,748 patients that met our inclusion criteria. The sample size ranged from 23 to 789 subjects. The following period ranged from 1 to 264 months. The numbers of studies comparing the OS and PFS difference between different demographic data in malignant patients with pleural effusion were 78 and 9, respectively. All 83 studies included in this systematic review were cohort studies. Figure 1 presents the PRISMA diagram of study selection. All included studies were listed in Additional file 1 (pp 3–8) and their basic characteristics were listed in Table 1.

**Table 1:** Baseline Characteristics of the Included Studies

Study	Year	Country	Age	Sex	Sample size	Follow-up (months)	Cancer type	Outcome
Sahn et al	1988	USA	60.0 ± 1.9	Mixed (53.33% male)	60	37	Mixed (not specified)	OS
Panadero et al	1989	Spain	NA	NA	50	28	Pleural metastatic carcinoma	OS
Foresti et al	1990	Italy	64.8 ± 17.7	Mixed (44.44% male)	36	27	Mixed (lung, breast, mesothelioma, and others)	OS
Gottehrer et al	1991	USA	NA	NA	26	7	MPM	OS
Sugiura et al	1997	Japan	NA	Mixed (67.51% male)	62	54	NSCLC	OS
Moragón et al	1998	Spain	60 ± 13	Mixed (45% male)	120	median 9	Mixed (NSCLC, breast, lymphoma, and others)	OS
Burrows et al	2000	USA	62(24–84)	Mixed (50% male)	85	53	Mixed (lung, breast, mesothelioma, and others)	OS
Heffner et al	2000	USA	61 ± 13	Mixed (50% male)	417	36	Mixed (lung, breast, unknown primary, mesothelioma, and others)	OS
Chen et al	2001	China	67.9 ± 11.2	Mixed (71.29% male)	202	49	Lung cancer	OS
Thyle´n et al	2001	Sweden	NA	Mixed (97% male)	100	100	MPM	OS
Bernard et al	2002	France	65 ± 11	Mixed (45.71% male)	70	> 3	Mixed (breast, unknown, lung, and others)	OS
Eitan et al	2005	USA	55 (26–88)	NA	97	150	Optimally debulked ovarian carcinoma	PFS
Aelony et al	2006	USA	NA	Mixed (92.31% male)	26	18	MPM	OS
Aoe et al	2006	Japan	69(22–95)	Mixed (72.55% male)	102	22	Lung cancer	OS
Soh et al	2006	Japan	NA	Mixed (65.6% male)	61	30	Lung cancer	OS
Bielsa et al	2008	Spain	67 ± 13	Mixed (52.82% male)	284	40	Mixed (lung, breast, unknown, and others)	OS
Wu et al	2008	China	63.4 (37.5–85.4)	Mixed (38.97% male)	136	27	Lung adenocarcinoma	OS
Hsu et al	2009	China	63(27–80)	Mixed (51.5% male)	97	49	NSCLC	OS
Wu et al	2009	China	NA	Mixed (58.33% male)	60	36	Lung cancer	OS
Kotyza et al	2010	Czech Republic	63 ± 11	Mixed (70.73% male)	164	36	Lung cancer	OS
Lan et al	2010	China	59 ± 16	Mixed (55% male)	44	36	Mixed (lung, breast, hepatoma and others)	OS
Ozyurtkan et al	2010	Turkey	59 ± 14	Mixed (56% male)	85	52	Mixed (mesothelioma, lung, ovary, breast, and others)	OS
Pilling et al	2010	UK	60 (26–89)	Mixed (38.85% male)	278	71	Mixed (breast, mesothelioma, lung, ovarian, and others)	OS
Tanrikulu et al	2010	Turkey	NA	Mixed (59.8% male)	363	54	MPM	OS
Hirayama et al	2010	Japan	69.17 ± 9.64	Mixed (82.6% male)	54	20	MPM	OS
Park et al	2011	South Korea	68.3 ± 15.0	Mixed (65.67% male)	67	36	Lung cancer	OS
Sakr et al	2011	France	Median 61	Mixed (46.7% male)	107	120	Mixed (lung, melanoma, breast, ovarian and others)	OS
Yamada et al	2011	Japan	66.16 ± 10.05	Mixed (68.9% male)	45	73	MPM	OS
Guo et al	2011	China	64.5 ± 9.8	Mixed (53.13% male)	128	28	Lung adenocarcinoma	OS
Hooper et al	2012	UK	73(39–96)	Mixed (62.14% male)	103	6	Mixed (mesothelioma, lung, breast, ovarian, and others)	OS
Maribel et al	2012	spain	63(53.2–80.0)	Mixed (66.7% male)	30	40	Lung adenocarcinoma	OS
Qian et al	2012	China	NA	Mixed (60.76% male)	79	18	Lung adenocarcinoma	OS
Qian et al	2012	China	NA	Mixed (61% male)	103	7	Lung adenocarcinoma	PFS
Wang et al	2012	China	NA	Mixed (41.85% male)	184	29	NSCLC	OS
Cheng et al	2013	China	NA	Mixed (63.38% male)	71	60	Lung cancer	OS
Faiz et al	2013	USA	65(17–85)	Mixed (54.95% male)	111	172	Acute leukemia	OS
Gorgun et al	2013	Turkey	60.20 ± 13.91	Mixed (57% male)	51	27	Mixed (lung, breast, pancreas, and others)	OS
Park et al	2013	Korea	68.3 ± 15.0	Mixed (66.25% male)	80	35	Lung cancer	OS
Wu et al	2013	China	27.9–95.5	Mixed (45.5% male)	448	81	Lung adenocarcinoma	OS
Anevlavis et al	2014	Greece	69 (37–93)	Mixed (53% male)	90	56	Mixed (breast, mesothelioma, gastrointestinal, and others)	OS
Clive et al	2014	UK	53–80	Mixed (53.6% male)	789	33	Mixed (mesothelioma, hematological malignancy, gynecological malignancy, breast, and others)	OS
Ni et al	2014	China	31–81	Mixed (47% male)	75	45	NSCLC	OS
Xu et al	2014	China	56.3 ± 12.5	Mixed (46.15% male)	78	16	Lung cancer	OS
Zhang et al	2014	China	64(36–84)	Mixed (51% male)	85	30	NSCLC	OS
Zhang et al	2014	China	Median 64	Mixed (65.7% male)	70	36	Lung cancer	OS
Abrao et al	2015	Brazil	59.6 (11.8)	Mixed (29.07% male)	86	1	Mixed (lung, breast, gastrointestinal, and others)	OS
Gkiozos et al	2015	Greece	NA	Mixed (75.2% male)	40	44	NSCLC	OS
								PFS
Porcel et al	2015	Spain	58–78	Mixed (77% male)	556	30	Lung cancer	OS
Xu et al	2015	China	58.3 ± 13.7	Mixed (54.08% male)	98	100	Lung cancer	OS
Zamboni et al	2015	Brazil	60.0 (1.0–95.0)	Mixed (47% male)	165	100	Mixed (ovary, breast, lymphoma, lung, and others)	OS
Zhao et al	2015	China	NA	Mixed (48.8% male)	43	30	Lung adenocarcinoma	PFS
Abrao et al	2016	Brazil	60 (24–86)	Mixed (31.25% male)	64	6	Mixed (lung, breast, gastrointestinal, and others)	OS
Hsu et al	2016	China	Median 57	Mixed (59% male)	61	59	Mixed (lung, breast, and others)	OS
Kasapoglu et al	2016	Turkey	64 (30–85)	Mixed (76% male)	199	60	Lung cancer	OS
Psallidas et al	2016	UK	NA	NA	75	28	Mixed (not specified)	OS
Tamiya et al	2016	Greece	68 (49–83)	Mixed (69.9% male)	23	47	NSCLC	OS
								PFS
Terra et al	2016	USA	58.9 ± 12	Mixed (28.21% male)	156	40	Mixed (breast, lung, lymphoma, and others)	OS
Usui et al	2016	Japan	NA	Mixed (80% male)	25	50	NSCLC	OS
								PFS
Verma et al	2016	Singapore	71 (38–92)	Mixed (51% male)	71	49	Lung adenocarcinoma	OS
Yang et al	2016	China	38–75	Mixed (48.7% male)	78	32	Lung cancer	OS
Amn et al	2017	Indonesia	17–85	Mixed (44% male)	102	27	Mixed (lung, breast, lymphoma, and others)	OS
Lee et al	2017	South Korea	NA	Mixed (51.3% male)	158	72	Lung cancer	OS
Lu et al	2017	China	59.34 ± 1.56	Mixed (65.71% male)	70	264	NSCLC	OS
Yang et al	2017	Korea	71 (42–94)	Mixed (37.5% male)	40	40	Lung adenocarcinoma	PFS
Zheng et al	2017	China	NA	Mixed (46.1% male)	128	55	NSCLC	OS
								PFS
Abisheganaden et al	2018	Singapore	72(38–92)	Mixed (53% male)	70	20	Lung adenocarcinoma	OS
Elena et al	2018	Spain	61.6 ± 11.2	Mixed (56% male)	84	14	Mixed (breast, mesothelioma, and lung cancer)	OS
Han et al	2018	Korea	70 ± 11	Mixed (65% male)	131	84	Mixed (lung, breast, ovary, lymphoma, and others)	OS
Jeba et al	2018	India	median 53	Mixed (29% male)	48	70	Mixed (lung, breast, gastrointestinal, and others)	OS
Lim et al	2018	Korea	68(35–92)	Mixed (55.3% male)	217	32	NSCLC	OS
								PFS
Porcel et al	2018	Spain	52–76	Mixed (57% male)	24	70	diffuse large B-cell lymphomas	OS
Psallidas et al	2018	UK	NA	NA	232	133	MPE	OS
Wu et al	2018	China	44(28–50)	Mixed (51% male)	142	45	Lung adenocarcinoma	OS
Xu et al	2018	China	62(40–78)	Mixed (50% male)	40	43	MPM	OS
Foote et al	2019	USA	61.44 ± 15.36	Mixed (41% male)	686	83	Mixed (lung, breast, gynecologic, lymphoma, and others)	OS
Kleontas et al	2019	United Kingdom	61.0 ± 10.9	Mixed (92.5% male)	40	41	Lung cancer	OS
Tian et al	2019	China	59.7 ± 9.12	Mixed (46% male)	160	25	Mixed (lung, breast, esophageal, gastric, and mesothelioma)	OS
Wang et al	2019	China	NA	Mixed (88% male)	295	84	Lung adenocarcinoma	OS
Martin et al	2020	UK	71(69–74)	Mixed (67% male)	97	75	Mixed (mesothelioma, lung, breast, genitourinary, and others)	OS
Quek et al	2020	Singapore	65(56–71)	Mixed (59% male)	130	14	Mixed (lung, mesothelioma, breast, gastrointestinal, and others)	OS
Shi et al	2020	China	65(55–73)	Mixed (51.3% male)	193	65	Mixed (lung, mesothelioma, and others)	OS
Stockhammer et al	2020	Germany	73.7 ± 8.6	Mixed (88% male)	48	40	MPM	OS

*NA* not available, *NSCLC* non-small cell lung cancer, *MPM* malignant pleural mesothelioma, *OS* overall survival, *PFS* progression-free survival

### Quality assessment of individual studies

The quality of each study was assessed in accordance with the Newcastle–Ottawa Scale (NOS) and was summarized in Additional file 1: eTable 2. The NOS score of all involved studies were above 6, which indicate low risk of bias.

### Included biomarkers

To comprehensively analyze the prognostic factors of malignant patients with pleural effusion, we included all biomarkers with the number of studies more than three. The clinical parameter biomarkers of OS include age, gender, smoking status, ECOG PS, stage, histology, cytology, distant metastasis, EGFR mutation and LENT score. The clinical parameter biomarkers of PFS include age, gender, smoking status, ECOG PS, stage and EGFR mutation. In addition, many studies analyzed the serum and pleural effusion biomarkers in the prognostic value of malignant patients with pleural effusion. The serum biomarkers include white blood cell counts (WBC), NLR, hemoglobin, total protein, albumin, LDH, C-reactive protein (CRP) and vascular endothelial growth factor (VEGF). The pleural effusion biomarkers include neutrophils, PH, total protein, albumin, glucose, LDH, VEGF and survivin.

### Clinical parameter biomarkers for malignant patients with pleural effusion, primary outcome: OS

In studies that reported the OS as outcome and evaluated the prognostic value of clinical parameter biomarkers, 36 were about the prognostic value of age, 35 of gender, 18 of smoking status, 28 of ECOG PS, 14 of stage, 17 of histology, 7 of cytology, 11 of distant metastasis, 7 of EGFR mutation and 8 of LENT score.

**Fig. 2:**
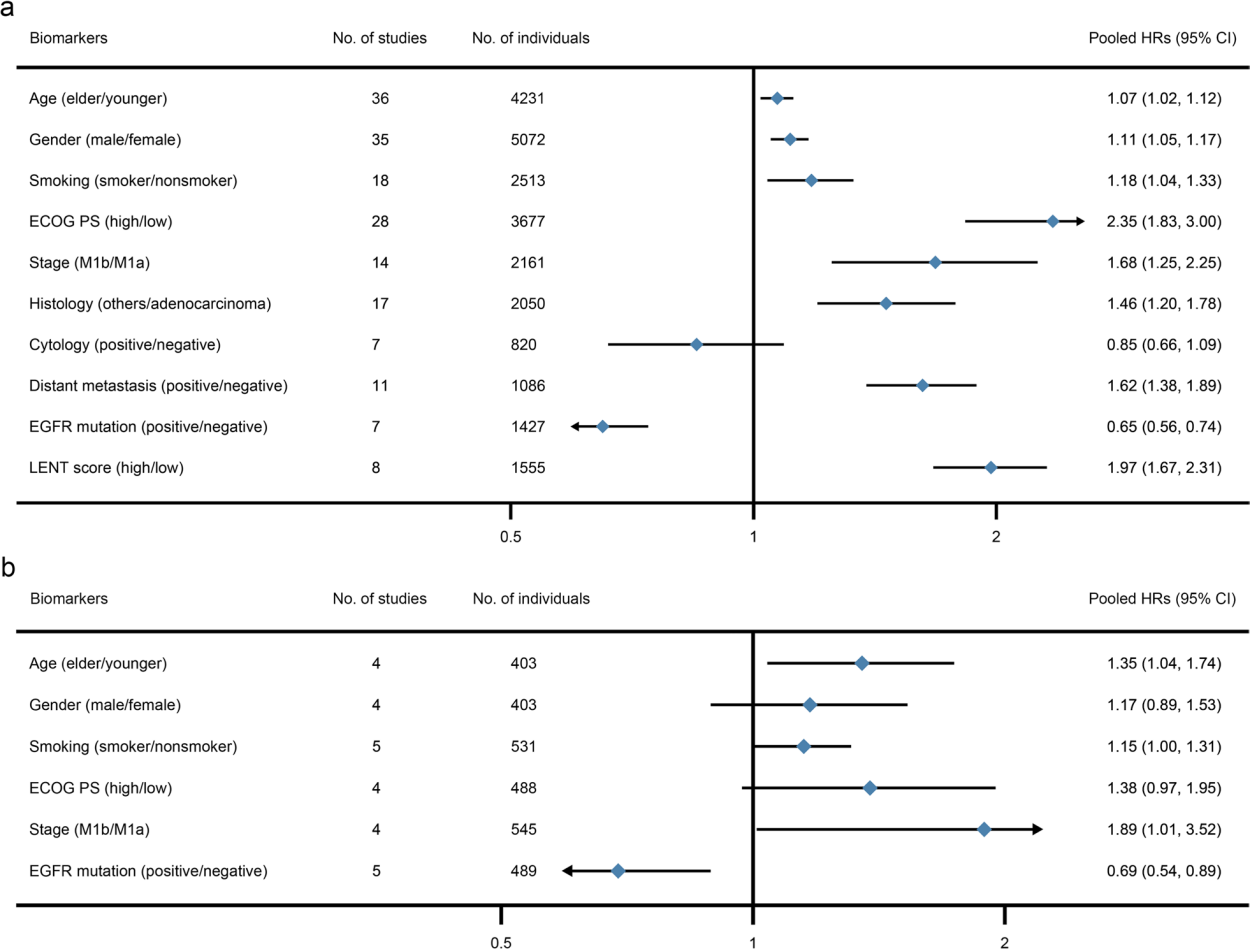
Pooled HRs of the clinical parameters in prognostic value of OS (**a**) and PFS (**b**) in malignant patients with pleural effusion

Figure 2a shows the forest plot of pooled HRs for the prognostic value of demographic data in OS with 95% CI. The pooled data demonstrate that elder age (HR 1.07; 95% CI 1.02–1.12), male gender (HR 1.11; 95% CI 1.05–1.17), smokers (HR 1.18; 95% CI 1.04–1.33), high ECOG PS (HR 2.35; 95% CI 1.83–3.00), M1b stage (HR 1.68; 95% CI 1.25–2.25), non-adenocarcinoma (HR 1.46; 95% CI 1.20–1.78), positive distant metastasis (HR 1.62; 95% CI 1.38–1.89) and high LENT score (HR 1.97; 95% CI 1.67–2.31) are prognostic risk factor in OS for malignant patients with pleural effusion. On the other hand, positive EGFR mutation is a protective factor in OS for malignant patients with pleural effusion (HR 0.65; 95% CI 0.56–0.74). The forest plots of each biomarker are showed on Additional file 1: eFigure 1–10. Heterogeneity testing revealed heterogeneity (*I*^2^ > 50%) in age, smoking status, ECOG PS, stage and histology.

### ***Clinical parameter biomarkers for malignant patients with pleural effusion, secondary outcome: PFS***.

In studies that reported the PFS as outcome and evaluated the prognostic value of clinical parameters, 4 were about the prognostic value of age, 4 of gender, 5 of smoking status, 4 of ECOG PS, 4 of stage and 5 of EGFR mutation.

Figure 2b shows the forest plot of pooled HRs for the prognostic value of demographic data in PFS with 95% CI. The pooled data demonstrate that elder age (HR 1.35; 95% CI 1.04–1.74), smokers (HR 1.15; 95% CI 1.00–1.31) and M1b stage (HR 1.89; 95% CI 1.01–3.52) are prognostic risk factors in PFS for malignant patients with pleural effusion. Positive EGFR mutation is still a protective factor in PFS for lung adenocarcinoma patients with pleural effusion (HR 0.69; 95% CI 0.54–0.89). The forest plots of each biomarker are showed on Additional file 1: eFigure 11–16. Heterogeneity testing revealed heterogeneity (*I*^2^ > 50%) in ECOG PS and stage.

### Serum prognostic biomarkers for malignant patients with pleural effusion

Serum prognostic biomarkers has been widely studied in the few decades and we systematically summarized these studies. In these studies, 4 were about the prognostic value of WBC, 5 of NLR, 4 of hemoglobin, 3 of total protein, 7 of albumin, 6 of LDH, 4 of CRP and 4 of VEGF.

**Fig. 3:**
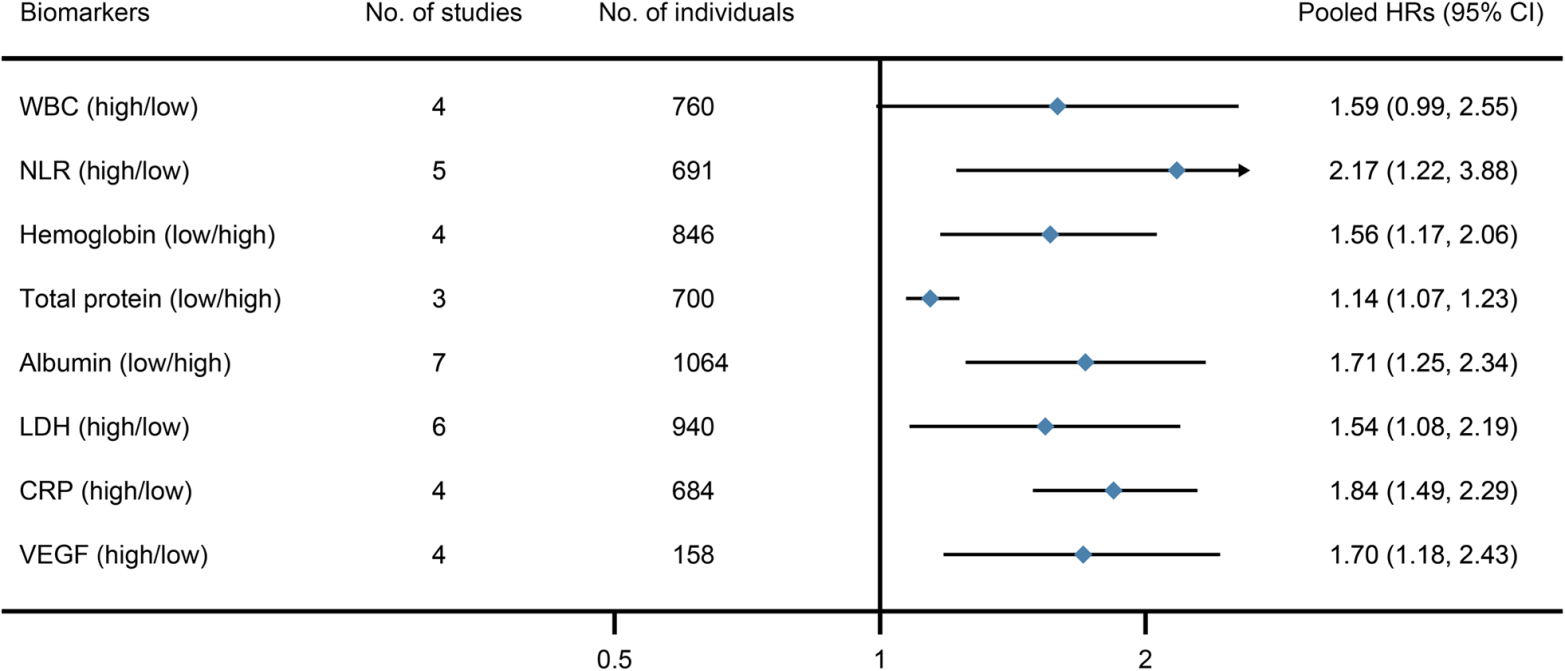
Pooled HRs of the serum biomarkers in prognostic value of OS in malignant patients with pleural effusion

The pooled data of forest plot is showed in Fig. 3. The results demonstrate that high NLR (HR 2.17; 95% CI 1.22–3.88), low hemoglobin (HR 1.56; 95% CI 1.17–2.06), low total protein (HR 1.14; 95% CI 1.07–1.23), low albumin (HR 1.71; 95% CI 1.25–2.34), high LDH (HR 1.54; 95% CI 1.08–2.19), high CRP (HR 1.84; 95% CI 1.49–2.29) and high VEGF (HR 1.70; 95% CI 1.18–2.43) in serum are prognostic risk factors in OS for malignant patients with pleural effusion. In addition, serum VEGF is also a prognostic biomarker associated with a poor prognosis in PFS for malignant patients with pleural effusion (HR 1.70; 95% CI 1.00–2.89). The forest plots of each biomarker in serum are showed on Additional file 1: eFigure 17–25. Heterogeneity testing revealed heterogeneity (*I*^*2*^ > 50%) in WBC, NLR, albumin, LDH, and VEGF for PFS.

### Pleural effusion prognostic biomarkers for malignant patients

Pleural effusion biomarkers are always the best material to test the pleural diseases. We also summarized the pleural effusion prognostic biomarkers for malignant patients. In these studies, 3 were about the prognostic value of neutrophils, 12 of PH, 10 of total protein, 4 of albumin, 12 of glucose, 14 of LDH, 10 of VEGF and 5 of survivin.

**Fig. 4:**
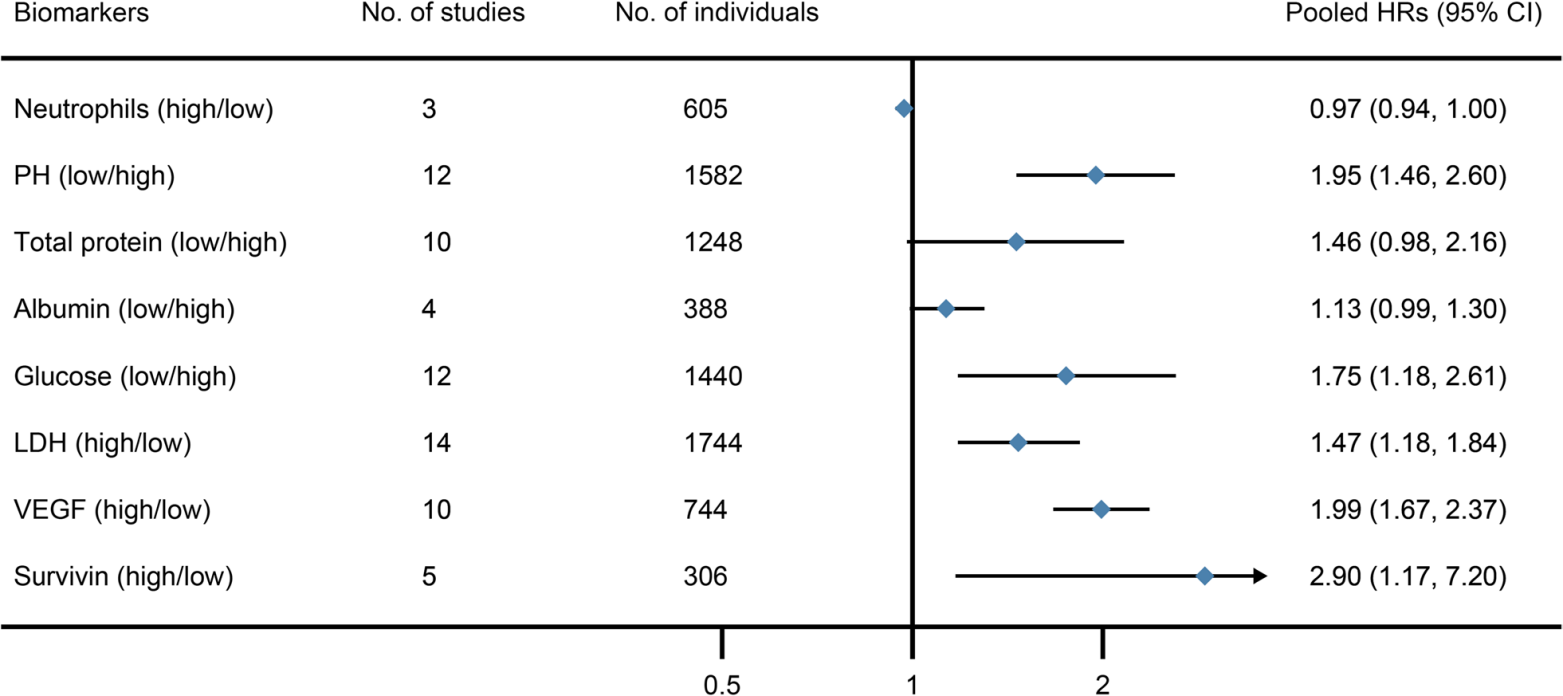
Pooled HRs of the pleural effusion biomarkers in prognostic value of OS in malignant patients with pleural effusion

The pooled data of forest plot is showed in Fig. 4. The results demonstrate that low PH (HR 1.95; 95% CI 1.46–2.60), low glucose (HR 1.75; 95% CI 1.18–2.61), high LDH (HR 1.47; 95% CI 1.18–1.84), high VEGF (HR 1.99; 95% CI 1.67–2.37) and high survivin (HR 2.90; 95% CI 1.17–7.20) in pleural effusion are prognostic risk factors in OS for malignant patients. In addition, pleural effusion VEGF is also a prognostic biomarker associated with a poor prognosis in PFS for malignant patients with pleural effusion (HR 1.42; 95% CI 1.02–2.00). The forest plots of each biomarker in serum are showed on Additional file 1: eFigure 26–34. Heterogeneity testing revealed heterogeneity (*I*^*2*^ > 50%) in PH, total protein, glucose, LDH, VEGF for PFS and survivin.

### Subgroup analysis

Significant heterogeneities were observed for the prognostic significance of age (I^2^ = 58.9%; P < 0.001), smoking status (I^2^ = 64.7%; P < 0.001), ECOG PS (I^2^ = 83.5%; P < 0.001), stage (I^2^ = 87.5%; P < 0.001), histology (I^2^ = 70.4%; P < 0.001), serum WBC (I^2^ = 87.7%; P < 0.001), serum NLR (I^2^ = 87.7%; P < 0.001), serum albumin (I^2^ = 61.2%; P < 0.05), pleural effusion PH (I^2^ = 88.5%; P < 0.001), pleural effusion total protein (I^2^ = 81.0%; P < 0.001), pleural effusion glucose (I^2^ = 86.2%; P < 0.001), pleural effusion LDH (I^2^ = 70.5%; P < 0.001), pleural effusion VEGF (I^2^ = 48.7%; P < 0.05) and pleural effusion surviving (I^2^ = 83.3%; P < 0.001) for overall survival in malignant patients with pleural effusion. For progression-free survival, significant heterogeneities were observed for the prognostic significance of stage (I^2^ = 87.8%; P < 0.001), smoking status (I^2^ = 64.7%; P < 0.001), ECOG PS (I^2^ = 83.5%; P < 0.001), stage (I^2^ = 87.5%; P < 0.001), histology (I^2^ = 70.4%; P < 0.001) and serum VEGF (I^2^ = 80.7%; P = 0.001) in malignant patients with pleural effusion. Thus, subgroup analyses were performed by categorizing subgroups by cancer types. As shown in Table 1, caner types in half of the studies were not specifically reported. In the subgroup analyses of the variables, there were no significant associations among cancer types (test for subgroup differences: P > 0.05) (Additional file 1: eFigure 35–41).

### Sensitivity analysis

In order to find the source of heterogeneity, sensitivity analyses were performed in all prognostic biomarkers with *I*^*2*^ > 50% (Additional file 1: eFigure 42–59). Two studies (Burrows et al [15] and Özyurtkan et al [16]) significantly influence the pooled result of pleural effusion glucose for OS in malignant patients. We have excluded these two studies for the glucose pooled result.

### Publication bias

We analyzed all biomarkers which include more than 10 studies. The contour-enhanced meta-analysis funnel plot of HR is presented in Additional file 1: eFigure 60–71. Publication bias was present in age, stage, histology, PH of pleural and LDH in pleural effusion with the Egger tests’ p value < 0.05.

## Discussion

Pleural effusion is a common clinical problem in patients with cancer, and may be due to both primary thoracic tumor or to a metastatic spread in the chest and constitutes the first sign of disease in approximately 10% of patients [17]. This study systematically summarizes all possible prognostic factors of pleural effusion caused by malignancy and may give a hind about the treatment and prognosis of malignant patients. Our main findings indicate that except the common prognostic factors such as elder age, male gender, smoking status, more advanced disease and distant metastasis, many other indicators can be valuable prognostic factors for pleural effusion caused by malignancy. These indicators include the clinical parameters such as ECOG PS, non-adenocarcinoma histology, EGFR mutation and LENT score, the serum indicators such as NLR, hemoglobin, total protein, albumin, LDH, CRP and VEGF, and the pleural effusion indicators such as PH, glucose, LDH, VEGF and surviving.

A multi-marker strategy may be a much better approach in predicting MPE prognosis. LENT score is one of the most widely recognized scoring systems to predict survival in patients with malignant pleural effusion, calculated based on tumor type, ECOG PS, serum NLR, and pleural fluid LDH. Our study also confirms its effectiveness that LENT score shows an excellent prognostic value for malignant patients with pleural effusion, so do the LENT score calculation items (LDH, ECOG PS, NLR and tumor type). However, the LENT score has not included many important developments in the prognosis of pleural effusion [18]. In our study, positive EGFR mutation patients shows a better survival both in OS and PFS. As we know that lung adenocarcinoma with malignant pleural effusion is associated with a higher incidence of EGFR mutations [19], Abisheganaden et al. advice to modify the LENT score with EGFR mutation in lung adenocarcinoma patients [9]. The LENT score system was created as a robust prognostic score in order to aid in decision-making regarding treatment of the diverse populations of patients with malignant [7], so further modifications according to other prognostic factors may be needed to provide a better prognostic effect. The SELECT prognostication model, which included EGFR mutations, has recently been recognized as a more effective model for predicting survival in Asian MPE populations, and more studies are needed to evaluate the accuracy of this scoring system. Many biological parameters also show the prognostic value for MPE such as hemoglobin, albumin, CRP and VEGF in our study. PROMISE score combines these biological parameters and clinical parameters to accurately estimate 3-month mortality [8]. This score includes pleural fluid tissue inhibitor of metalloproteinases as one of the evaluate indexes. Unfortunately, there is not enough study of this parameter for us to analyze.

Except the LENT score, modified LENT score, SELECT model and PROMISE score items, we also found that serum albumin, serum and pleural effusion VEGF, pleural effusion PH, pleural effusion glucose and pleural effusion survivin are also valuable prognostic factors for malignant patients. VEGF is a potent angiogenic regulator with a crucial role in the initiation and progression of solid malignancies [20, 21] and MPE is associated with high levels of VEGF in serum and MPE [22, 23]. According to our analysis, the increased VEGF levels in pleural fluid and serum may associated with worse OS and PFS. In addition, pleural fluid analysis has not only diagnostic but also prognostic significance in patients with malignant effusion [24]. Our results have confirmed this view. These indictors provide a new direction for the prognosis of malignant patients with pleural effusion.

Neuron-specific enolase (NSE) [25], Cancer Antigen 153 (CA153) and Cancer Antigen 125 (CA125) [26] are validated for the diagnosis and prognosis of patients with cancer. However, there is still a lack of research on whether these biomarkers in pleural effusion have prognostic values on MPE patients. The prognosis value of other combined use of markers such as Cancer ratio [27] and Cancer ratio plus [28] in predicting survival of MPE should also be studied.

Our study has some limitations. First, the combinations of some factors have significant heterogeneity. The heterogeneity may come from the different types of cancer. Second, some of the funnel plots implied possible publication bias. We have already tried to include all studies that meet the criteria, but the publication bias cannot be avoided. Third, for the outcomes, only OS and PFS were involved. Although some studies demonstrate the disease-free survival, the number is too small to summarize.

## Conclusions

Our findings suggest that for malignant patients with pleural effusion, LENT score and its items are valuable prognostic biomarkers, so do the clinical parameters like stage, distant metastasis, EGFR mutation, the serum biological parameters like hemoglobin, albumin, C-reaction protein, VEGF, and the pleural effusion biological parameters like PH, glucose, VEGF and survivin.

## Supplementary Information

**Additional file 1**. Original study protocol, supplementary tables and supplementary figures.

## Data Availability

Data sharing is not applicable to this article as no datasets were generated or analyzed during the current study.

## Abbreviations

MPE: Malignant pleural effusions
LDH: Lactate dehydrogenase
ECOG PS: Eastern Cooperative Oncology Group Performance Status
NLR: Neutrophil-to-lymphocyte ratio
PRISMA: Preferred Reporting Items for Systematic Reviews and Meta-Analysis
OS: Overall survival
PFS: Progression-free survival
HR: Hazard ratio
NOS: Newcastle–Ottawa Scale
WBC: White blood cell counts
CRP: C-reactive protein
VEGF: Vascular endothelial growth factor
