# Supplementary material for: Prognostic biomarkers of malignant patients with pleural effusion: a systematic review and meta-analysis
Source: Cancer Cell Int. 2022 Feb 24;22:99. doi: 10.1186/s12935-022-02518-w (PMC8876446; doi:10.1186/s12935-022-02518-w)
Supplement: Supplementary file 1 — Additional file 1. Original study protocol, supplementary tables and supplementary figures. [file 12935_2022_2518_MOESM1_ESM.docx]

**Original study protocol**

**Prognostic biomarkers of malignant patients with pleural effusion (Protocol)**

Peng Peng, MD; Yuan Yang, MD; Juan Du, MM; Kan Zhai, MD; Huan-Zhong Shi, MD

**Abstract**

The objectives of this systematic review and meta-analysis are as follows:

To summarize all the known prognostic indicators of malignant patients with pleural effusion.

**Background**

Pleural effusions are common medical problems resulting from the increasing fluid in the pleural space. More than 50 diseases can cause pleural effusion, such as pleura disease, lung disease, organ dysfunction, systemic conditions and drugs. Malignant pleural effusion is one of the most common types of pleural effusion. It can be observed in many malignant diseases such as lung cancer, breast cancer, ovarian cancer and so on. The occurrence of pleural effusion in patients with malignancy always indicates the cancer systemic dissemination, thereupon then declines the life expectancy and quality. Identifying the prognostic biomarkers can provide important information on the survival of patients and guide the selection of management strategies. So, we will do a systematic review and meta-analysis to summarize all the known prognostic indicators of malignant patients with pleural effusion.

**Objectives**

To summarize all the known prognostic indicators of malignant patients with pleural effusion.

**Criteria for considering studies for this review**

***Types of studies***

Prospective or retrospective cohort studies.

***Types of participants***

Inclusion: Adults with any form of malignant tumor and pleural effusion.

Exclusion: Adolescents (under 18 years of age).

Exposures: Any kind of prognostic biomarkers of malignant patients with pleural effusion.

Control: A group of patients without the prognostic biomarkers.

***Types of outcome measures***

The main outcome was death due to all causes during follow up. The outcome measurements include overall survival and progression-free survival.

**Identification of studies**

We will do a systematic review and meta-analysis to summarize all the known prognostic indicators of malignant patients with pleural effusion. We will attempt to identify all relevant trials, irrespective of language or publication status. We will search several electronic databases, covering all dates from the creation of each database up to April 2, 2020: PubMed, the Cochrane Library, Medline, Embase, and Web of Science.

**Methods of review**

***Study selection***

Two group reviewers (PP and YY plus DJ and ZK) will independently search the databases and selected the citations according to the search strategy. YY and DJ will read full articles of the potentially relevant papers and reasons of excluded studies will be given. Disagreements will be resolved by discussion. When the papers do not give enough information, we will contact the authors.

***Assessment of methodological quality***

The quality of each study will be assessed in accordance with the Newcastle-Ottawa Scale (NOS). The scoring will be based on the following items: representativeness of exposed cohort, selection of non-exposed cohort, ascertainment of exposure, demonstration that outcome of interest was not present at start of study, comparability of cohorts on the basis of the design or analysis, assessment of outcome, was follow-up long enough for outcomes to occur and adequacy of follow up of cohorts. Two reviews (YY and DJ) will independently assess the risk of bias of each trial. They will cross check the data and settle discrepancies by discussion.

***Data extraction***

We will develop a data extraction sheet (based on the Cochrane Consumers and Communication Review Group’s data extraction template). Two reviews (YY and DJ) will independently extract data with the data extraction sheet. They cross checked the data and disagreements were resolved by discussion.

***Data analysis***

We will use the Mantel-Haenszel method to calculate the pooled HR and 95% CI. Data will be graphically displayed using forest plots. *I^2^* test will be used to detect heterogeneity. Sensitivity analyses will be used to find the source of heterogeneity. The funnel plots and contour-enhanced meta-analysis funnel plots will be used to examine the publication bias. A two-sided P value < 0 .05 will be considered statistically significant. The data analyses will be performed using software Stata (version 15; <https://www.stata.com/).>

**Studies included in this systematic review and meta-analysis ^1-82^**

1. Sahn SA, Good JT, Jr. Pleural fluid pH in malignant effusions. Diagnostic, prognostic, and therapeutic implications. *Ann Intern Med* 1988; **108**(3): 345-9.

2. Rodriguez-Panadero F, Lopez-Mejias J. Survival time of patients with pleural metastatic carcinoma predicted by glucose and pH studies. *Chest* 1989; **95**(2): 320-4.

3. Foresti V, Scolari N, Villa A, Parisio E, De Filippi G, Guareschi G. Malignant pleural effusions: meaning of pleural-fluid pH determination. *Oncology* 1990; **47**(1): 62-4.

4. Gottehrer A, Taryle DA, Reed CE, Sahn SA. Pleural fluid analysis in malignant mesothelioma. Prognostic implications. *Chest* 1991; **100**(4): 1003-6.

5. Sugiura S, Ando Y, Minami H, Ando M, Sakai S, Shimokata K. Prognostic value of pleural effusion in patients with non-small cell lung cancer. *Clin Cancer Res* 1997; **3**(1): 47-50.

6. Martínez-Moragón E, Aparicio J, Sanchis J, Menéndez R, Cruz Rogado M, Sanchis F. Malignant pleural effusion: prognostic factors for survival and response to chemical pleurodesis in a series of 120 cases. *Respiration* 1998; **65**(2): 108-13.

7. Burrows CM, Mathews C, Colt HG. Predicting survival in patients with recurrent symptomatic malignant pleural effusions - An assessment of the prognostic values of physiologic, morphologic, and quality of life measures of extent of disease. *Chest* 2000; **117**(1): 73-8.

8. Heffner JE, Nietert PJ, Barbieri C. Pleural fluid pH as a predictor of survival for patients with malignant pleural effusions. *Chest* 2000; **117**(1): 79-86.

9. Chen YM, Yang WK, Whang-Peng J, Tsai CM, Perng RP. An analysis of cytokine status in the serum and effusions of patients with tuberculous and lung cancer. *Lung Cancer* 2001; **31**(1): 25-30.

10. Thylén A, Hjerpe A, Martensson G. Hyaluronan content in pleural fluid as a prognostic factor in patients with malignant pleural mesothelioma. *Cancer* 2001; **92**(5): 1224-30.

11. Bernard A, de Dompsure RB, Hagry O, Favre JP. Early and late mortality after pleurodesis for malignant pleural effusion. *Ann Thorac Surg* 2002; **74**(1): 213-7.

12. Eitan R, Levine DA, Abu-Rustum N, Sonoda Y, Huh JN, Franklin CC, et al. The clinical significance of malignant pleural effusions in patients with optimally debulked ovarian carcinoma. *Cancer* 2005; **103**(7): 1397-401.

13. Aelony Y, Yao JF, King RR. Prognostic value of pleural fluid pH in malignant epithelial mesothelioma after talc poudrage. *Respiration* 2006; **73**(3): 334-9.

14. Aoe K, Hiraki A, Yamazaki K, Nakamura Y, Murakami T, Maeda T, et al. Elevated pleural fluid RCAS1 is a diagnostic marker and outcome predictor in lung cancer patients. *Int J Oncol* 2006; **29**(1): 65-72.

15. Soh J, Toyooka S, Aoe K, Asano H, Ichihara S, Katayama H, et al. Usefulness of EGFR mutation screening in pleural fluid to predict the clinical outcome of gefitinib treated patients with lung cancer. *Int J Cancer* 2006; **119**(10): 2353-8.

16. Bielsa S, Salud A, Martínez M, Esquerda A, Martín A, Rodríguez-Panadero F, et al. Prognostic significance of pleural fluid data in patients with malignant effusion. *Eur J Intern Med* 2008; **19**(5): 334-9.

17. Wu SG, Gow CH, Yu CJ, Chang YL, Yang CH, Hsu YC, et al. Frequent epidermal growth factor receptor gene mutations in malignant pleural effusion of lung adenocarcinoma. *Eur Respir J* 2008; **32**(4): 924-30.

18. Hsu IL, Su WC, Yan JJ, Chang JM, Lai WW. Angiogenetic biomarkers in non-small cell lung cancer with malignant pleural effusion: correlations with patient survival and pleural effusion control. *Lung Cancer* 2009; **65**(3): 371-6.

19. Wu YK, Chen KT, Kuo YB, Huang YS, Chan EC. Quantitative detection of survivin in malignant pleural effusion for the diagnosis and prognosis of lung cancer. *Cancer Lett* 2009; **273**(2): 331-5.

20. Kotyza J, Havel D, Vrzalová J, Kulda V, Pesek M. Diagnostic and prognostic significance of inflammatory markers in lung cancer-associated pleural effusions. *Int J Biol Markers* 2010; **25**(1): 12-20.

21. Lan CC, Wu YK, Lee CH, Huang YC, Huang CY, Tsai YH, et al. Increased survivin mRNA in malignant pleural effusion is significantly correlated with survival. *Jpn J Clin Oncol* 2010; **40**(3): 234-40.

22. Ozyurtkan MO, Balci AE, Cakmak M. Predictors of mortality within three months in the patients with malignant pleural effusion. *Eur J Intern Med* 2010; **21**(1): 30-4.

23. Pilling JE, Dusmet ME, Ladas G, Goldstraw P. Prognostic factors for survival after surgical palliation of malignant pleural effusion. *J Thorac Oncol* 2010; **5**(10): 1544-50.

24. Tanrikulu AC, Abakay A, Kaplan MA, Küçüköner M, Palanci Y, Evliyaoglu O, et al. A clinical, radiographic and laboratory evaluation of prognostic factors in 363 patients with malignant pleural mesothelioma. *Respiration* 2010; **80**(6): 480-7.

25. Hirayama N, Tabata C, Tabata R, Maeda R, Yasumitsu A, Yamada S, et al. Pleural effusion VEGF levels as a prognostic factor of malignant pleural mesothelioma. *Respir Med* 2011; **105**(1): 137-42.

26. Sakr L, Maldonado F, Greillier L, Dutau H, Loundou A, Astoul P. Thoracoscopic assessment of pleural tumor burden in patients with malignant pleural effusion: prognostic and therapeutic implications. *J Thorac Oncol* 2011; **6**(3): 592-7.

27. Yamada S, Tabata C, Tabata R, Fukuoka K, Nakano T. Clinical significance of pleural effusion mesothelin in malignant pleural mesothelioma. *Clin Chem Lab Med* 2011; **49**(10): 1721-6.

28. Botana-Rial M, De Chiara L, Valverde D, Leiro-Fernández V, Represas-Represas C, Del Campo-Pérez V, et al. Prognostic value of aberrant hypermethylation in pleural effusion of lung adenocarcinoma. *Cancer Biol Ther* 2012; **13**(14): 1436-42.

29. Guo H, Wan Y, Tian G, Liu Q, Kang Y, Li Y, et al. EGFR mutations predict a favorable outcome for malignant pleural effusion of lung adenocarcinoma with Tarceva therapy. *Oncol Rep* 2012; **27**(3): 880-90.

30. Hooper CE, Elvers KT, Welsh GI, Millar AB, Maskell NA. VEGF and sVEGFR-1 in malignant pleural effusions: association with survival and pleurodesis outcomes. *Lung Cancer* 2012; **77**(2): 443-9.

31. Park DS, Hwang KE, Shim H, Kim BR, Choi KH, Park SH, et al. Elevated survivin is associated with a poor response to chemotherapy and reduced survival in lung cancer with malignant pleural effusions. *Clin Exp Metastasis* 2012; **29**(2): 83-9.

32. Qian Q, Sun WK, Zhan P, Zhang Y, Song Y, Yu LK. Role of monocyte chemoattractant protein-1, tumor necrosis factor-alpha and interleukin-6 in the control of malignant pleural effusion and survival in patients with primary lung adenocarcinoma. *Int J Biol Markers* 2012; **27**(2): e118-24.

33. Qian Q, Zhan P, Sun WK, Zhang Y, Song Y, Yu LK. Vascular endothelial growth factor and soluble intercellular adhesion molecule-1 in lung adenocarcinoma with malignant pleural effusion: correlations with patient survival and pleural effusion control. *Neoplasma* 2012; **59**(4): 433-9.

34. Wang T, Lv M, Shen S, Zhou S, Wang P, Chen Y, et al. Cell-free microRNA expression profiles in malignant effusion associated with patient survival in non-small cell lung cancer. *PLoS One* 2012; **7**(8): e43268.

35. Cheng D, Kong H, Li Y. Prognostic values of VEGF and IL-8 in malignant pleural effusion in patients with lung cancer. *Biomarkers* 2013; **18**(5): 386-90.

36. Faiz SA, Bashoura L, Lei X, Sampat KR, Brown TC, Eapen GA, et al. Pleural effusions in patients with acute leukemia and myelodysplastic syndrome. *Leuk Lymphoma* 2013; **54**(2): 329-35.

37. Görgün D, Seçik F, Midilli K, Akkaya V, Yıldız P. Diagnostic and prognostic significance of survivin levels in malignant pleural effusion. *Respir Med* 2013; **107**(8): 1260-5.

38. Park DS, Kim D, Hwang KE, Hwang YR, Park C, Seol CH, et al. Diagnostic value and prognostic significance of pleural C-reactive protein in lung cancer patients with malignant pleural effusions. *Yonsei Med J* 2013; **54**(2): 396-402.

39. Wu SG, Yu CJ, Tsai MF, Liao WY, Yang CH, Jan IS, et al. Survival of lung adenocarcinoma patients with malignant pleural effusion. *Eur Respir J* 2013; **41**(6): 1409-18.

40. Anevlavis S, Kouliatsis G, Sotiriou I, Koukourakis MI, Archontogeorgis K, Karpathiou G, et al. Prognostic factors in patients presenting with pleural effusion revealing malignancy. *Respiration* 2014; **87**(4): 311-6.

41. Clive AO, Kahan BC, Hooper CE, Bhatnagar R, Morley AJ, Zahan-Evans N, et al. Predicting survival in malignant pleural effusion: development and validation of the LENT prognostic score. *Thorax* 2014; **69**(12): 1098-104.

42. Xu C, Yu L, Zhan P, Zhang Y. Elevated pleural effusion IL-17 is a diagnostic marker and outcome predictor in lung cancer patients. *Eur J Med Res* 2014; **19**(1): 23.

43. Zhang H, Liu HB, Yuan DM, Wang ZF, Wang YF, Song Y. Prognostic value of secreted phosphoprotein-1 in pleural effusion associated with non-small cell lung cancer. *BMC Cancer* 2014; **14**: 280.

44. Zhang Y, Yu LK, Lu GJ, Xia N, Xie HY, Hu W, et al. Prognostic values of VEGF and endostatin with malignant pleural effusions in patients with lung cancer. *Asian Pac J Cancer Prev* 2014; **15**(19): 8435-40.

45. Abrao FC, de Abreu IR, Fogarolli M, Caxeiro G, Bezerra CB, de Cerqueira Cesar FP, et al. Prognostic Factors of 30-Day Mortality After Palliative Procedures in Patients with Malignant Pleural Effusion. *Ann Surg Oncol* 2015; **22**(12): 4083-8.

46. Gkiozos I, Tsagouli S, Charpidou A, Grapsa D, Kainis E, Gratziou C, et al. Levels of vascular endothelial growth factor in serum and pleural fluid are independent predictors of survival in advanced non-small cell lung cancer: results of a prospective study. *Anticancer Res* 2015; **35**(2): 1129-37.

47. Ni XF, Wu P, Wu CP, Ji M, Wu J, Gu XF, et al. Elevated serum C-reactive protein, carcinoembryonic antigen and N2 disease are poor prognostic indicators in non-small cell lung cancer. *Asia Pac J Clin Oncol* 2015; **11**(4): e22-30.

48. Porcel JM, Gasol A, Bielsa S, Civit C, Light RW, Salud A. Clinical features and survival of lung cancer patients with pleural effusions. *Respirology* 2015; **20**(4): 654-9.

49. Xu CH, Cao L, Zhang XW, Yan J, Yu LK. Prognostic value of soluble H7-B4 in pleural effusion associated with lung cancer. *Tumour Biol* 2015; **36**(6): 4397-403.

50. Zamboni MM, da Silva CT, Jr., Baretta R, Cunha ET, Cardoso GP. Important prognostic factors for survival in patients with malignant pleural effusion. *BMC Pulm Med* 2015; **15**: 29.

51. Zhao C, Li X, Su C, Li J, Cheng N, Ren S, et al. High expression of E-cadherin in pleural effusion cells predicts better prognosis in lung adenocarcinoma patients. *Int J Clin Exp Pathol* 2015; **8**(3): 3104-9.

52. Abrao FC, Peixoto RD, de Abreu IR, Janini MC, Viana GG, de Oliveira MC, et al. Prognostic factors in patients with malignant pleural effusion: Is it possible to predict mortality in patients with good performance status? *J Surg Oncol* 2016; **113**(5): 570-4.

53. Hsu LH, Hsu PC, Liao TL, Feng AC, Chu NM, Kao SH. Pleural fluid osteopontin, vascular endothelial growth factor, and urokinase-type plasminogen activator levels as predictors of pleurodesis outcome and prognosticators in patients with malignant pleural effusion: a prospective cohort study. *BMC Cancer* 2016; **16**: 463.

54. Kasapoglu US, Arınç S, Gungor S, Irmak I, Guney P, Aksoy F, et al. Prognostic factors affecting survival in non-small cell lung carcinoma patients with malignant pleural effusions. *Clin Respir J* 2016; **10**(6): 791-9.

55. Psallidas I, Kannelakis N, Yousuf A, Corcoran J, Rahman N. Lent score validation on patients with malignant pleural effusion. *European Respiratory Journal* 2016; **48**(suppl 60): PA3385.

56. Tamiya M, Tamiya A, Yasue T, Nakao K, Omachi N, Shiroyama T, et al. Vascular Endothelial Growth Factor in Plasma and Pleural Effusion Is a Biomarker for Outcome After Bevacizumab plus Carboplatin-Paclitaxel Treatment for Non-small Cell Lung Cancer with Malignant Pleural Effusion. *Anticancer Res* 2016; **36**(6): 2939-44.

57. Terra RM, Antonangelo L, Mariani AW, de Oliveira RL, Teixeira LR, Pego-Fernandes PM. Pleural Fluid Adenosine Deaminase (ADA) Predicts Survival in Patients with Malignant Pleural Effusion. *Lung* 2016; **194**(4): 681-6.

58. Usui K, Sugawara S, Nishitsuji M, Fujita Y, Inoue A, Mouri A, et al. A phase II study of bevacizumab with carboplatin-pemetrexed in non-squamous non-small cell lung carcinoma patients with malignant pleural effusions: North East Japan Study Group Trial NEJ013A. *Lung Cancer* 2016; **99**: 131-6.

59. Verma A, Phua CK, Sim WY, Algoso RE, Tee KS, Lew SJ, et al. Pleural LDH as a prognostic marker in adenocarcinoma lung with malignant pleural effusion. *Medicine (Baltimore)* 2016; **95**(26): e3996.

60. Amin Z, Iskandar SD, Sibli. Prognostic Factors of 30-day Survival of Patients with Malignant Pleural Effusion. *Indian J Palliat Care* 2017; **23**(3): 321-4.

61. Lee YS, Nam HS, Lim JH, Kim JS, Moon Y, Cho JH, et al. Prognostic impact of a new score using neutrophil-to-lymphocyte ratios in the serum and malignant pleural effusion in lung cancer patients. *BMC Cancer* 2017; **17**(1): 557.

62. Lu GJ, Shao CJ, Zhang Y, Wei YY, Xie WP, Kong H. Diagnostic and prognostic values of endothelial-cell-specific molecule-1 with malignant pleural effusions in patients with non-small cell lung cancer. *Oncotarget* 2017; **8**(30): 49217-23.

63. Zheng Z, Xie D, Su H, Lin B, Zhao L, Deng X, et al. Treatment outcome comparisons between exons 19 and 21 EGFR mutations for non-small-cell lung cancer patients with malignant pleural effusion after first-line and second-line tyrosine kinase inhibitors. *Tumour Biol* 2017; **39**(6): 1010428317706211.

64. Abisheganaden J, Verma A, Dagaonkar RS, Light RW. An Observational Study Evaluating the Performance of LENT Score in the Selected Population of Malignant Pleural Effusion from Lung Adenocarcinoma in Singapore. *Respiration* 2018; **96**(4): 308-13.

65. Arellano-Orden E, Romero-Romero B, Sánchez-López V, Martín-Juan J, Rodríguez-Panadero F, Otero-Candelera R. Survivin is a negative prognostic factor in malignant pleural effusion. *Eur J Clin Invest* 2018; **48**(4).

66. Han AL, Kim H-R, Choi K-H, Ryu J-w, Hwang K-E, So H-S, et al. Expression Profile of Three Splicing Factors in Pleural Cells Based on the Underlying Etiology and Its Clinical Values in Patients with Pleural Effusion. *Translational Oncology* 2018; **11**(1): 147-56.

67. Jeba J, Cherian RM, Thangakunam B, George R, Visalakshi J. Prognostic Factors of Malignant Pleural Effusion among Palliative Care Outpatients: A Retrospective Study. *Indian J Palliat Care* 2018; **24**(2): 184-8.

68. Lim JU, Yeo CD, Kang HS, Park CK, Kim JS, Kim JW, et al. Prognostic value of platelet count and lymphocyte to monocyte ratio combination in stage IV non-small cell lung cancer with malignant pleural effusion. *PLoS One* 2018; **13**(7): e0200341.

69. Psallidas I, Kanellakis NI, Gerry S, Thézénas ML, Charles PD, Samsonova A, et al. Development and validation of response markers to predict survival and pleurodesis success in patients with malignant pleural effusion (PROMISE): a multicohort analysis. *Lancet Oncol* 2018; **19**(7): 930-9.

70. Wu SG, Liu YN, Yu CJ, Yang JC, Shih JY. Driver mutations of young lung adenocarcinoma patients with malignant pleural effusion. *Genes Chromosomes Cancer* 2018; **57**(10): 513-21.

71. Xu LL, Yang Y, Wang Z, Wang XJ, Tong ZH, Shi HZ. Malignant pleural mesothelioma: diagnostic value of medical thoracoscopy and long-term prognostic analysis. *BMC Pulm Med* 2018; **18**(1): 56.

72. Yang J, Lee OJ, Son SM, Woo CG, Jeong Y, Yang Y, et al. EGFR Mutation Status in Lung Adenocarcinoma-Associated Malignant Pleural Effusion and Efficacy of EGFR Tyrosine Kinase Inhibitors. *Cancer Res Treat* 2018; **50**(3): 908-16.

73. Yang J, Zhu Y, Wu L, Zhu W, Zhang X, Yang Y, et al. Diagnostic and prognostic significance of receptor-binding cancer antigen expressed on SiSo cells in lung-cancer-associated pleural effusion. *Clin Respir J* 2018; **12**(1): 279-84.

74. Foote DC, Burke CR, Pandian B, Banks S, Haug KL, Hipp M, et al. Gender Disparity in Referral for Definitive Care of Malignant Pleural Effusions. *J Surg Res* 2019; **244**(1095-8673 (Electronic)): 409-16.

75. Kleontas A, Sioga A, Pandria N, Barbetakis N, Lazopoulos A, Katsikas I, et al. Clinical factors affecting the survival of patients diagnosed with non-small cell lung cancer and metastatic malignant pleural effusion, treated with hyperthermic intrathoracic chemotherapy or chemical talc pleurodesis: a monocentric, prospective, randomized trial. *J Thorac Dis* 2019; **11**(5): 1788-98.

76. Porcel JM, Cuadrat I, García-Cerecedo T, Pardina M, Bielsa S. Pleural Effusions in Diffuse Large B-Cell Lymphoma: Clinical and Prognostic Significance. *Lung* 2019; **197**(1): 47-51.

77. Tian T, Li J, Hu W, Sun C, Zhou J. Thymidine kinase 1 concentration in pleural effusion is a diagnostic marker and survival predictor for malignant pleural effusion. *J Clin Lab Anal* 2019; **33**(6): e22901.

78. Wang S, Chen H, Zhong J, Qin H, Bai H, Zhao J, et al. Comparative study of EGFR mutations detected in malignant pleural effusion, plasma and tumor tissue in patients with adenocarcinoma of the lung. *Lung Cancer* 2019; **135**: 116-22.

79. Martin GA, Kidd AC, Tsim S, Halford P, Bibby A, Maskell NA, et al. Inter-observer variation in image interpretation and the prognostic importance of non-expansile lung in malignant pleural effusion. *Respirology* 2020; **25**(3): 298-304.

80. Quek JC, Tan QL, Allen JC, Anantham D. Malignant pleural effusion survival prognostication in an Asian population. *Respirology* 2020.

81. Shi XY, Yi FS, Wang Z, Qiao X, Zhai K. Prognostic value of a new score using serum alkaline phosphatase and pleural effusion lactate dehydrogenase for patients with malignant pleural effusion. *Thorac Cancer* 2020; **11**(2): 320-8.

82. Stockhammer P, Ploenes T, Theegarten D, Schuler M, Maier S, Aigner C, et al. Detection of TGF-β in pleural effusions for diagnosis and prognostic stratification of malignant pleural mesothelioma. *Lung Cancer* 2020; **139**: 124-32.

**e Tables**

**eTable 1 Database search strategies**

|  | **Pubmed** | **Cochrane Library** | **Embase** | **Web of Science** | **Medline** |
| --- | --- | --- | --- | --- | --- |
| **1** | PLEURAL EFFUSION | PLEURAL EFFUSION | PLEURAL EFFUSION | pleural effusion | PLEURAL EFFUSION |
| **2** | pleural effusion | pleural effusion | pleural effusion | pleural effusions | pleural effusion |
| **3** | pleural effusions | pleural effusions | pleural effusions | Effusion, Pleural | pleural effusions |
| **4** | Effusion, Pleural | Effusion, Pleural | Effusion, Pleural | Effusions, Pleural | Effusion, Pleural |
| **5** | Effusions, Pleural | Effusions, Pleural | Effusions, Pleural | PE | Effusions, Pleural |
| **6** | PE | PE | PE | 1 or 2 or 3 or 4 or 5 | PE |
| **7** | 1 or 2 or 3 or 4 or 5 or 6 | 1 or 2 or 3 or 4 or 5 or 6 | 1 or 2 or 3 or 4 or 5 or 6 | Neoplasia | 1 or 2 or 3 or 4 or 5 or 6 |
| **8** | NEOPLASIA | NEOPLASIA | NEOPLASIA | Neoplasias | NEOPLASIA |
| **9** | Neoplasia | Neoplasia | Neoplasia | Neoplasm | Neoplasia |
| **10** | Neoplasias | Neoplasias | Neoplasias | Tumors | Neoplasias |
| **11** | Neoplasm | Neoplasm | Neoplasm | Tumor | Neoplasm |
| **12** | Tumors | Tumors | Tumors | Cancer | Tumors |
| **13** | Tumor | Tumor | Tumor | Cancers | Tumor |
| **14** | Cancer | Cancer | Cancer | Malignancy | Cancer |
| **15** | Cancers | Cancers | Cancers | Malignancies | Cancers |
| **16** | Malignancy | Malignancy | Malignancy | Malignant Neoplasms | Malignancy |
| **17** | Malignancies | Malignancies | Malignancies | Malignant Neoplasm | Malignancies |
| **18** | Malignant Neoplasms | Malignant Neoplasms | Malignant Neoplasms | Neoplasm, Malignant | Malignant Neoplasms |
| **19** | Malignant Neoplasm | Malignant Neoplasm | Malignant Neoplasm | Neoplasms, Malignant | Malignant Neoplasm |
| **20** | Neoplasm, Malignant | Neoplasm, Malignant | Neoplasm, Malignant | 7 or 8 or 9 or 10 or 11 or 12 or 13 or 14 or 15 or 16 or 17 or 18 or 19 | Neoplasm, Malignant |
| **21** | Neoplasms, Malignant | Neoplasms, Malignant | Neoplasms, Malignant | Prognosis | Neoplasms, Malignant |
| **22** | 8 or 9 or 10 or 11 or 12 or 13 or 14 or 15 or 16 or 17 or 18 or 19 or 20 or 21 | 8 or 9 or 10 or 11 or 12 or 13 or 14 or 15 or 16 or 17 or 18 or 19 or 20 or 21 | 8 or 9 or 10 or 11 or 12 or 13 or 14 or 15 or 16 or 17 or 18 or 19 or 20 or 21 | Prognoses | 8 or 9 or 10 or 11 or 12 or 13 or 14 or 15 or 16 or 17 or 18 or 19 or 20 or 21 |
| **23** | PROGNOSIS | PROGNOSIS | PROGNOSIS | Prognostic Factor | PROGNOSIS |
| **24** | Prognosis | Prognosis | Prognosis | Prognostic Factors | Prognosis |
| **25** | Prognoses | Prognoses | Prognoses | Factor, Prognostic | Prognoses |
| **26** | Prognostic Factor | Prognostic Factor | Prognostic Factor | Factors, Prognostic | Prognostic Factor |
| **27** | Prognostic Factors | Prognostic Factors | Prognostic Factors | Survival | Prognostic Factors |
| **28** | Factor, Prognostic | Factor, Prognostic | Factor, Prognostic | Outcome | Factor, Prognostic |
| **29** | Factors, Prognostic | Factors, Prognostic | Factors, Prognostic | 21 or 22 or 23 or 24 or 25 or 26 or 27 or 28 | Factors, Prognostic |
| **30** | Survival | Survival | Survival | 6 and 20 and 29 | Survival |
| **31** | Outcome | Outcome | Outcome |  | Outcome |
| **32** | 23 or 24 or 25 or 26 or 27 or 28 or 29 or 30 or 31 | 23 or 24 or 25 or 26 or 27 or 28 or 29 or 30 or 31 | 23 or 24 or 25 or 26 or 27 or 28 or 29 or 30 or 31 |  | 23 or 24 or 25 or 26 or 27 or 28 or 29 or 30 or 31 |
| **33** | 7 and 22 and 32 | 7 and 22 and 32 | 7 and 22 and 32 |  | 7 and 22 and 32 |

Search terms used in combination with the search strategy for retrieving trials developed by The Cochrane Collaboration (JPT 2019); upper case: MeSH or EMTREE heading

Higgins JPT, Thomas J, Chandler J, Cumpston M, Li T, Page MJ, Welch VA (editors). *Cochrane Handbook for Systematic Reviews of Interventions* version 6.0 (updated July 2019). Cochrane, 2019. Available from [www.training.cochrane.org/handbook](http://www.training.cochrane.org/handbook).

**eTable 2 Newcastle-Ottawa Scale (NOS) Score of the Included Studies.**

| Study | Year | NOS scoring | | | | | | | | |
| --- | --- | --- | --- | --- | --- | --- | --- | --- | --- | --- |
|  |  | Representativeness of the Exposed Cohort | Selection of the Non-Exposed Cohort | Ascertainment of Exposure | Demonstration That Outcome of Interest Was Not Present at Start of Study | Comparability of Cohorts on the Basis of the Design or Analysis | Assessment of Outcome | Was Follow-Up Long Enough for Outcomes to Occur | Adequacy of Follow Up of Cohorts | Total score |
| Sahn et al | 1988 | 1 | 1 | 1 | 0 | 2 | 1 | 1 | 1 | 8 |
| Panadero et al | 1989 | 1 | 1 | 1 | 1 | 2 | 1 | 1 | 1 | 9 |
| Foresti et al | 1990 | 1 | 1 | 1 | 0 | 1 | 1 | 1 | 1 | 7 |
| Gottehrer et al | 1991 | 1 | 1 | 1 | 0 | 1 | 1 | 0 | 1 | 6 |
| Sugiura et al | 1997 | 1 | 1 | 1 | 0 | 2 | 1 | 1 | 1 | 8 |
| Moragón et al | 1998 | 1 | 1 | 1 | 0 | 2 | 1 | 0 | 1 | 7 |
| Burrows et al | 2000 | 1 | 1 | 1 | 0 | 2 | 1 | 1 | 1 | 8 |
| Heffner et al | 2000 | 1 | 1 | 1 | 0 | 2 | 1 | 1 | 1 | 8 |
| Chen et al | 2001 | 1 | 1 | 1 | 0 | 2 | 1 | 1 | 1 | 8 |
| Thyle´n et al | 2001 | 1 | 1 | 1 | 0 | 2 | 1 | 1 | 1 | 8 |
| Bernard et al | 2002 | 1 | 1 | 1 | 0 | 2 | 1 | 1 | 1 | 8 |
| Eitan et al | 2005 | 1 | 1 | 1 | 1 | 2 | 1 | 1 | 1 | 9 |
| Aelony et al | 2006 | 1 | 1 | 1 | 0 | 1 | 1 | 1 | 1 | 7 |
| Aoe et al | 2006 | 1 | 1 | 1 | 0 | 2 | 1 | 1 | 1 | 8 |
| Soh et al | 2006 | 1 | 1 | 1 | 0 | 2 | 1 | 0 | 1 | 7 |
| Bielsa et al | 2008 | 1 | 1 | 1 | 0 | 2 | 1 | 1 | 1 | 8 |
| Wu et al | 2008 | 1 | 1 | 1 | 0 | 2 | 1 | 1 | 1 | 8 |
| Hsu et al | 2009 | 1 | 1 | 1 | 0 | 2 | 1 | 1 | 1 | 8 |
| Wu et al | 2009 | 1 | 1 | 1 | 0 | 2 | 1 | 1 | 1 | 8 |
| Kotyza et al | 2010 | 1 | 1 | 1 | 0 | 2 | 1 | 1 | 1 | 8 |
| Lan et al | 2010 | 1 | 1 | 1 | 1 | 1 | 1 | 1 | 1 | 8 |
| Ozyurtkan et al | 2010 | 1 | 1 | 1 | 0 | 2 | 1 | 1 | 1 | 8 |
| Pilling et al | 2010 | 1 | 1 | 1 | 0 | 2 | 1 | 1 | 1 | 8 |
| Tanrikulu et al | 2010 | 1 | 1 | 1 | 0 | 2 | 1 | 1 | 1 | 8 |
| Hirayama et al | 2010 | 1 | 1 | 1 | 0 | 2 | 1 | 0 | 1 | 7 |
| Park et al | 2011 | 1 | 1 | 1 | 0 | 2 | 1 | 1 | 1 | 8 |
| Sakr et al | 2011 | 1 | 1 | 1 | 0 | 2 | 1 | 1 | 1 | 8 |
| Yamada et al | 2011 | 1 | 1 | 1 | 0 | 1 | 1 | 1 | 1 | 7 |
| Guo et al | 2011 | 1 | 1 | 1 | 0 | 2 | 1 | 1 | 1 | 8 |
| Hooper et al | 2012 | 1 | 1 | 1 | 0 | 2 | 1 | 0 | 1 | 7 |
| Maribel et al | 2012 | 1 | 1 | 1 | 1 | 1 | 1 | 1 | 1 | 8 |
| Qian et al | 2012 | 1 | 1 | 1 | 0 | 2 | 1 | 0 | 1 | 7 |
| Qian et al | 2012 | 1 | 1 | 1 | 1 | 2 | 1 | 0 | 1 | 8 |
| Wang et al | 2012 | 1 | 1 | 1 | 0 | 2 | 1 | 1 | 1 | 8 |
| Cheng et al | 2013 | 1 | 1 | 1 | 1 | 2 | 1 | 1 | 1 | 9 |
| Faiz et al | 2013 | 1 | 1 | 1 | 0 | 2 | 1 | 1 | 1 | 8 |
| Gorgun et al | 2013 | 1 | 1 | 1 | 0 | 2 | 1 | 1 | 1 | 8 |
| Park et al | 2013 | 1 | 1 | 1 | 0 | 2 | 1 | 1 | 1 | 8 |
| Wu et al | 2013 | 1 | 1 | 1 | 1 | 2 | 1 | 1 | 1 | 9 |
| Anevlavis et al | 2014 | 1 | 1 | 1 | 0 | 2 | 1 | 1 | 1 | 8 |
| Clive et al | 2014 | 1 | 1 | 1 | 1 | 2 | 1 | 1 | 1 | 9 |
| Ni et al | 2014 | 1 | 1 | 1 | 0 | 2 | 1 | 1 | 1 | 8 |
| Xu et al | 2014 | 1 | 1 | 1 | 0 | 2 | 1 | 0 | 1 | 7 |
| Zhang et al | 2014 | 1 | 1 | 1 | 0 | 2 | 1 | 1 | 1 | 8 |
| Zhang et al | 2014 | 1 | 1 | 1 | 0 | 2 | 1 | 1 | 1 | 8 |
| Abrao et al | 2015 | 1 | 1 | 1 | 0 | 2 | 1 | 0 | 1 | 7 |
| Gkiozos et al | 2015 | 1 | 1 | 1 | 1 | 1 | 1 | 1 | 1 | 8 |
| Porcel et al | 2015 | 1 | 1 | 1 | 0 | 2 | 1 | 1 | 1 | 8 |
| Xu et al | 2015 | 1 | 1 | 1 | 0 | 2 | 1 | 1 | 1 | 8 |
| Zamboni et al | 2015 | 1 | 1 | 1 | 0 | 2 | 1 | 1 | 1 | 8 |
| Zhao et al | 2015 | 1 | 1 | 1 | 0 | 1 | 1 | 1 | 1 | 7 |
| Abrao et al | 2016 | 1 | 1 | 1 | 1 | 2 | 1 | 0 | 1 | 8 |
| Hsu et al | 2016 | 1 | 1 | 1 | 1 | 2 | 1 | 1 | 1 | 9 |
| Kasapoglu et al | 2016 | 1 | 1 | 1 | 0 | 2 | 1 | 1 | 1 | 8 |
| Psallidas et al | 2016 | 1 | 1 | 1 | 0 | 2 | 1 | 1 | 1 | 8 |
| Tamiya, et al | 2016 | 1 | 1 | 1 | 0 | 1 | 1 | 1 | 1 | 7 |
| Terra et al | 2016 | 1 | 1 | 1 | 0 | 2 | 1 | 1 | 1 | 8 |
| Usui et al | 2016 | 1 | 1 | 1 | 0 | 1 | 1 | 1 | 1 | 7 |
| Verma et al | 2016 | 1 | 1 | 1 | 0 | 2 | 1 | 1 | 1 | 8 |
| Yang et al | 2016 | 1 | 1 | 1 | 0 | 2 | 1 | 1 | 1 | 8 |
| Amn et al | 2017 | 1 | 1 | 1 | 0 | 2 | 1 | 1 | 1 | 8 |
| Lee et al | 2017 | 1 | 1 | 1 | 0 | 2 | 1 | 1 | 1 | 8 |
| Lu et al | 2017 | 1 | 1 | 1 | 0 | 2 | 1 | 1 | 1 | 8 |
| Yang et al | 2017 | 1 | 1 | 1 | 0 | 1 | 1 | 1 | 1 | 7 |
| Zheng et al | 2017 | 1 | 1 | 1 | 0 | 2 | 1 | 1 | 1 | 8 |
| Abisheganaden et al | 2018 | 1 | 1 | 1 | 0 | 2 | 1 | 0 | 1 | 7 |
| Elena et al | 2018 | 1 | 1 | 1 | 0 | 2 | 1 | 0 | 1 | 7 |
| Han et al | 2018 | 1 | 1 | 1 | 0 | 2 | 1 | 1 | 1 | 8 |
| [Jeba et al](https://www.ncbi.nlm.nih.gov/pubmed/?term=Jeba%20J%5BAuthor%5D&cauthor=true&cauthor_uid=29736123) | 2018 | 1 | 1 | 1 | 0 | 2 | 1 | 1 | 1 | 8 |
| Lim et al | 2018 | 1 | 1 | 1 | 0 | 2 | 1 | 1 | 1 | 8 |
| Porcel et al | 2018 | 1 | 1 | 1 | 0 | 1 | 1 | 1 | 1 | 7 |
| Psallidas et al | 2018 | 1 | 1 | 1 | 1 | 2 | 1 | 1 | 1 | 9 |
| Wu et al | 2018 | 1 | 1 | 1 | 0 | 2 | 1 | 1 | 1 | 8 |
| Xu et al | 2018 | 1 | 1 | 1 | 0 | 1 | 1 | 1 | 1 | 7 |
| Foote et al | 2019 | 1 | 1 | 1 | 0 | 2 | 1 | 1 | 1 | 8 |
| Kleontas et al | 2019 | 1 | 1 | 1 | 1 | 2 | 1 | 1 | 1 | 9 |
| Tian et al | 2019 | 1 | 1 | 1 | 0 | 2 | 1 | 1 | 1 | 8 |
| Wang et al | 2019 | 1 | 1 | 1 | 0 | 2 | 1 | 1 | 1 | 8 |
| Martin et al | 2020 | 1 | 1 | 1 | 0 | 2 | 1 | 1 | 1 | 8 |
| Quek et al | 2020 | 1 | 1 | 1 | 0 | 2 | 1 | 0 | 1 | 7 |
| Shi et al | 2020 | 1 | 1 | 1 | 0 | 2 | 1 | 0 | 1 | 7 |
| Stockhammer et al | 2020 | 1 | 1 | 1 | 0 | 2 | 1 | 1 | 1 | 8 |

**eFigures**

**eFigure 1. Forest plot of the hazard ratios of age for overall survival in malignant patients with pleural effusion.**


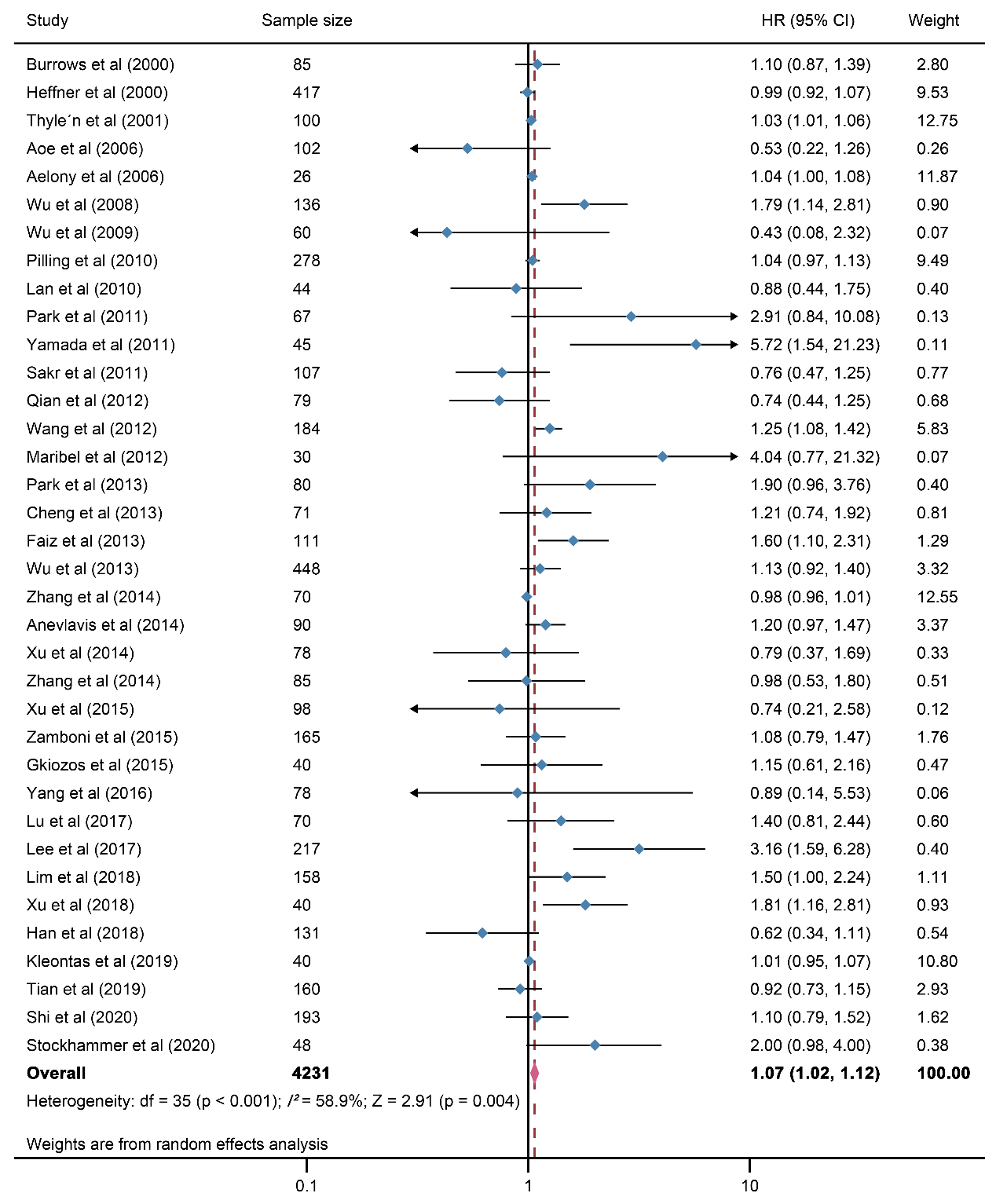


**eFigure 2. Forest plot of the hazard ratios of gender for overall survival in malignant patients with pleural effusion.**


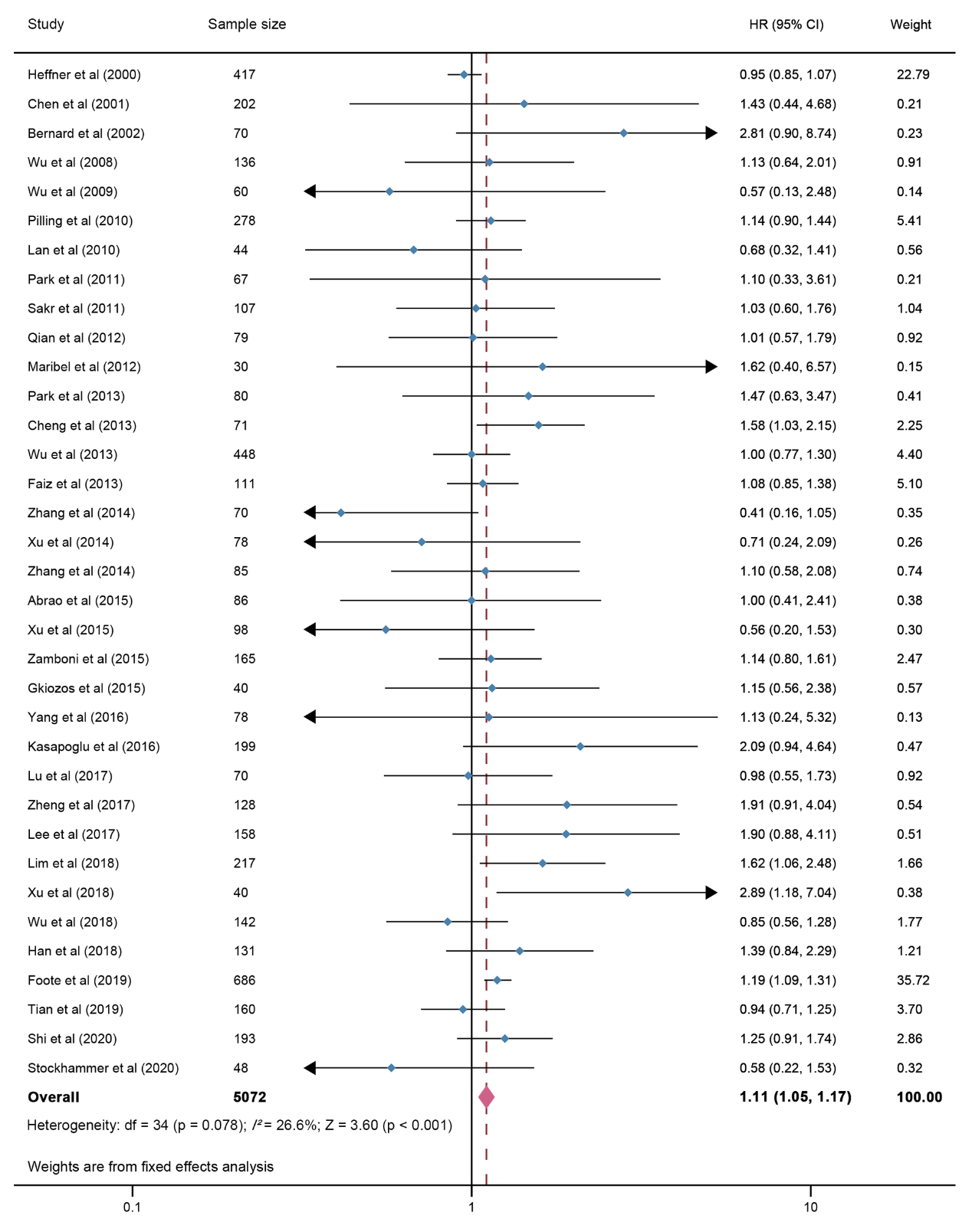


**eFigure 3. Forest plot of the hazard ratios of smoking status for overall survival in malignant patients with pleural effusion.**


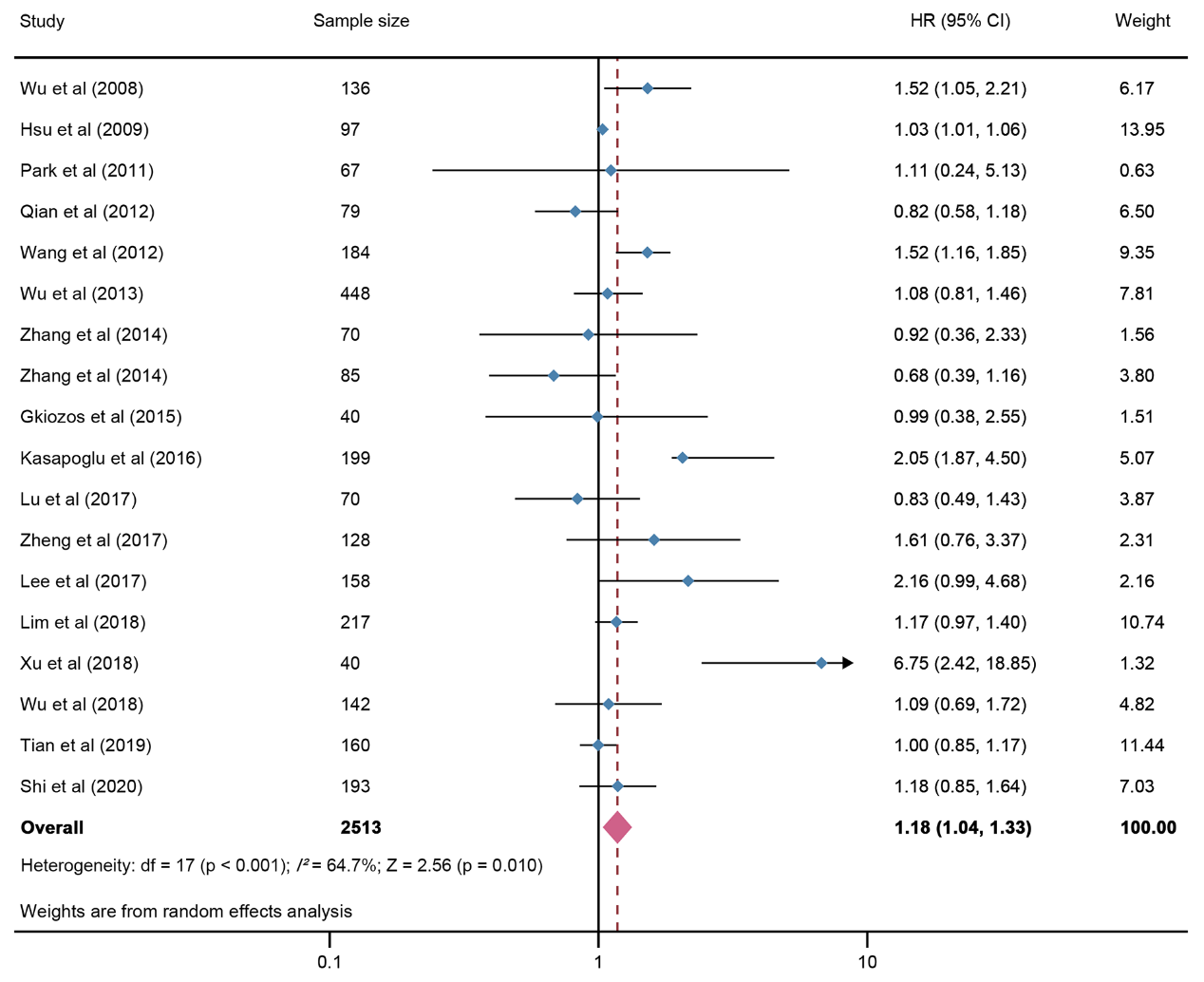


**eFigure 4. Forest plot of the hazard ratios of ECOG PS for overall survival in malignant patients with pleural effusion.**


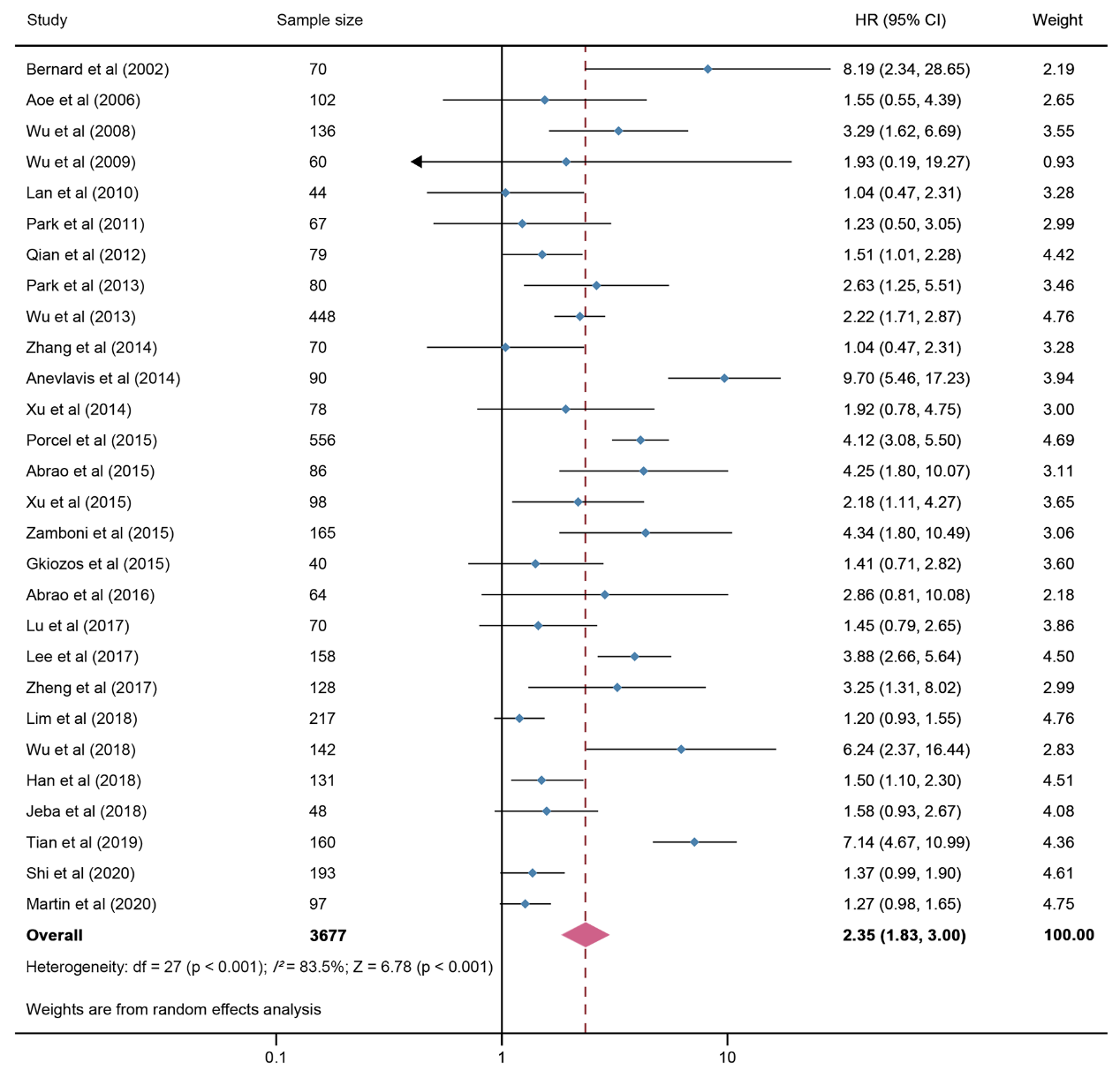


**eFigure 5. Forest plot of the hazard ratios of stage for overall survival in malignant patients with pleural effusion.**


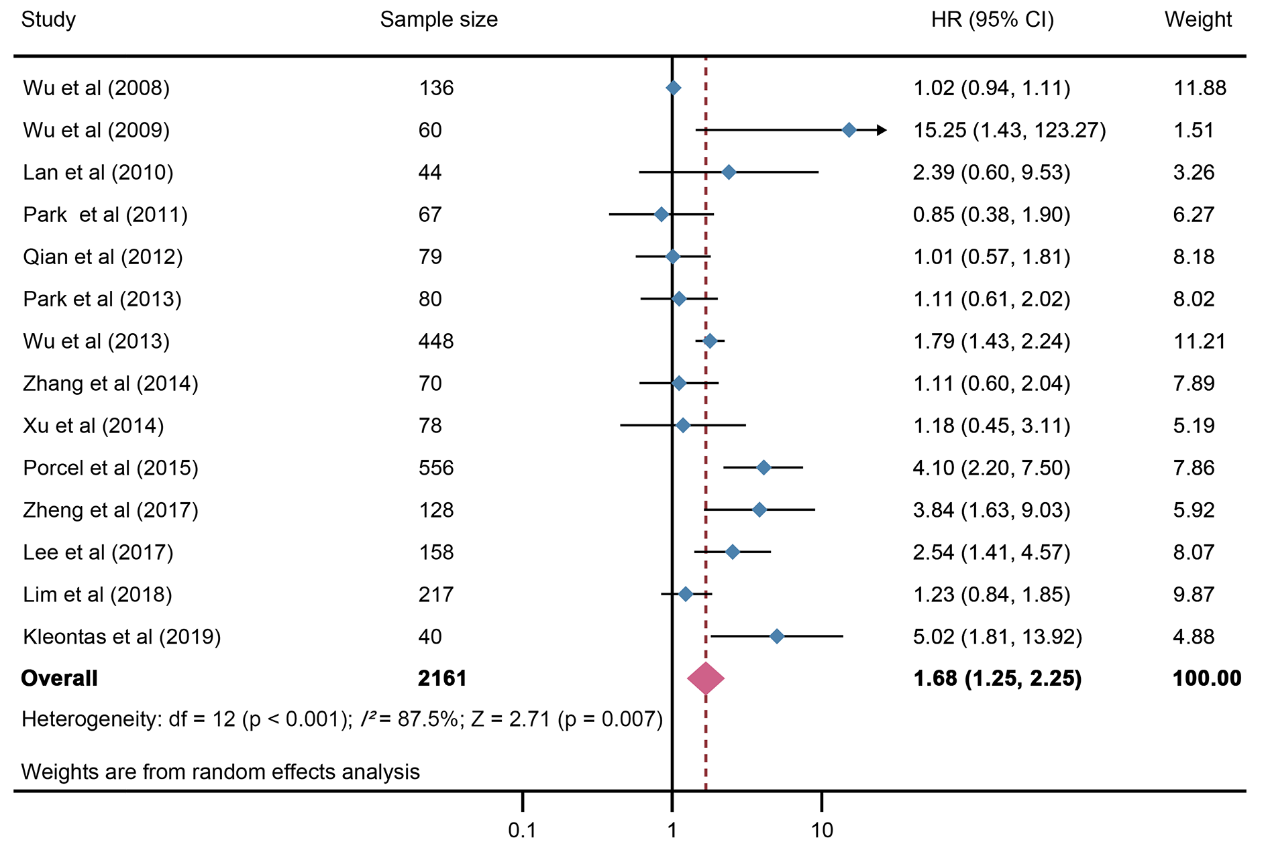


**eFigure 6. Forest plot of the hazard ratios of histology for overall survival in malignant patients with pleural effusion.**


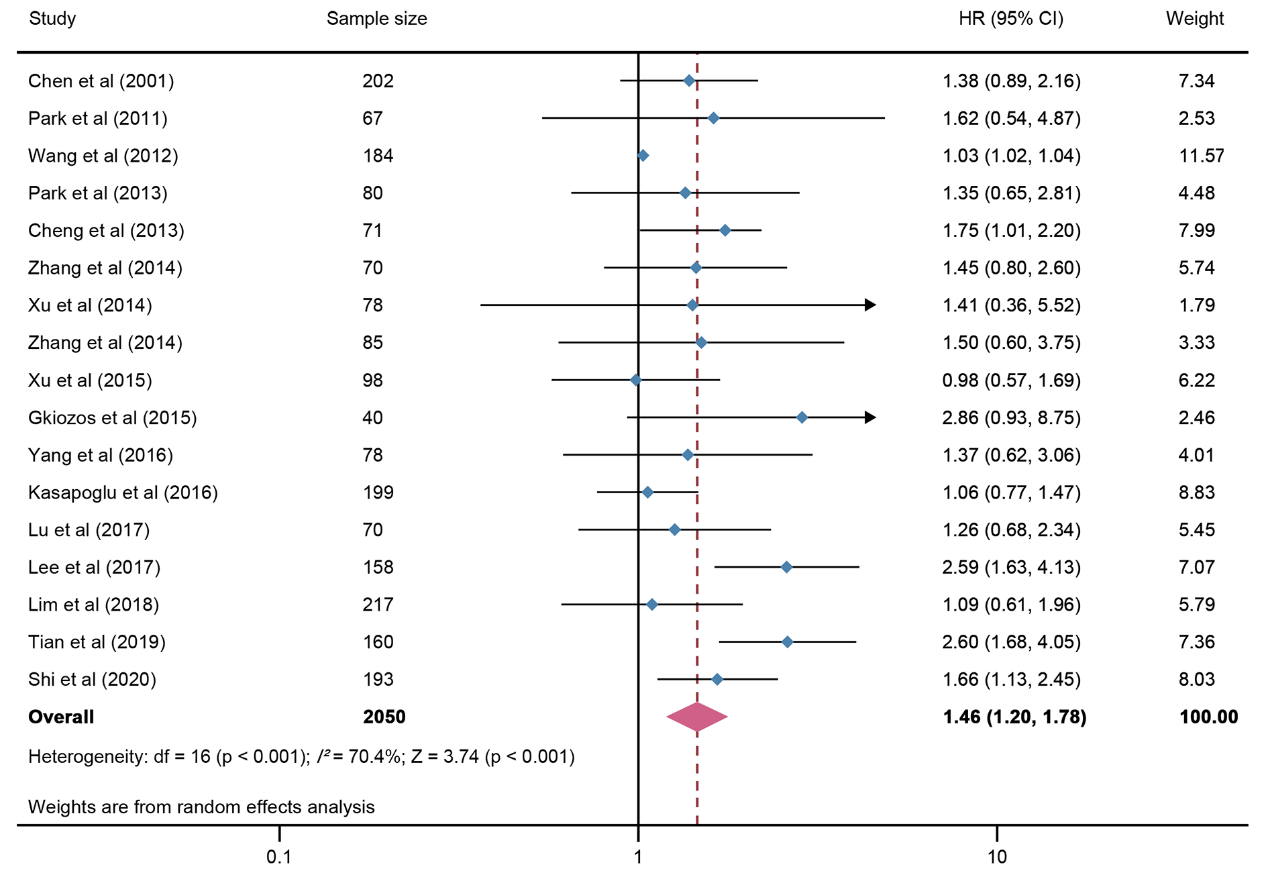


**eFigure 7. Forest plot of the hazard ratios of cytology for overall survival in malignant patients with pleural effusion.**


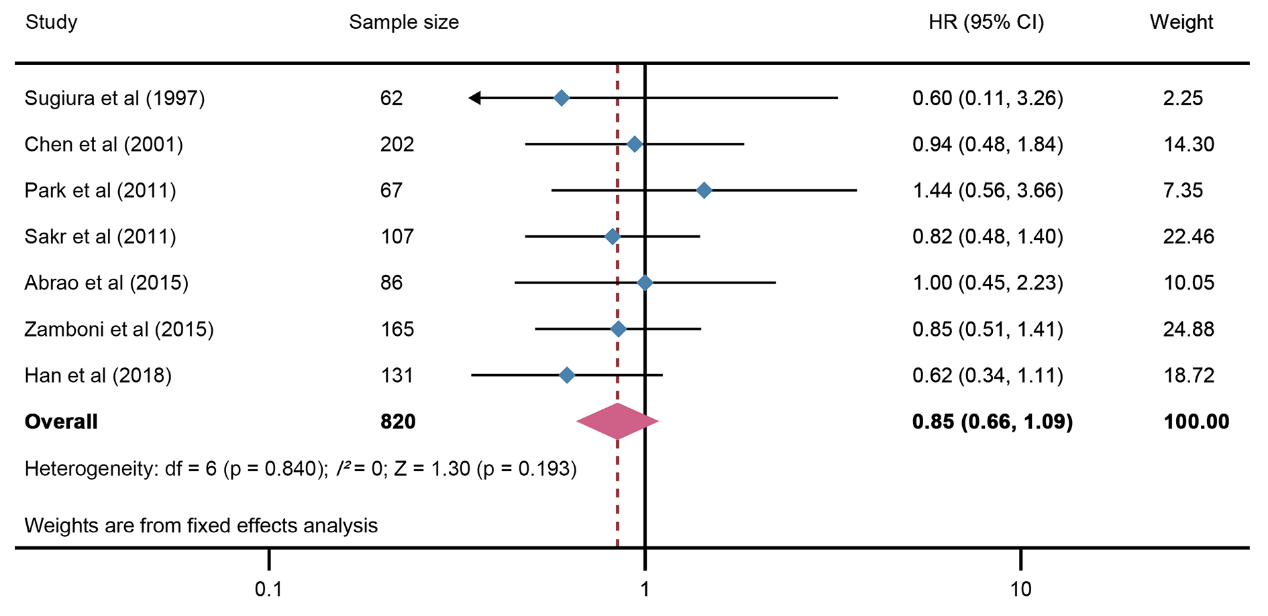


**eFigure 8. Forest plot of the hazard ratios of distant metastasis for overall survival in malignant patients with pleural effusion.**


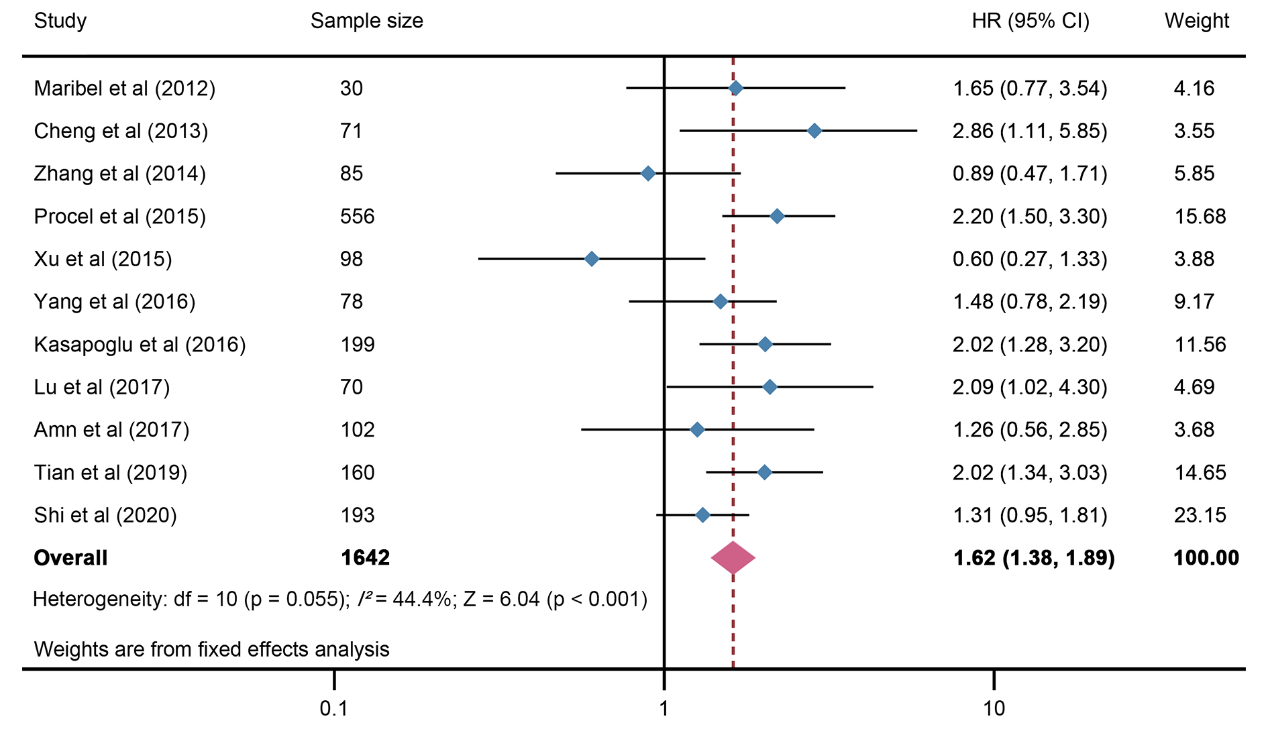


**eFigure 9. Forest plot of the hazard ratios of EGFR mutation for overall survival in malignant patients with pleural effusion.**


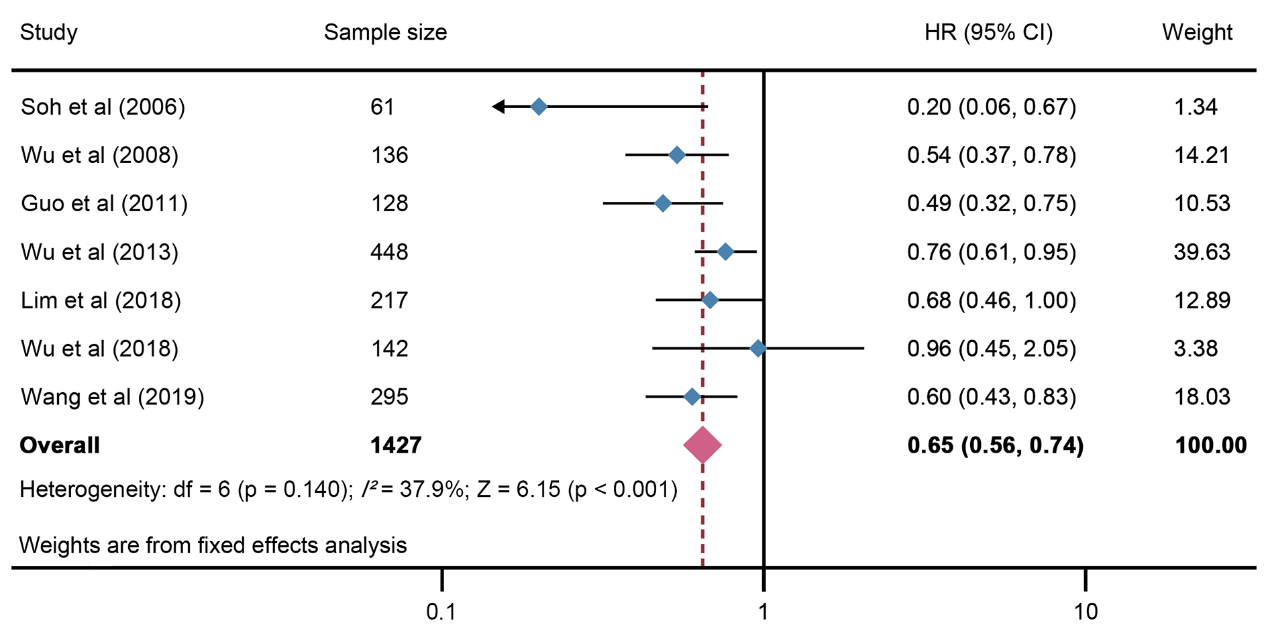


**eFigure 10. Forest plot of the hazard ratios of LENT score for overall survival in malignant patients with pleural effusion.**


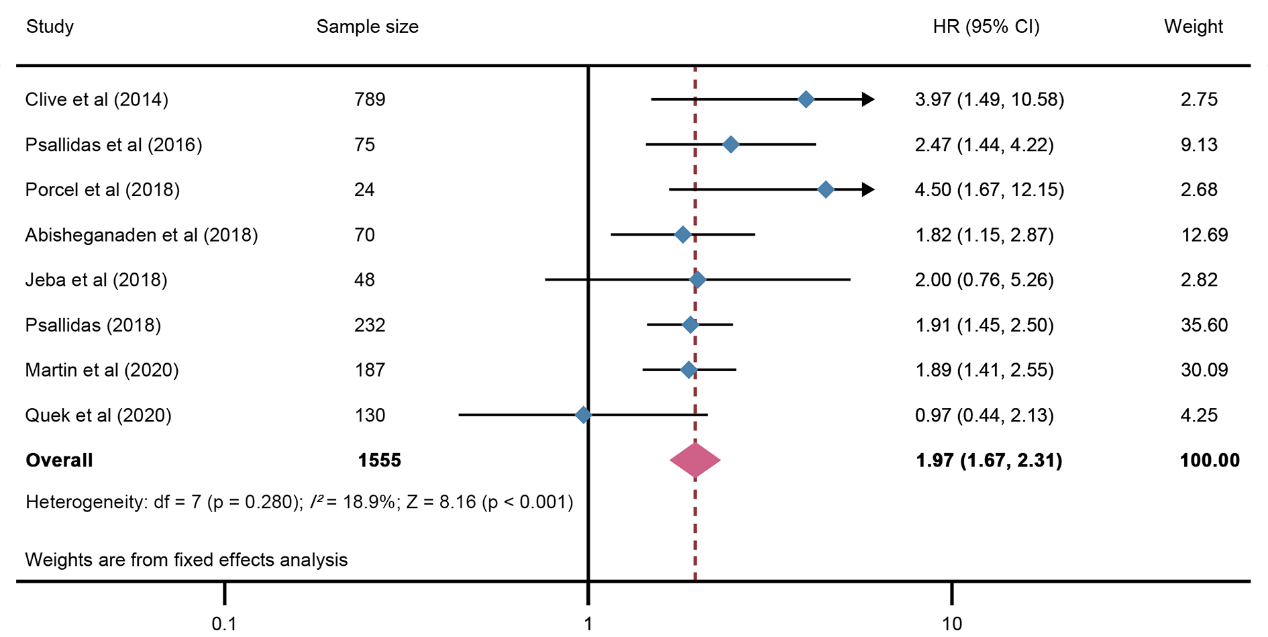


**eFigure 11. Forest plot of the hazard ratios of age for progression-free survival in malignant patients with pleural effusion.**

**
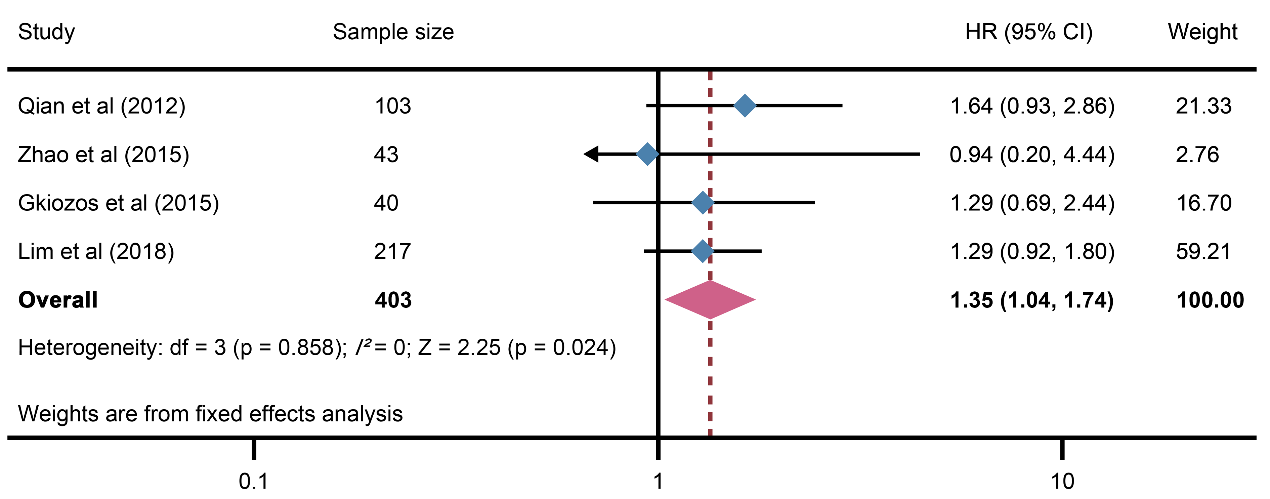
**

**eFigure 12. Forest plot of the hazard ratios of gender for progression-free survival in malignant patients with pleural effusion.**


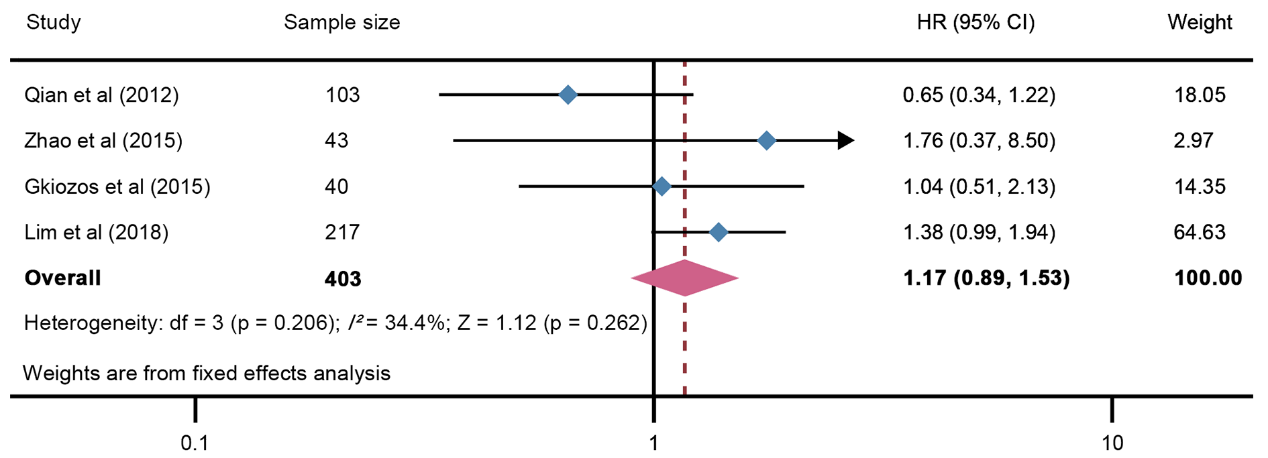


**eFigure 13. Forest plot of the hazard ratios of smoking status for progression-free survival in malignant patients with pleural effusion.**


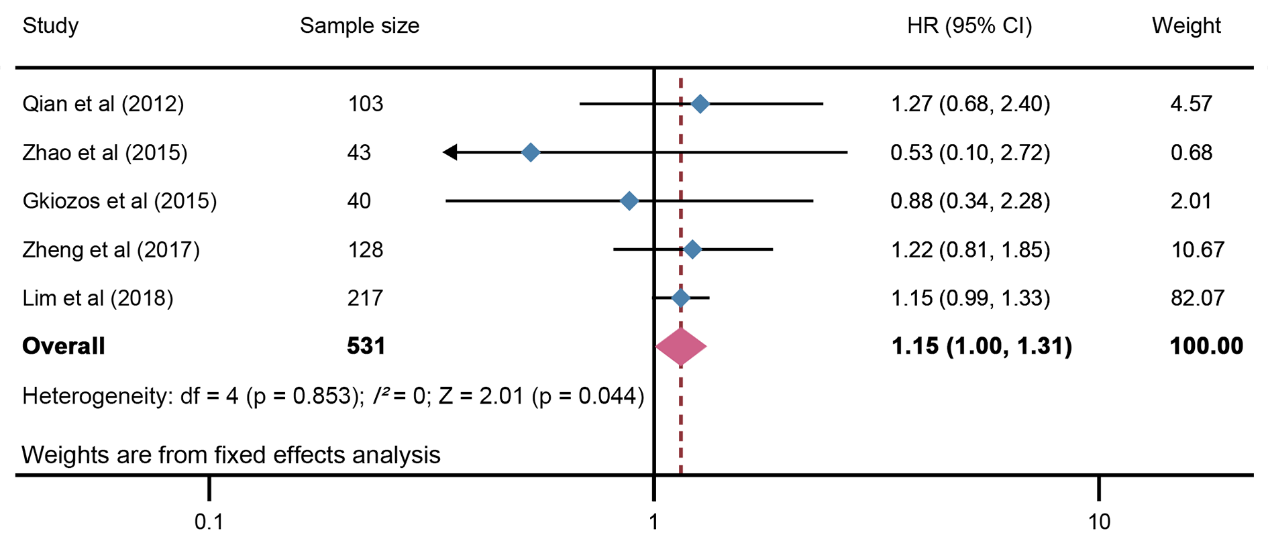


**eFigure 14. Forest plot of the hazard ratios of ECOG PS for progression-free survival in malignant patients with pleural effusion.**


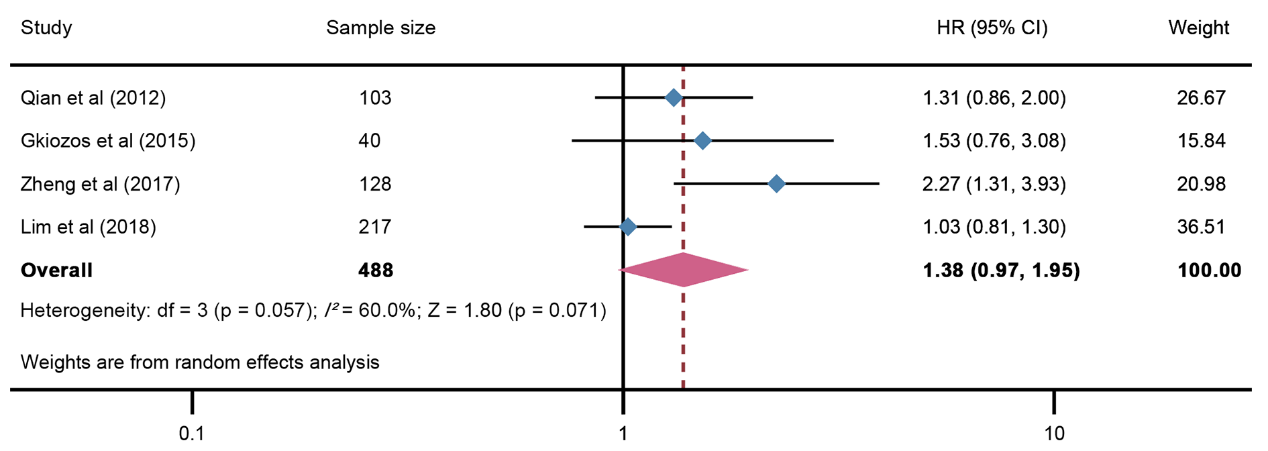


**eFigure 15. Forest plot of the hazard ratios of stage fo****r progression-free survival in malignant patients with pleural effusion.**


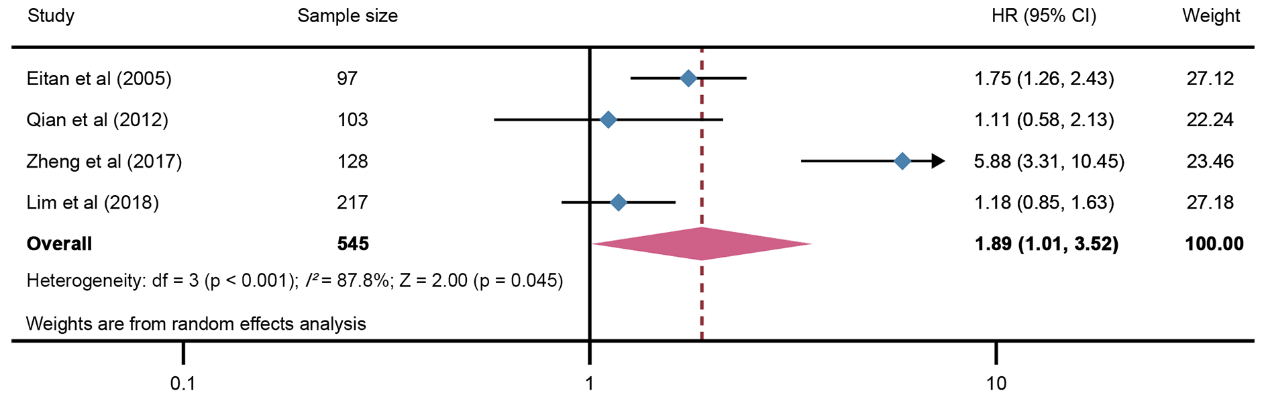


**eFigure 16. Forest plot of the hazard ratios of EGFR mutation for progression-free survival in malignant patients with pleural effusion.**


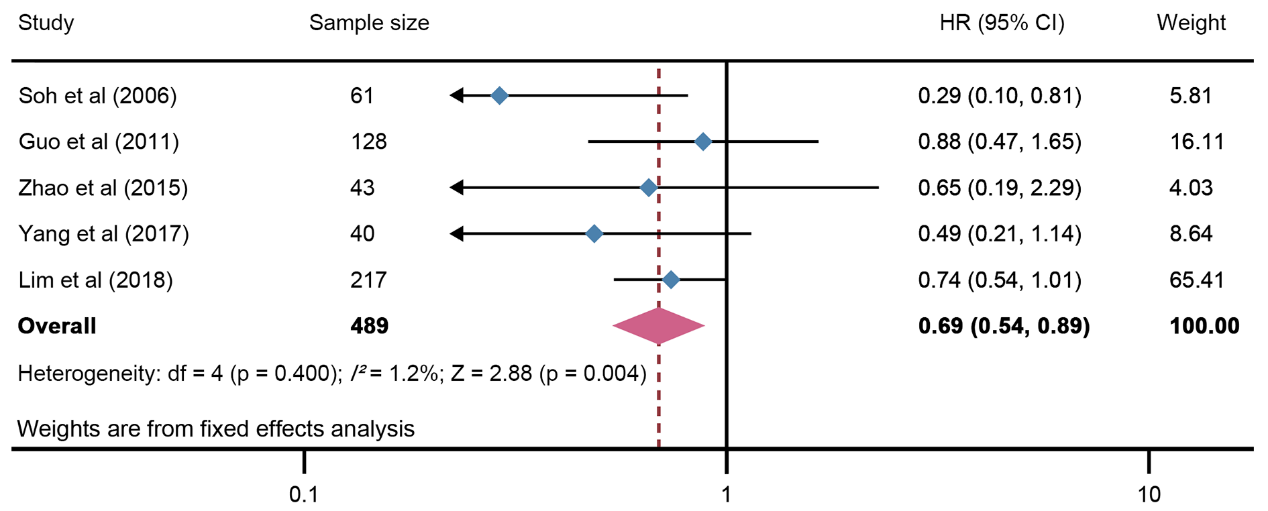


**eFigure 17. Forest plot of the hazard ratios of serum WBC for overall survival in malignant patients with pleural effusion.**


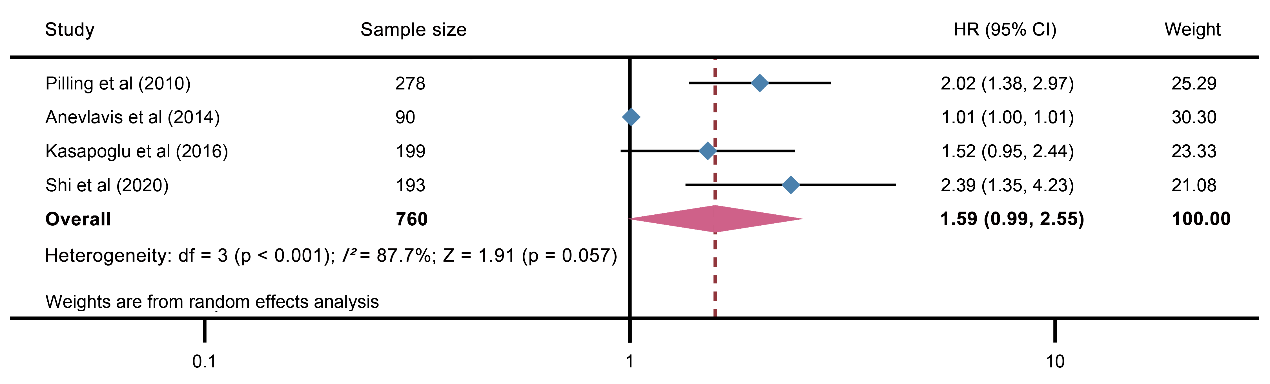


**eFigure 18. Forest plot of the hazard ratios of serum NLR for overall survival in malignant patients with pleural effusion.**


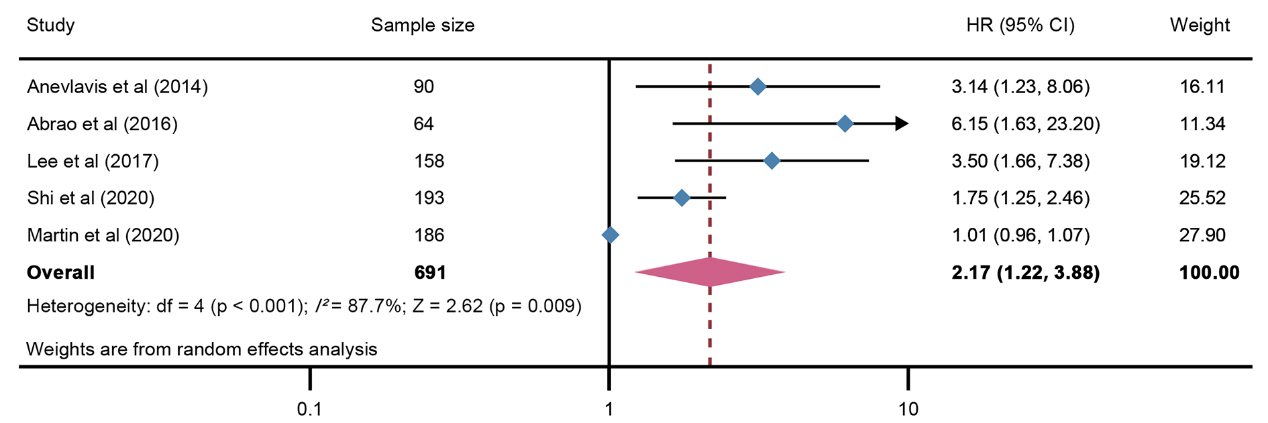


**eFigure 19. Forest plot of the hazard ratios of serum hemoglobin for overall survival in malignant patients with pleural effusion.**


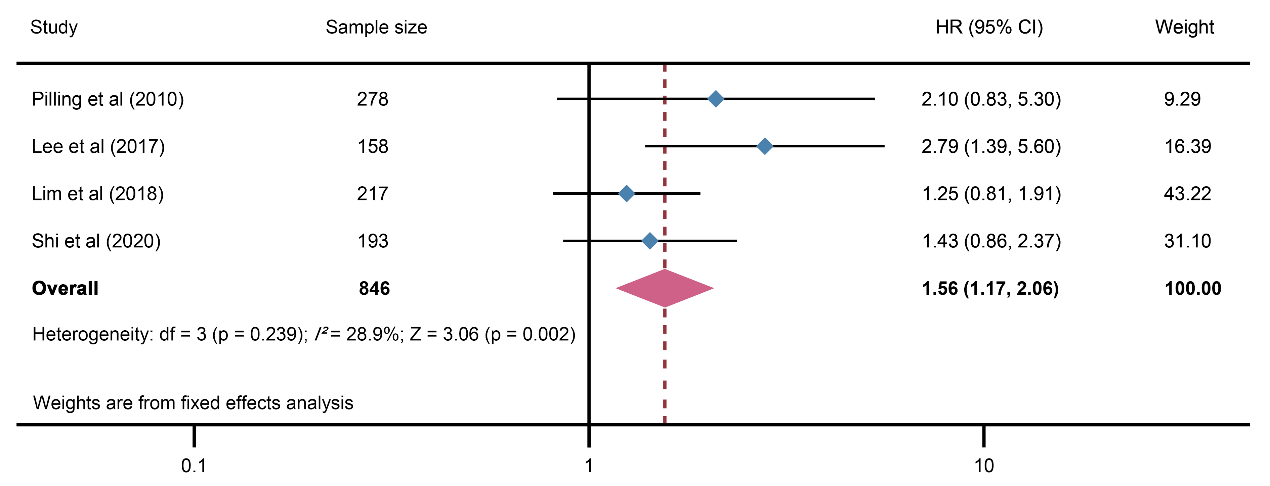


**eFigure 20. Forest plot of the hazard ratios of serum total protein for overall survival in malignant patients with pleural effusion.**


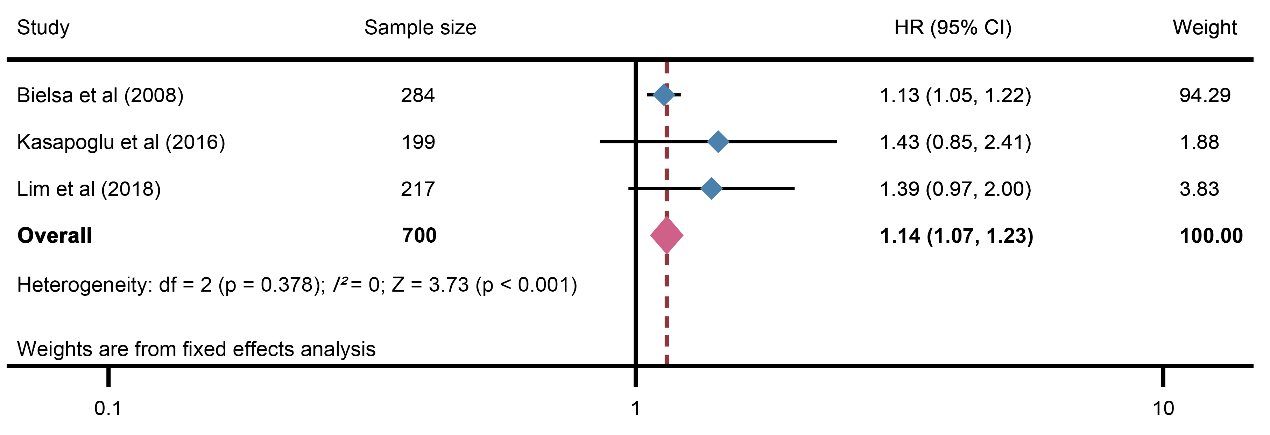


**eFigure 21. Forest plot of the hazard ratios of serum albumin for overall survival in malignant patients with pleural effusion.**


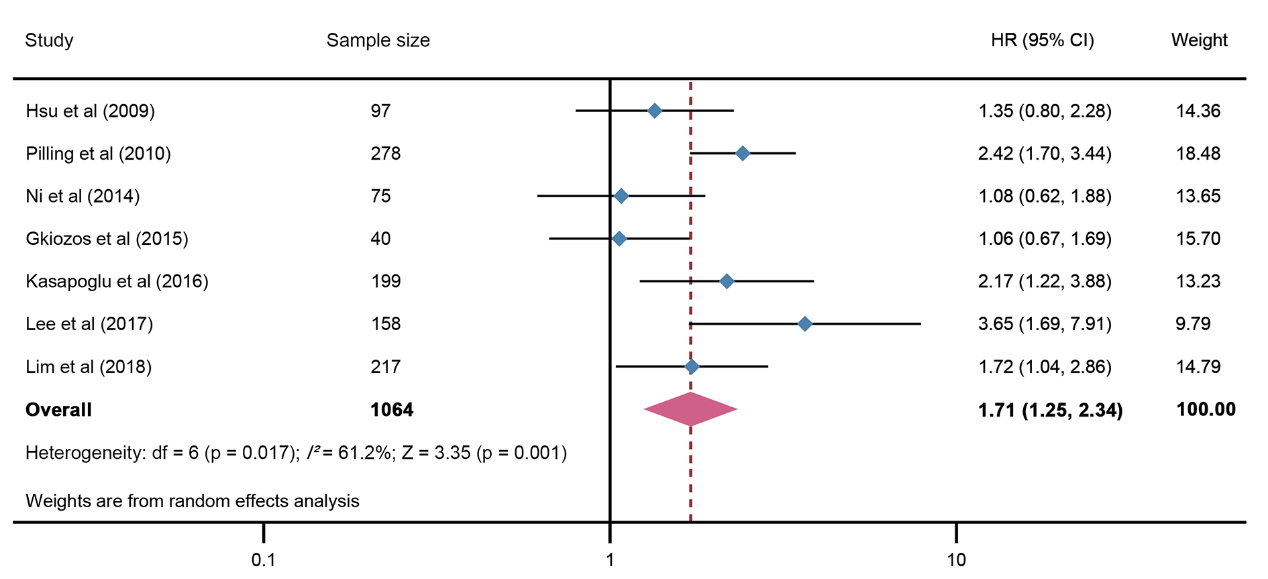


**eFigure 22. Forest plot of the hazard ratios of serum LDH for overall survival in malignant patients with pleural effusion.**


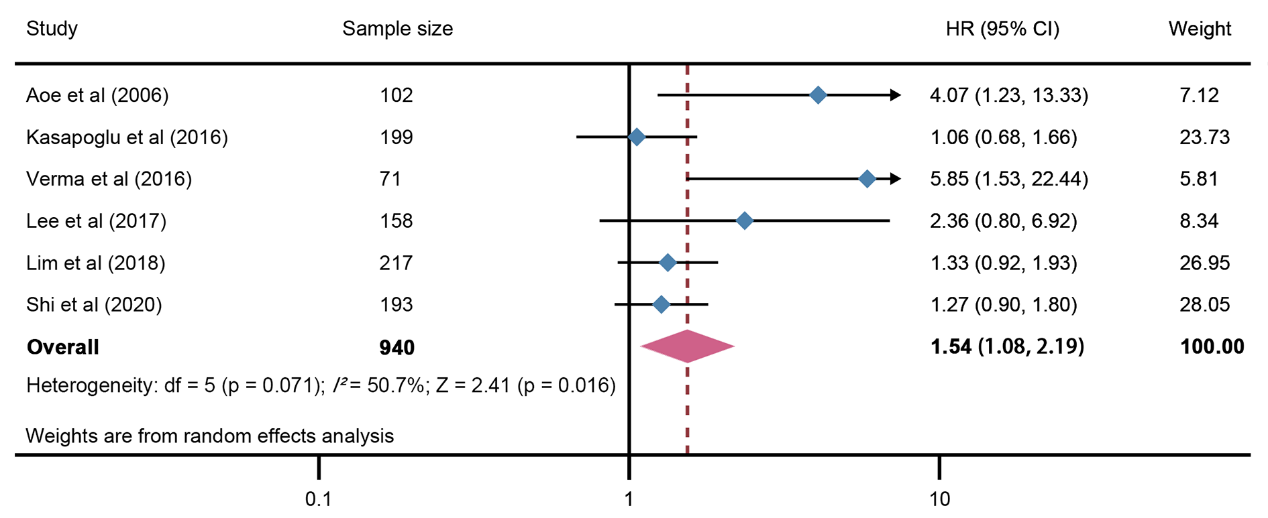


**eFigure 23. Forest plot of the hazard ratios of serum CRP for overall survival in malignant patients with pleural effusion.**


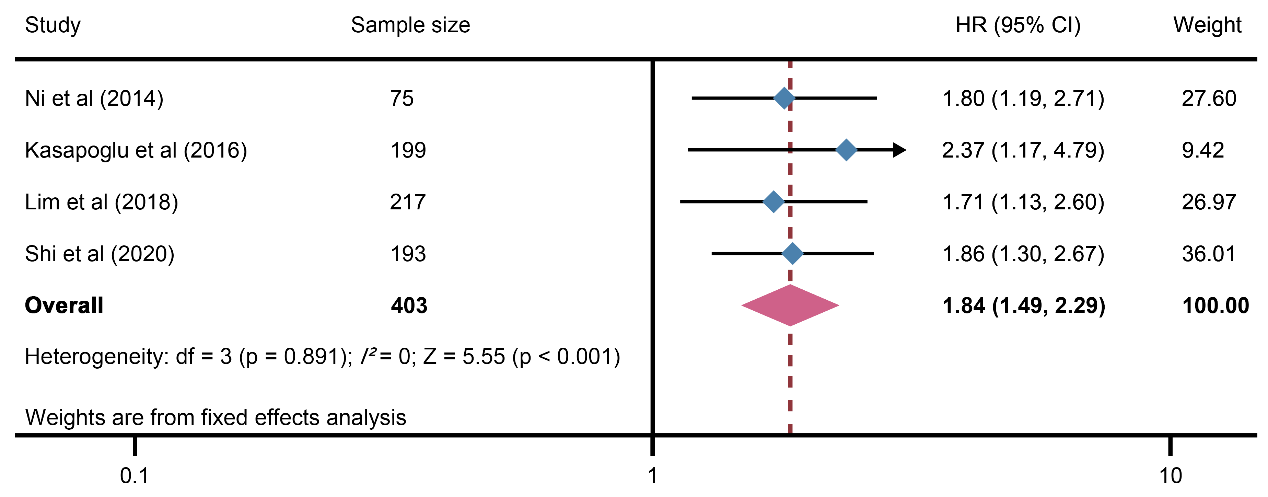


**eFigure 24. Forest plot of the hazard ratios of serum VEGF for overall survival in malignant patients with pleural effusion.**


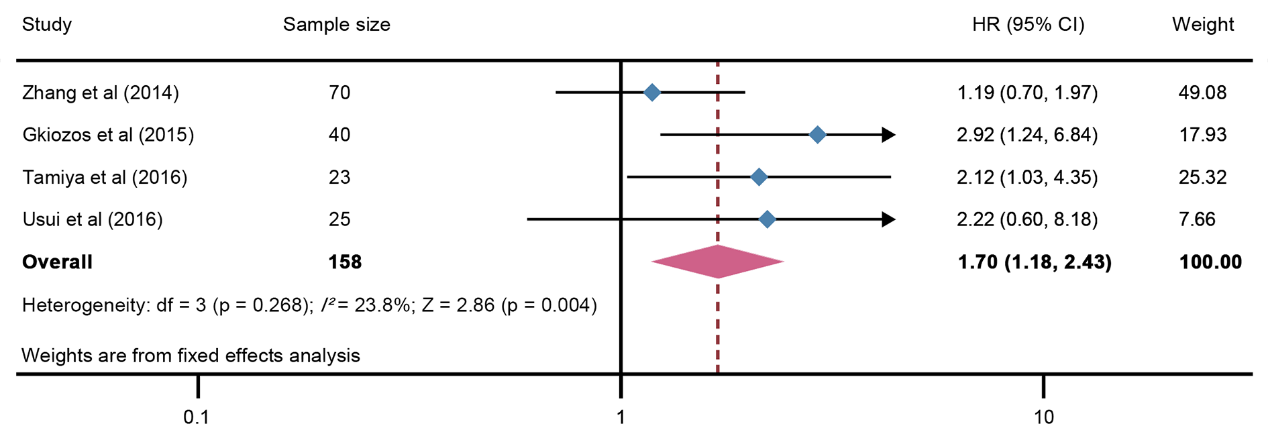


**eFigure 25. Forest plot of the hazard ratios of serum VEGF for progression-free survival in malignant patients with pleural effusion.**


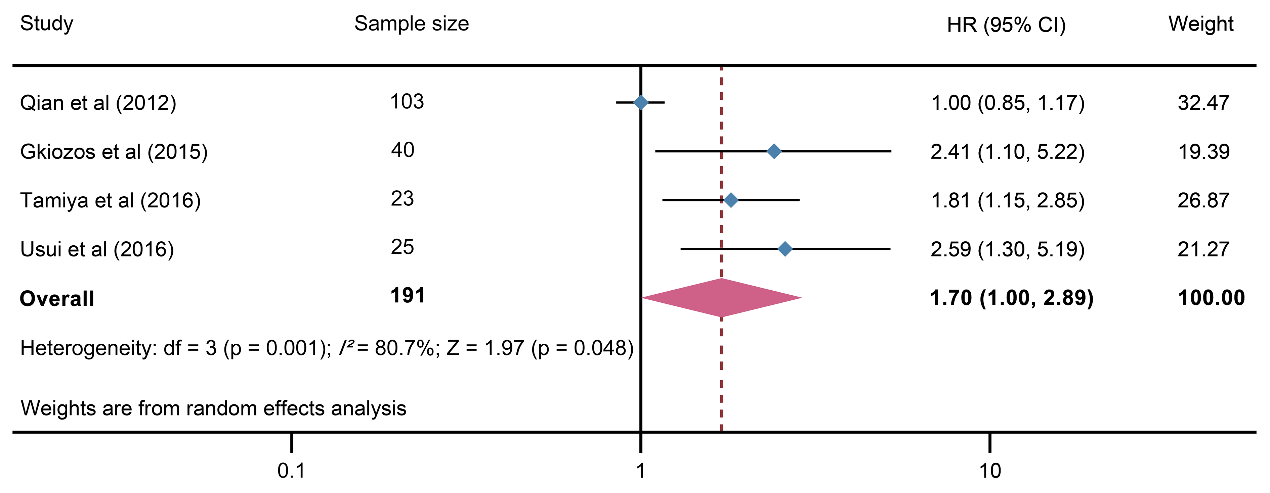


**eFigure 26. Forest plot of the hazard ratios of pleural effusion neutrophils for overall survival in malignant patients.**

**
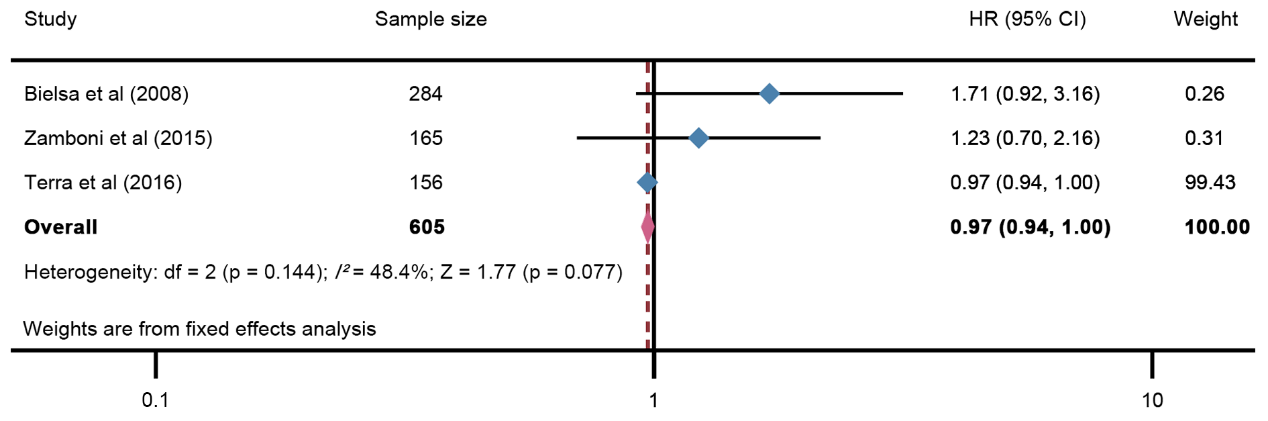
**

**eFigure 27. Forest plot of the hazard ratios of pleural effusion PH for overall survival in malignant patients.**

**
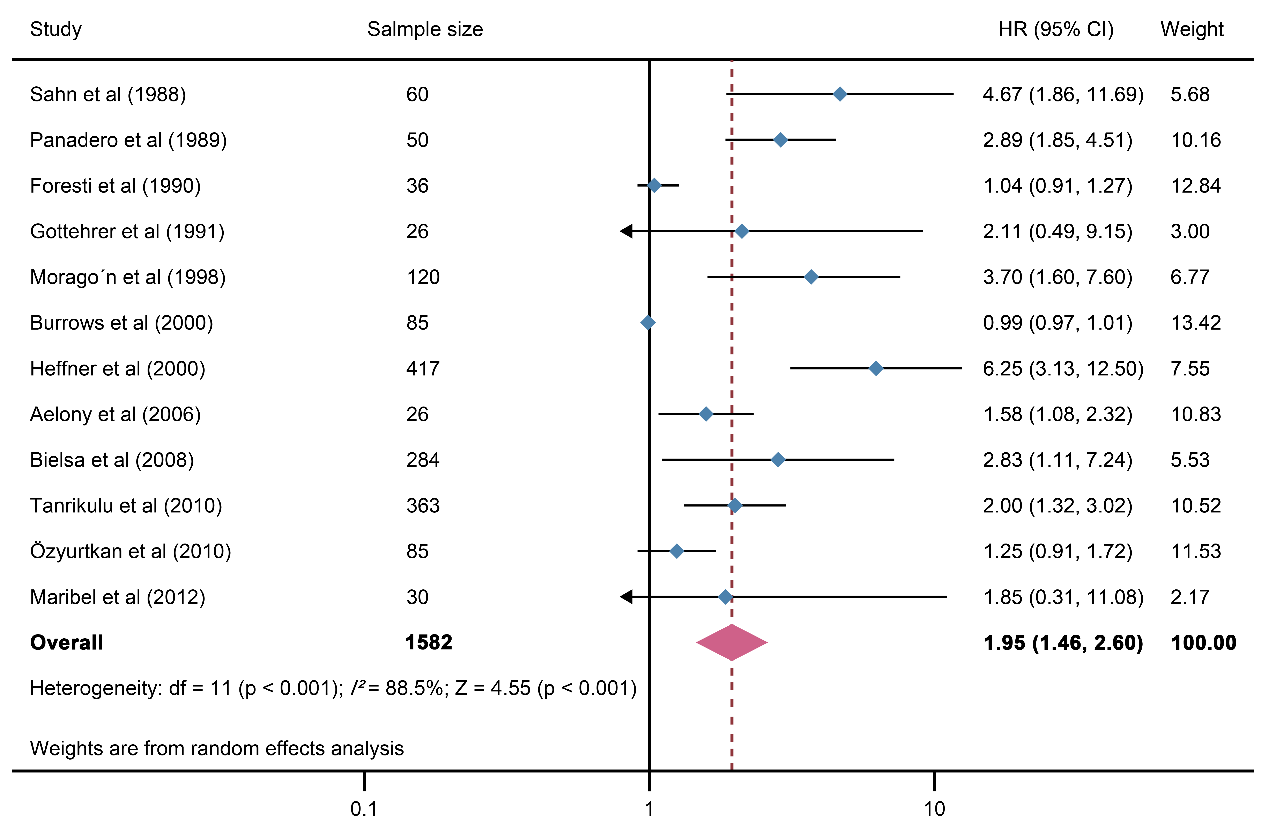
**

**eFigure 28. Forest plot of the hazard ratios of pleural effusion total protein for overall survival in malignant patients.**


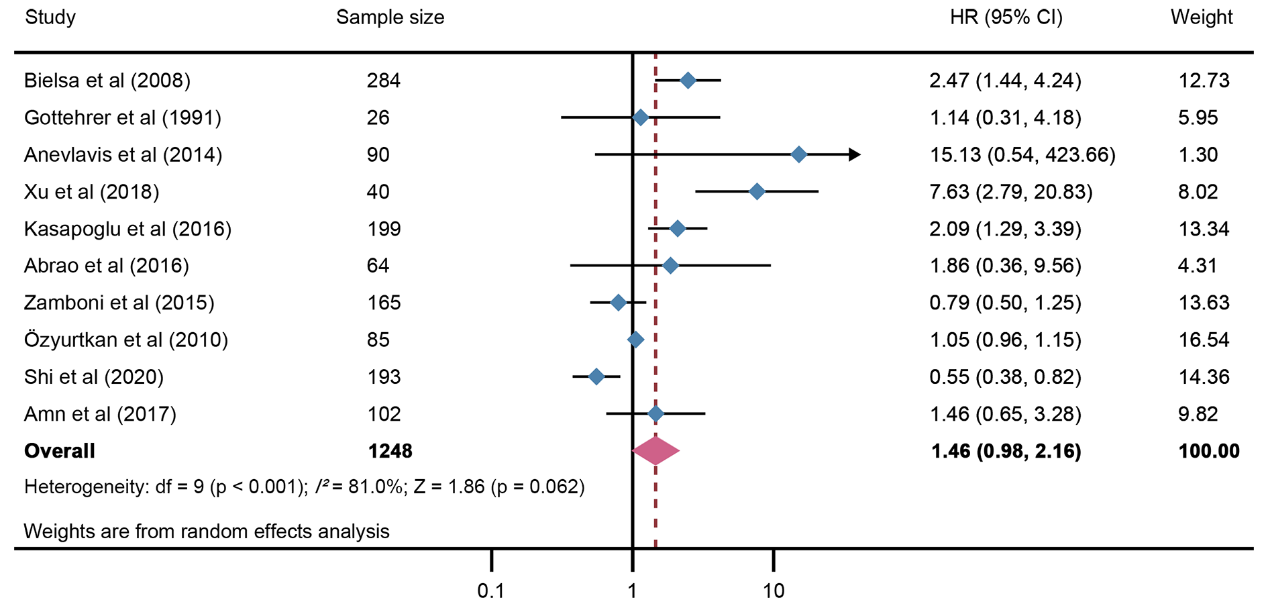


**eFigure 29. Forest plot of the hazard ratios of pleural effusion albumin for overall survival in malignant patients.**


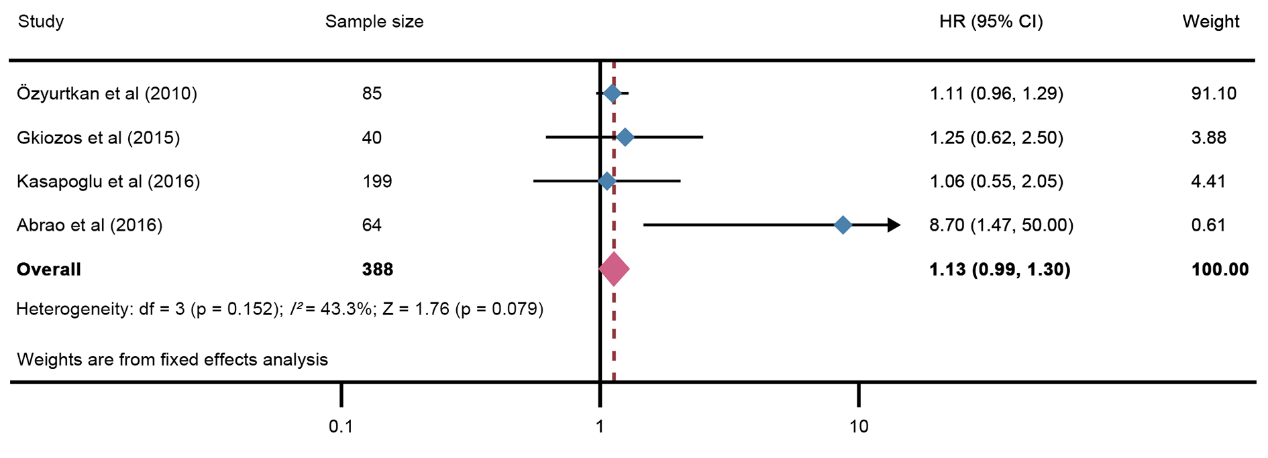


**eFigure 30. Forest plot of the hazard ratios of pleural effusion glucose for overall survival in malignant patients.**


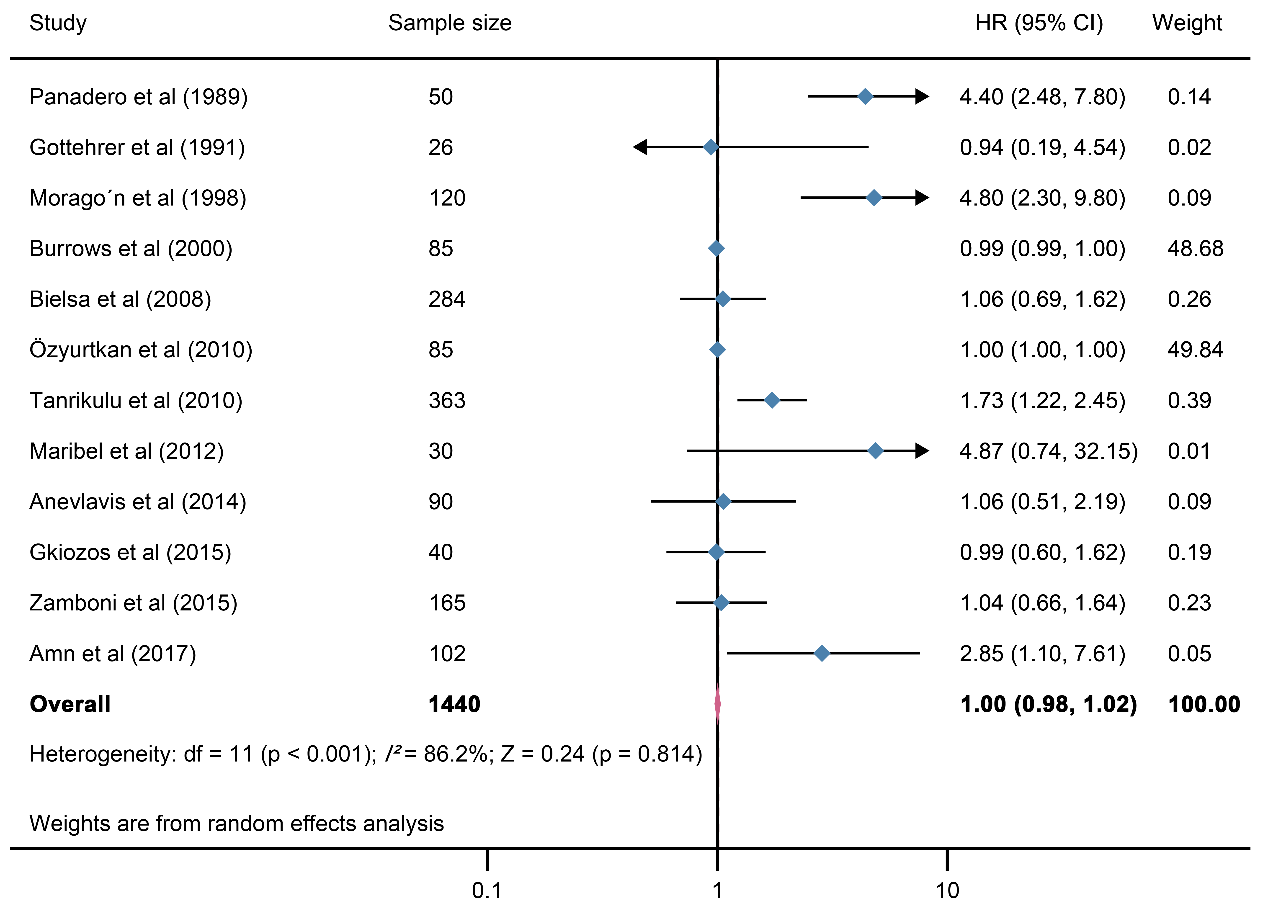


**eFigure 31. Forest plot of the hazard ratios of pleural effusion LDH for overall survival in malignant patients.**


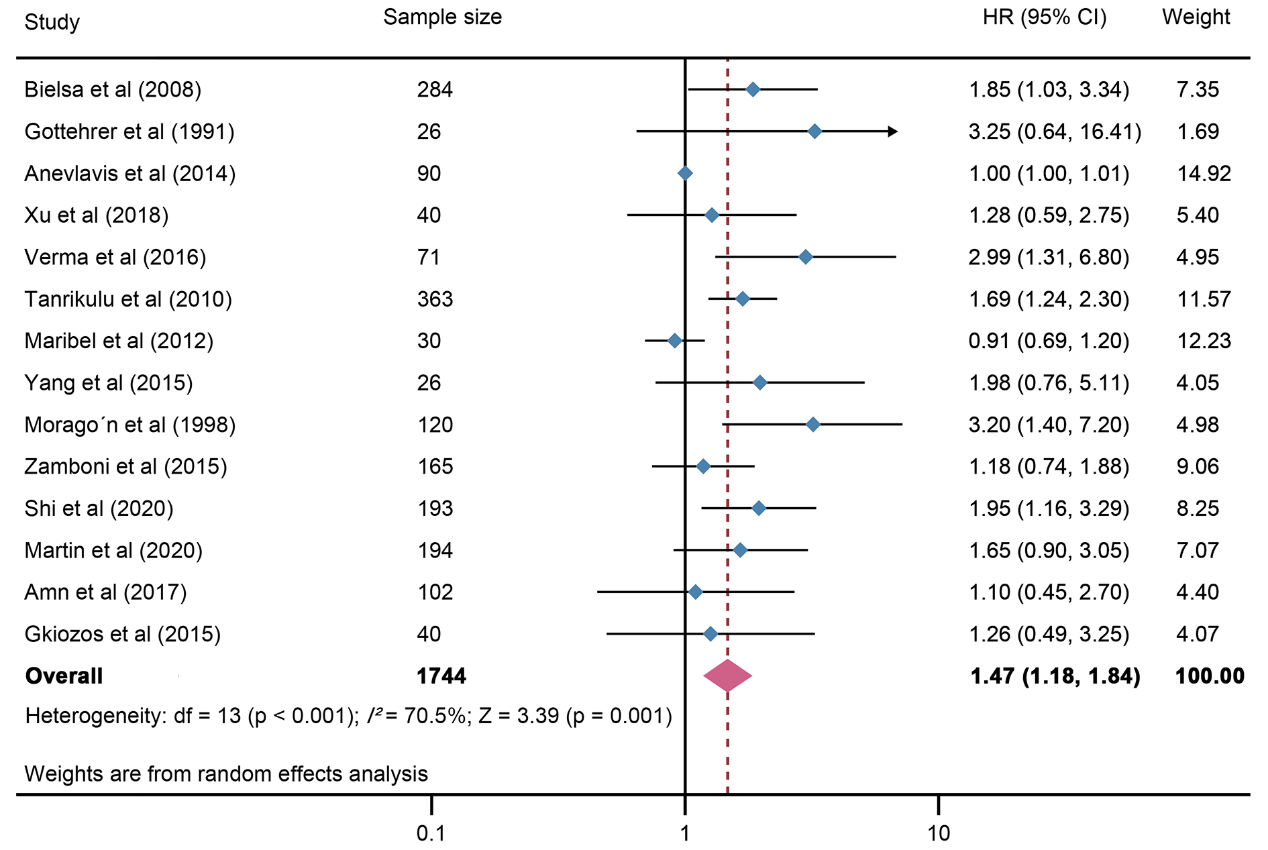


**eFigure 32. Forest plot of the hazard ratios of** **pleural effusion VEGF for overall survival in malignant patients.**


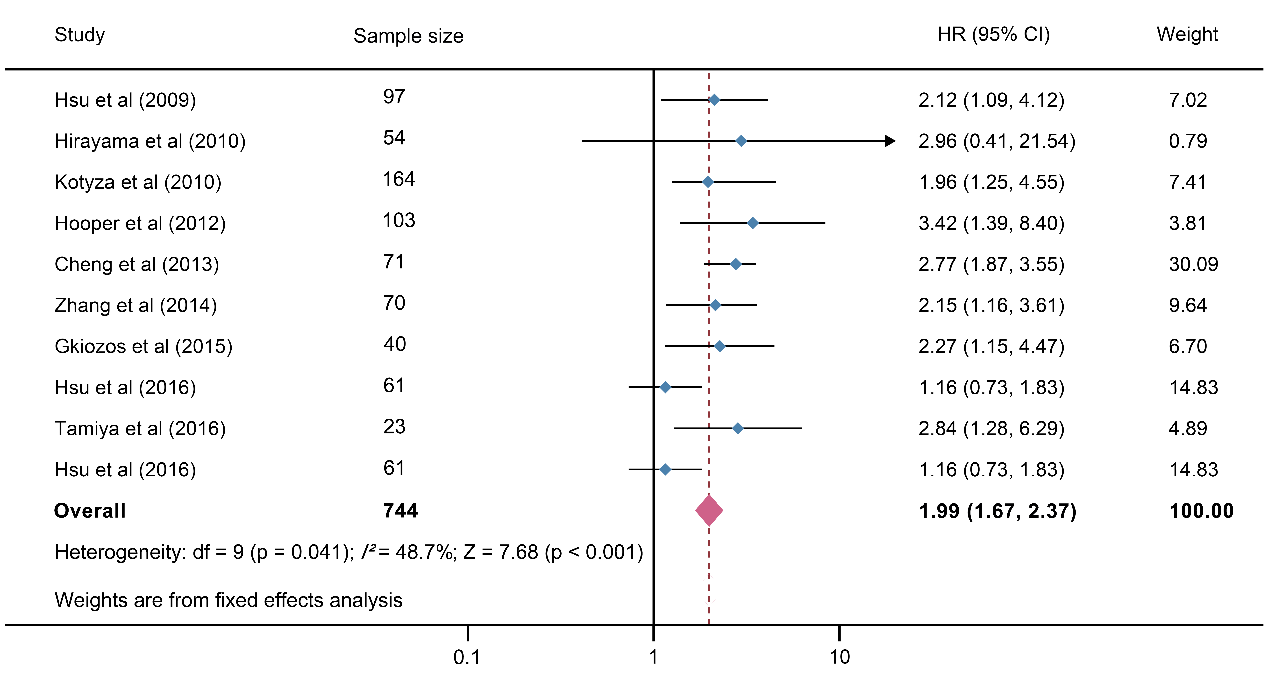


**eFigure 33. Forest plot of the hazard ratios of pleural effusion VEGF for progression-free survival in malignant patients.**


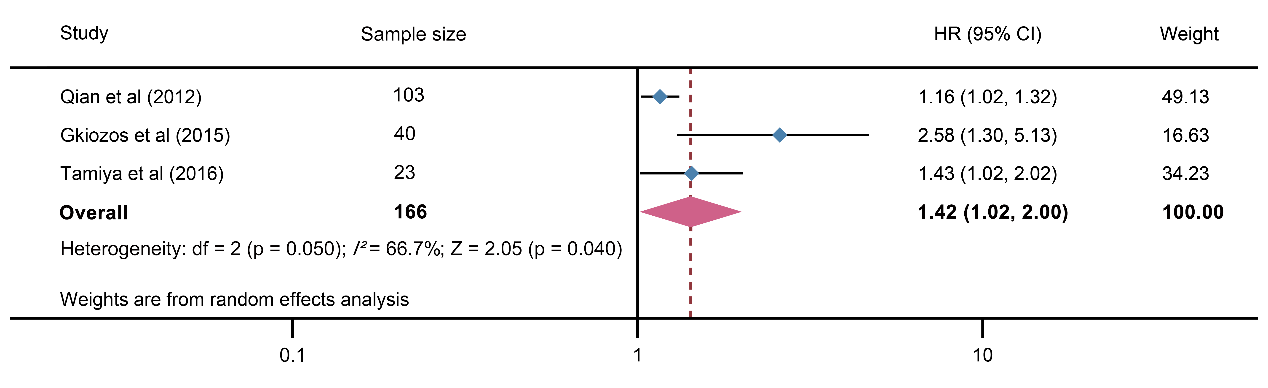


**eFigure 34. Forest plot of the hazard ratios of pleural effusion survivin for overall survival in malignant patients.**


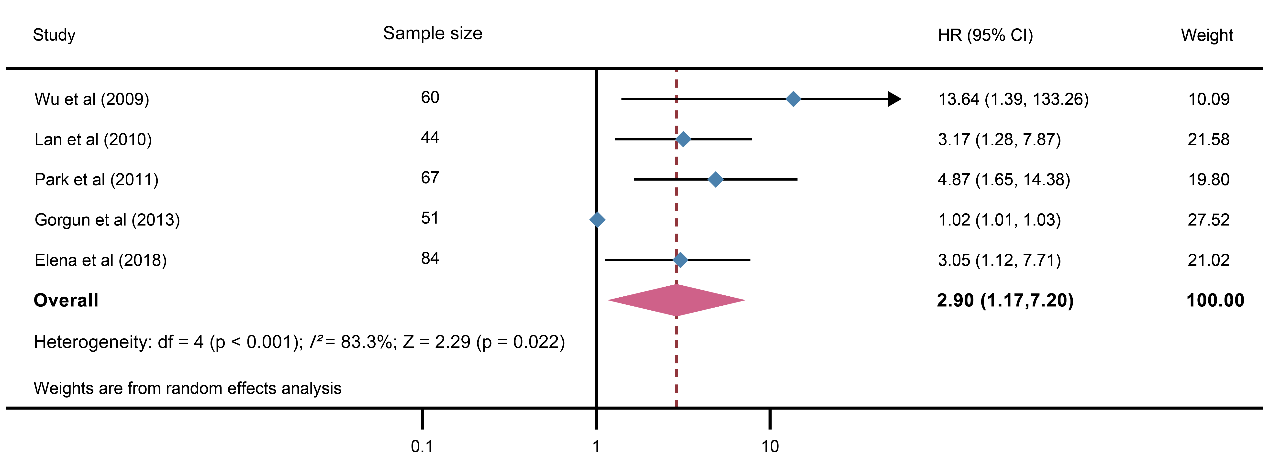


**eFigure 35.** **Subgroup analysis of age for overall survival in malignant patients** **by cancer type.**

**eFigure 36. Subgroup analysis of gender for overall survival in** **malignant patients by cancer type.**

**eFigure 37. Subgroup analysis of smoking for overall survival in** **malignant patients by cancer type.**

**eFigure 38. Subgroup analysis of ECOG PS for overall survival in malignant patients by cancer type.**

**eFigure 39. Subgroup analysis of cytology for overall survival in malignant patients by cancer type.**

**eFigure 40. Subgroup analysis of distant metastasis for overall survival** **in malignant patients by cancer type.**

**eFigure 41. Subgroup analysis of pleural effusion VEGF for overall** **survival in malignant patients by cancer type.**

**eFigure 42. Sensitivity analysis of hazard ratios of age for overall survival in malignant patients with pleural effusion.**


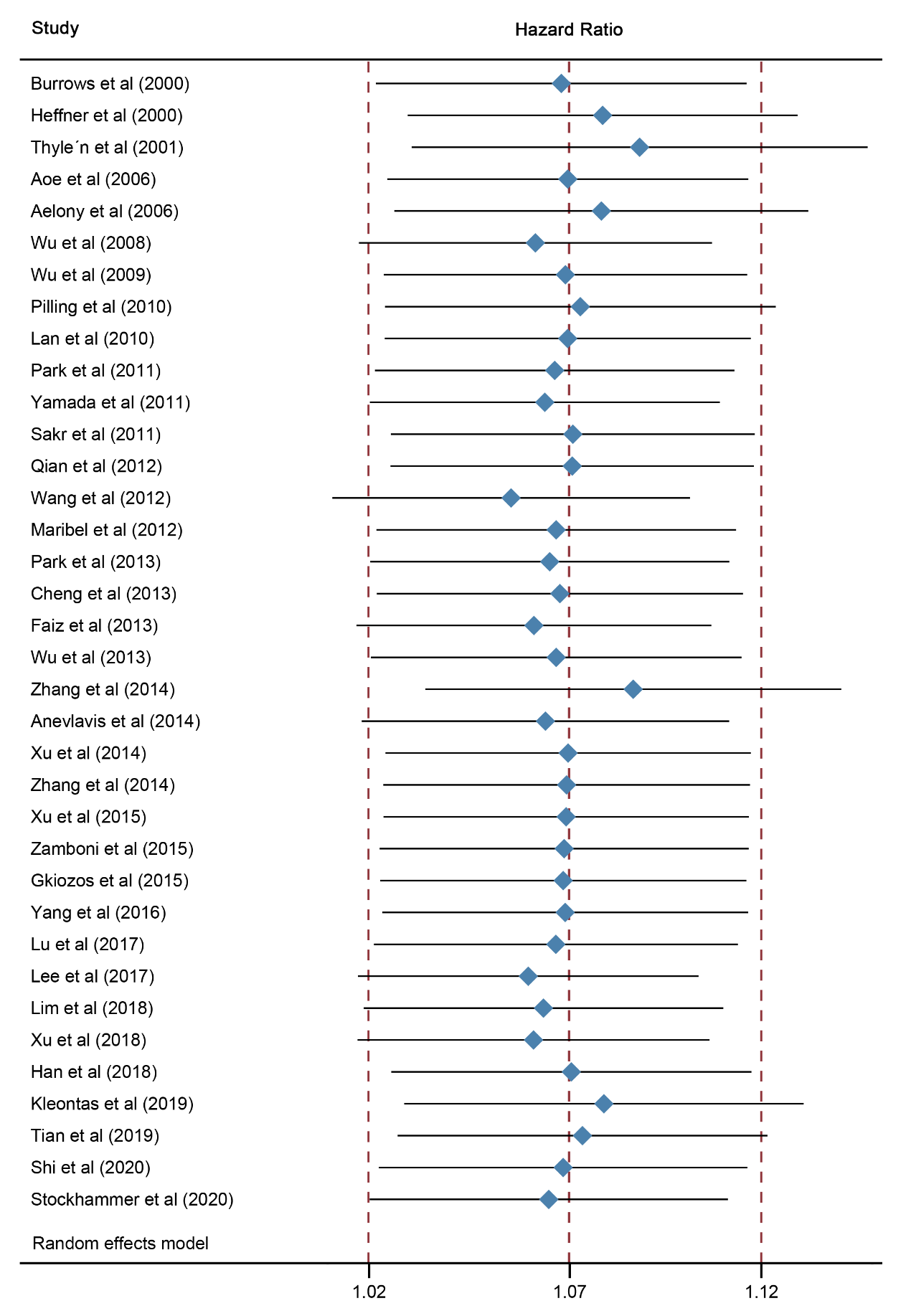


**eFigure 43. Sensitivity analysis of hazard ratios of smoking status for overall survival in malignant patients with pleural effusion.**


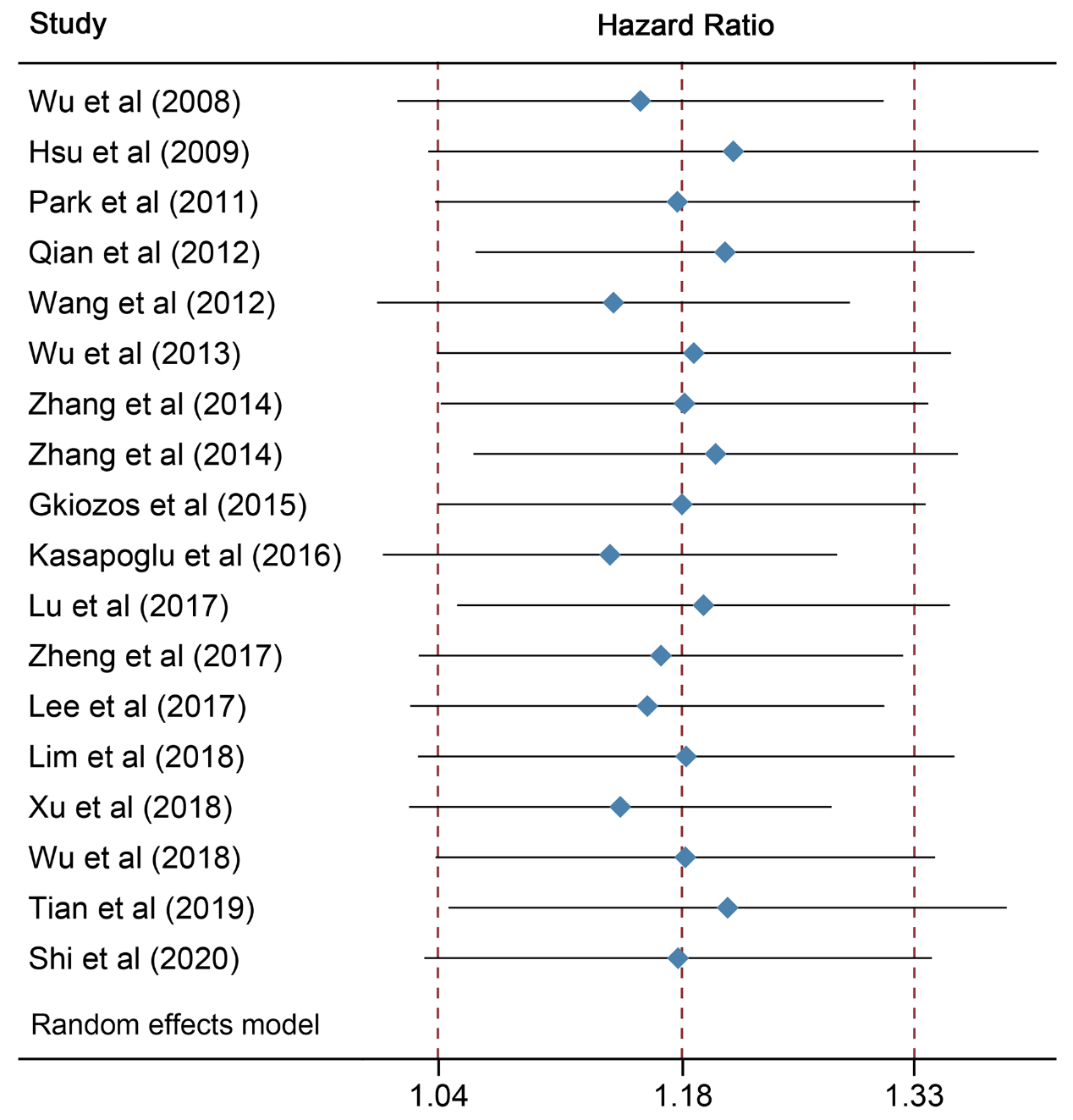


**eFigure 44. Sensitivity analysis of hazard ratios of ECOG PS for overall survival in malignant patients with pleural effusion.**


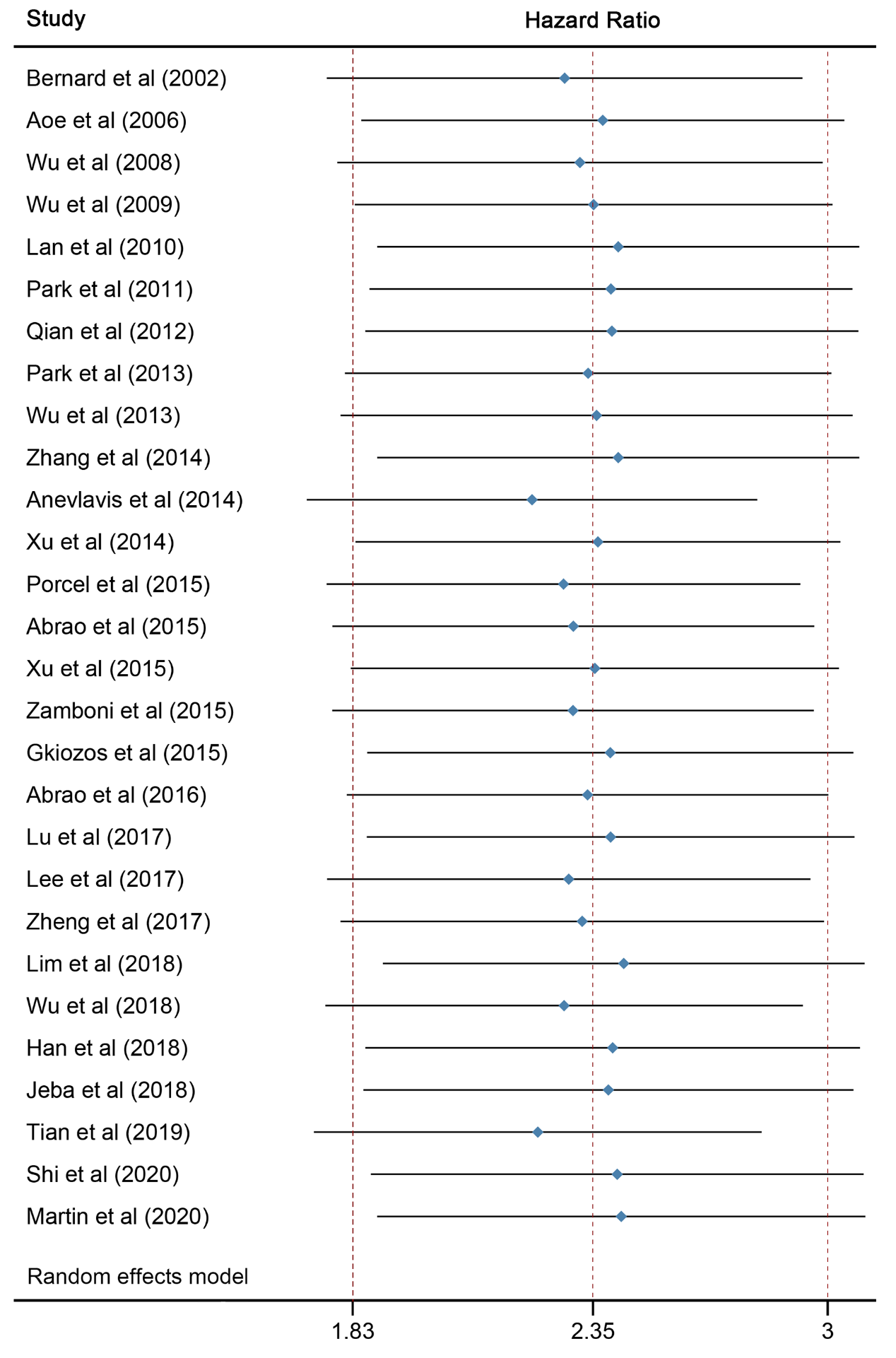


**eFigure 45. Sensitivity analysis of hazard ratios of stage for overall survival in malignant patients with pleural effusion.**


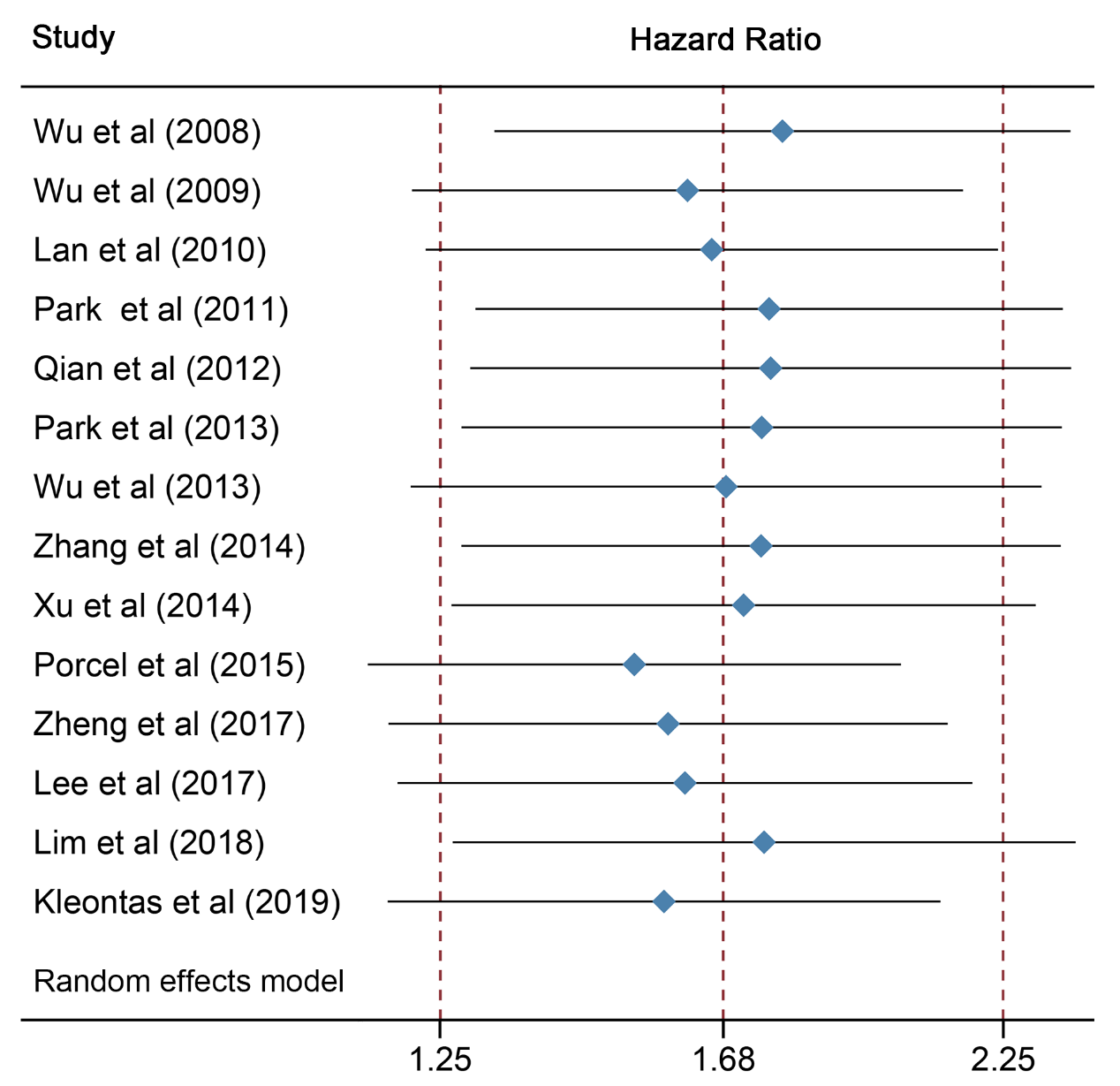


**eFigure 46. Sensitivity analysis of hazard ratios of histology for overall survival in malignant patients with pleural effusion.**


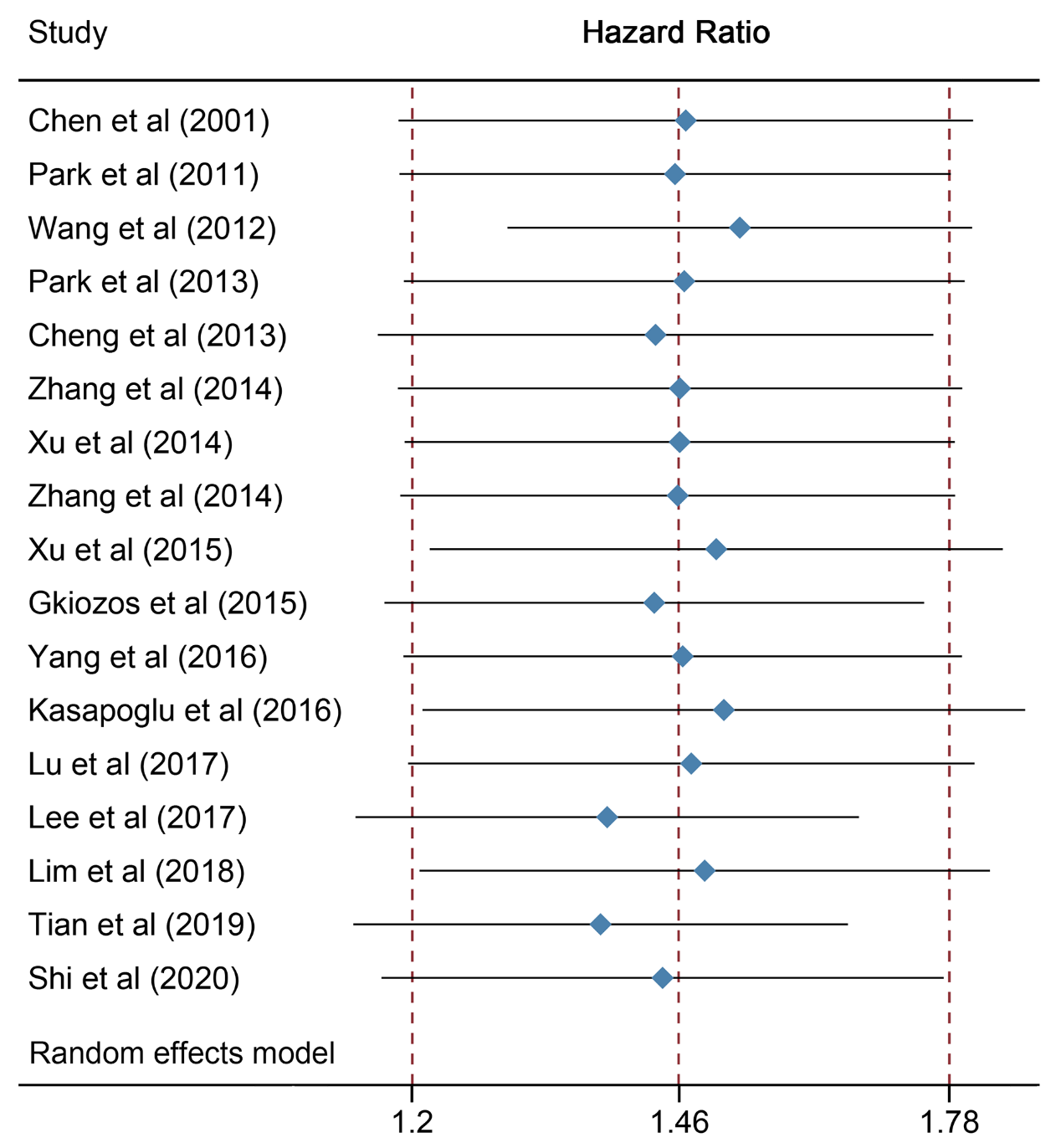


**eFigure 47. Sensitivity analysis of hazard ratios of ECOG PS for progression-free survival in malignant patients with pleural effusion.**


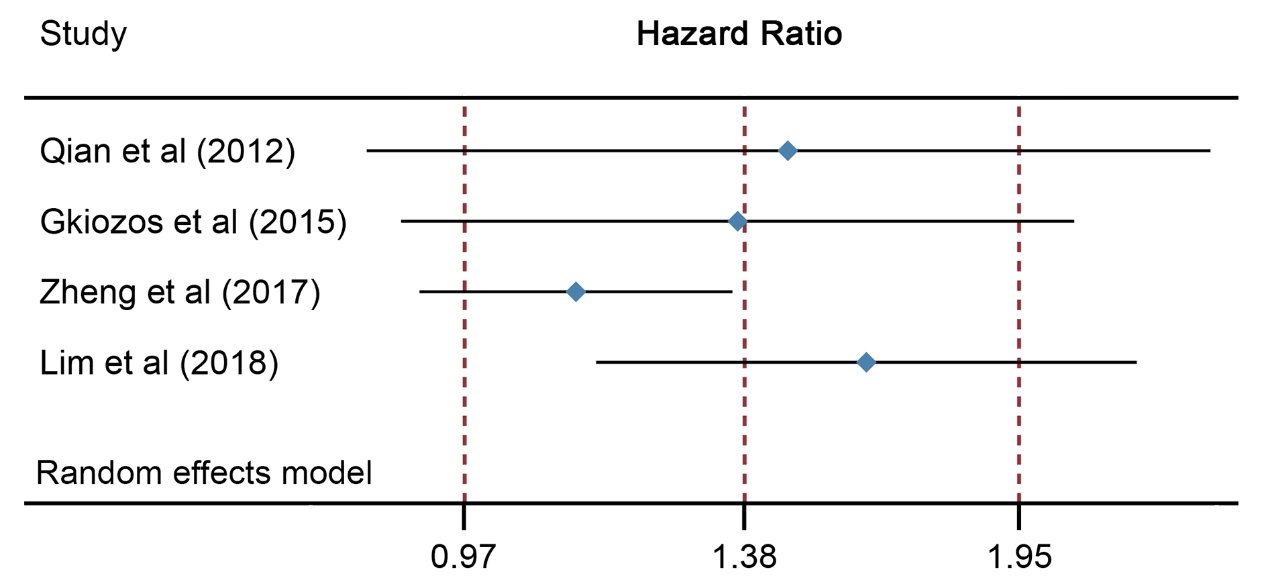


**eFigure 48. Sensitivity analysis of hazard ratios of stage for progression-free survival in malignant patients with pleural effusion.**


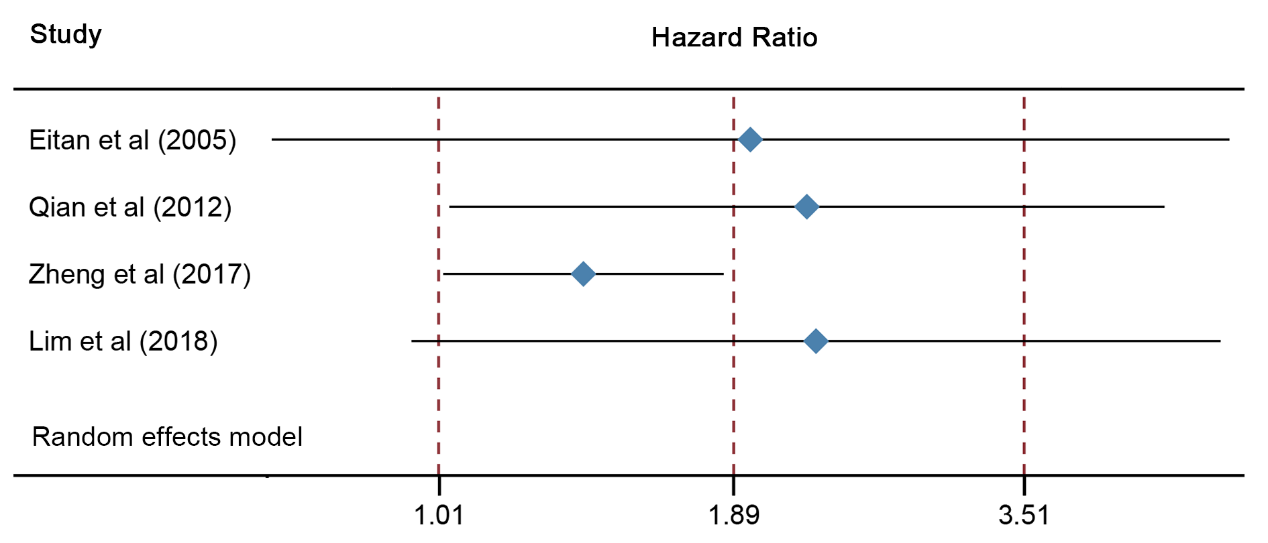


**eFigure 49. Sensitivity analysis of hazard ratios of serum WBC for overall survival in malignant patients with pleural effusion.**


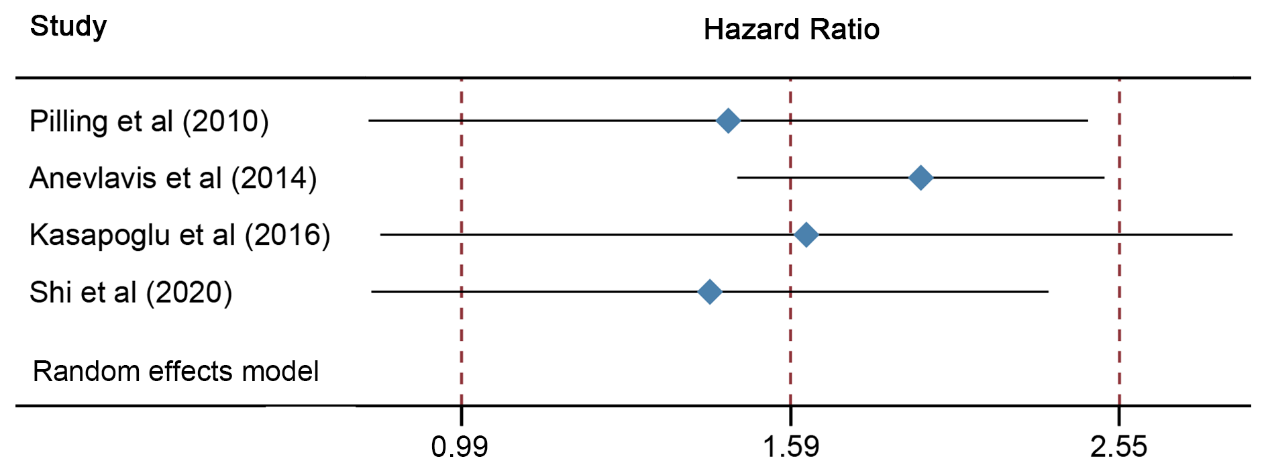


**eFigure 50. Sensitivity analysis of hazard ratios of serum NLR for overall survival in malignant patients with pleural effusion.**


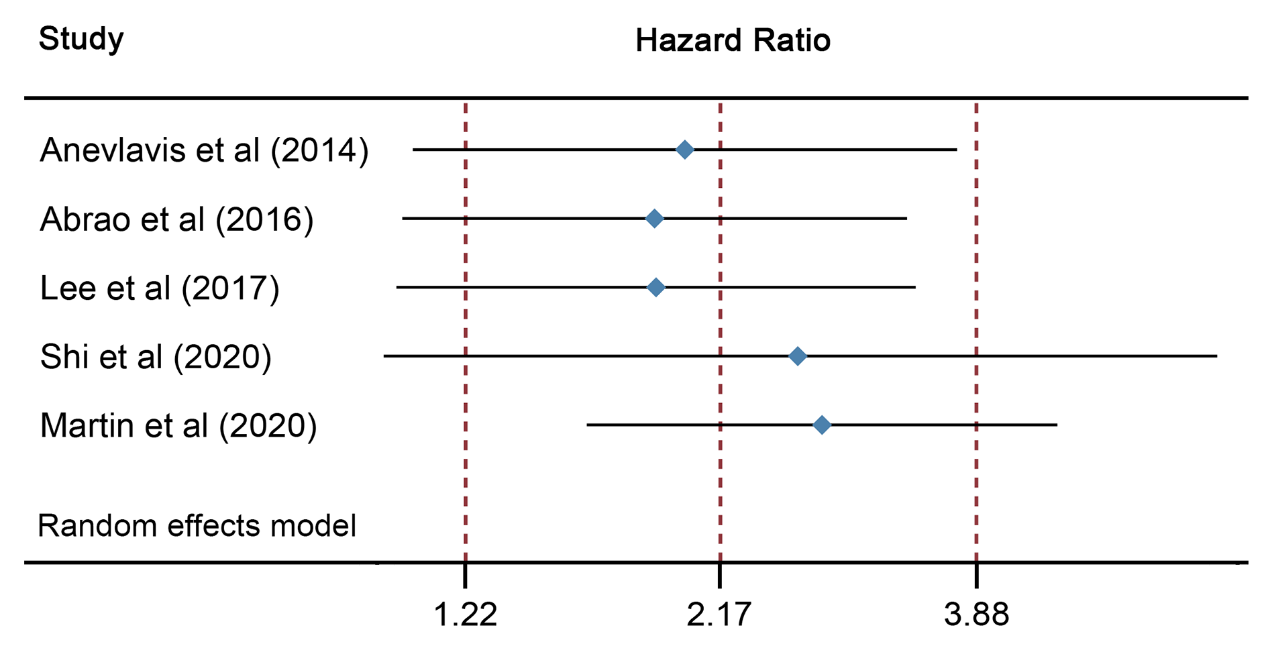


**eFigure 51. Sensitivity analysis of hazard ratios of serum albumin for overall survival in malignant patients with pleural effusion.**


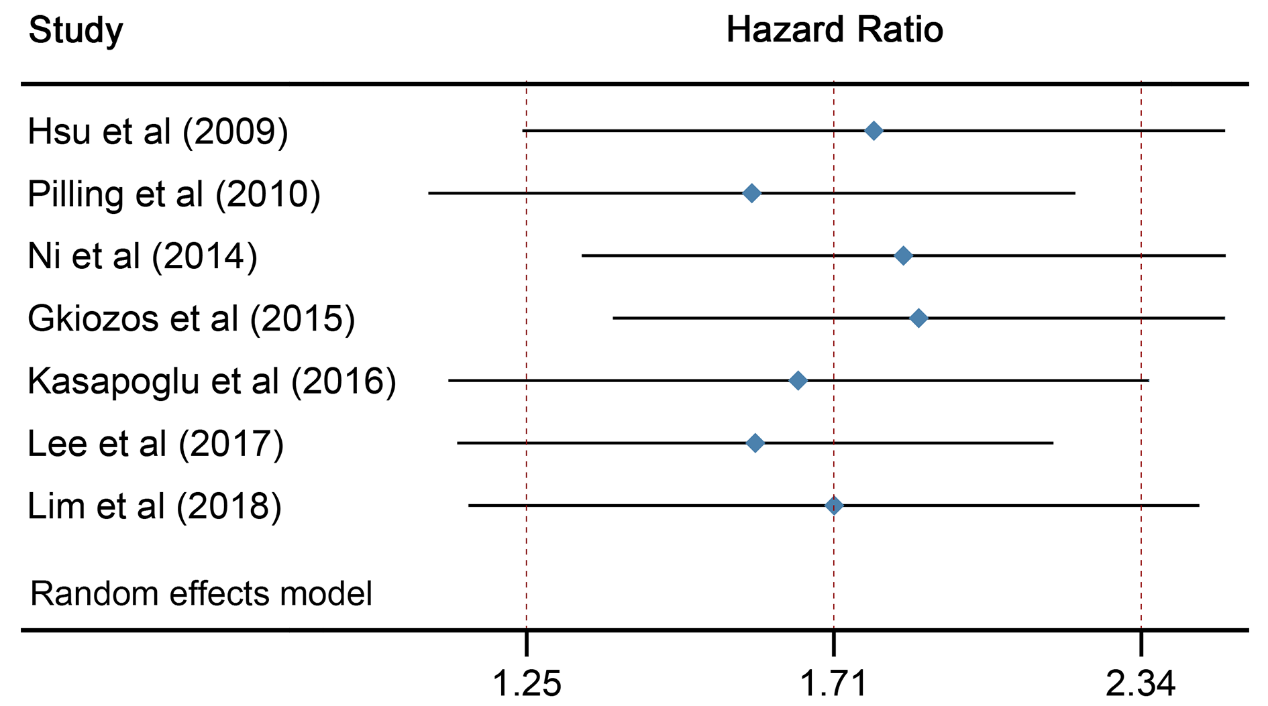


**eFigure 52. Sensitivity analysis of hazard ratios of serum LDH for overall survival in malignant patients with pleural effusion.**


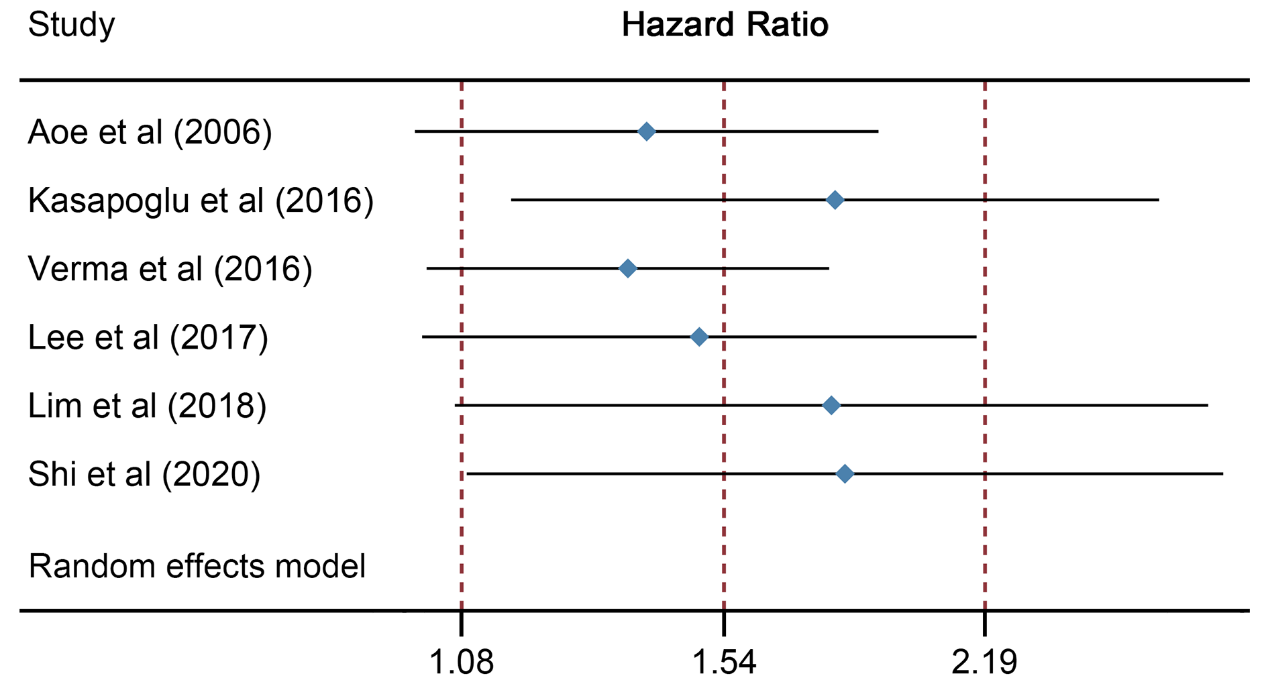


**eFigure 53. Sensitivity analysis of hazard ratios of serum VEGF for progression-free survival in malignant patients with pleural effusion.**


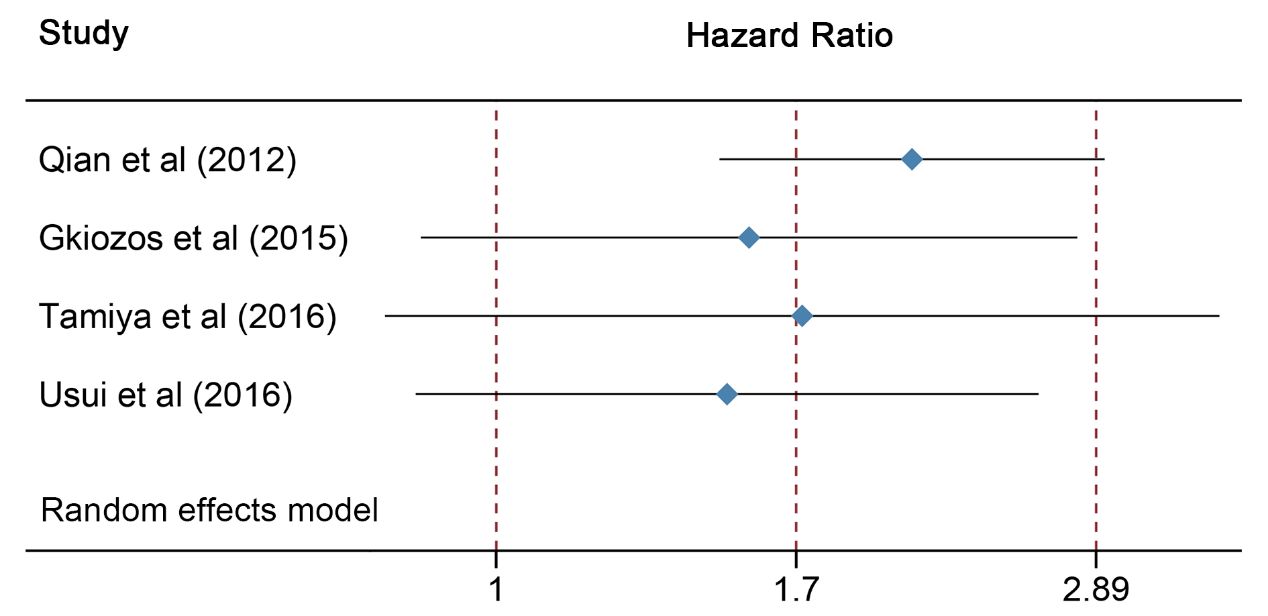


**eFigure 54. Sensitivity analysis of hazard ratios of pleural effusion PH for overall survival in malignant patients.**


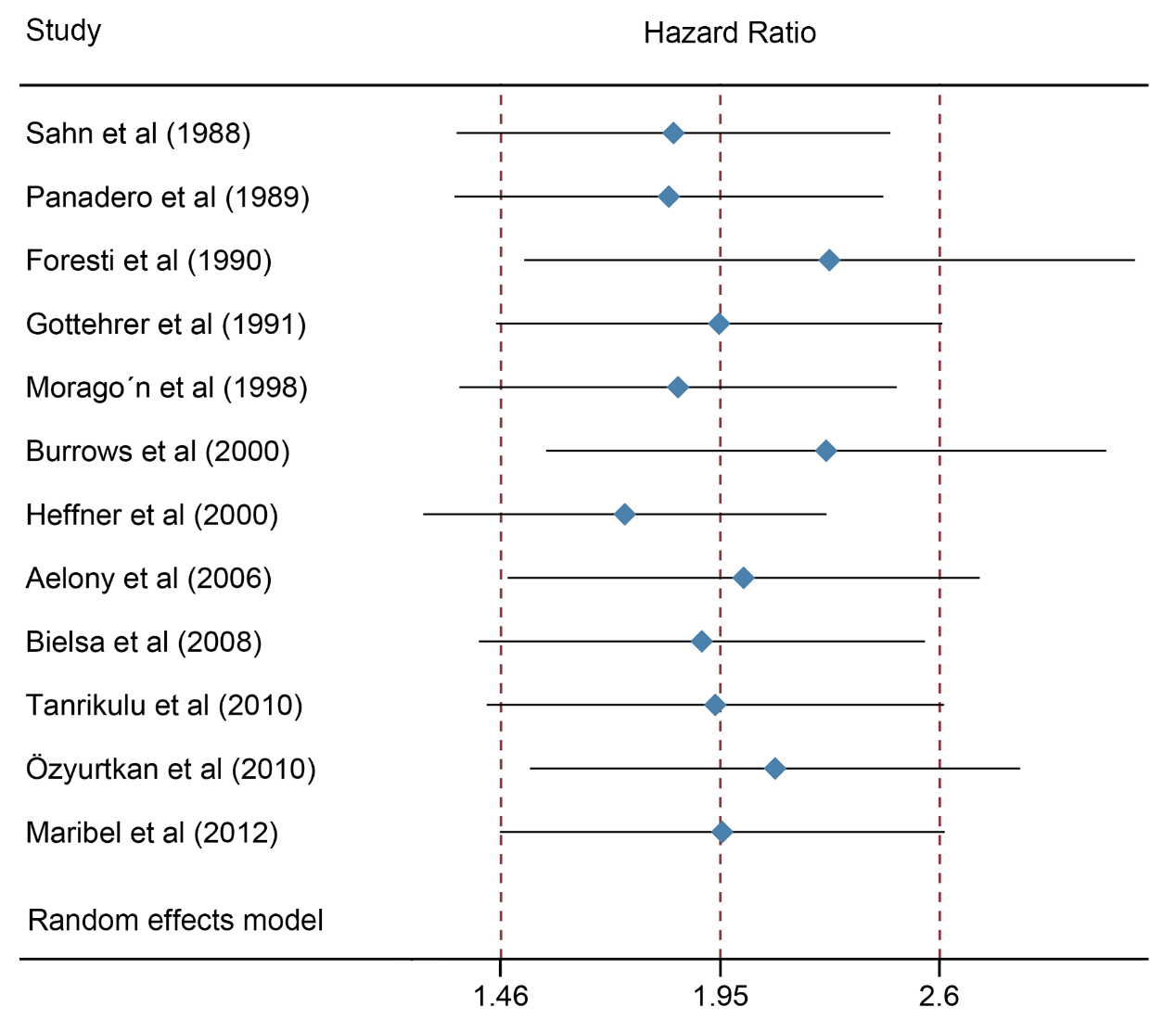


**eFigure 55. Sensitivity analysis of hazard ratios of pleural effusion total protein for overall survival in malignant patients.**


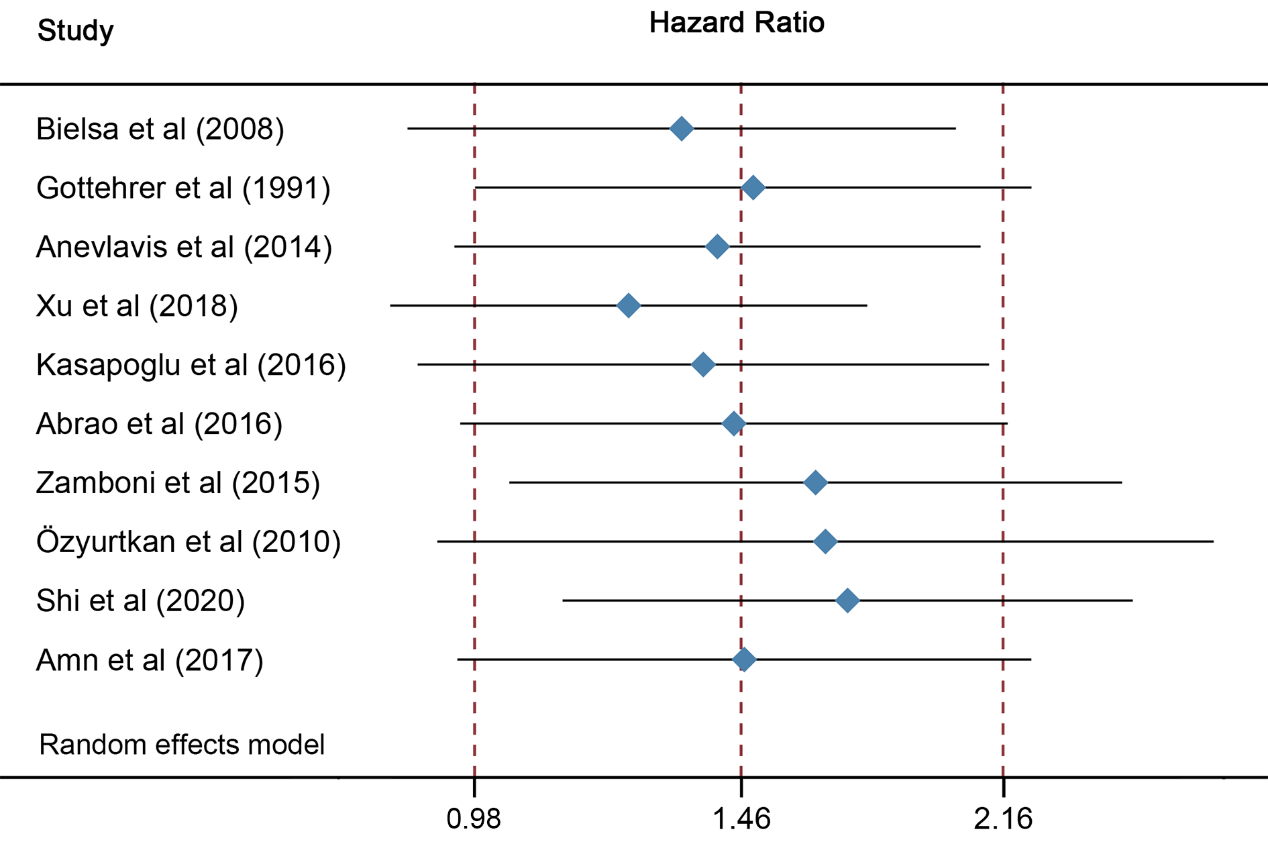


**eFigure 56. Sensitivity analysis of hazard ratios of pleural effusion glucose for overall survival in malignant patients.**


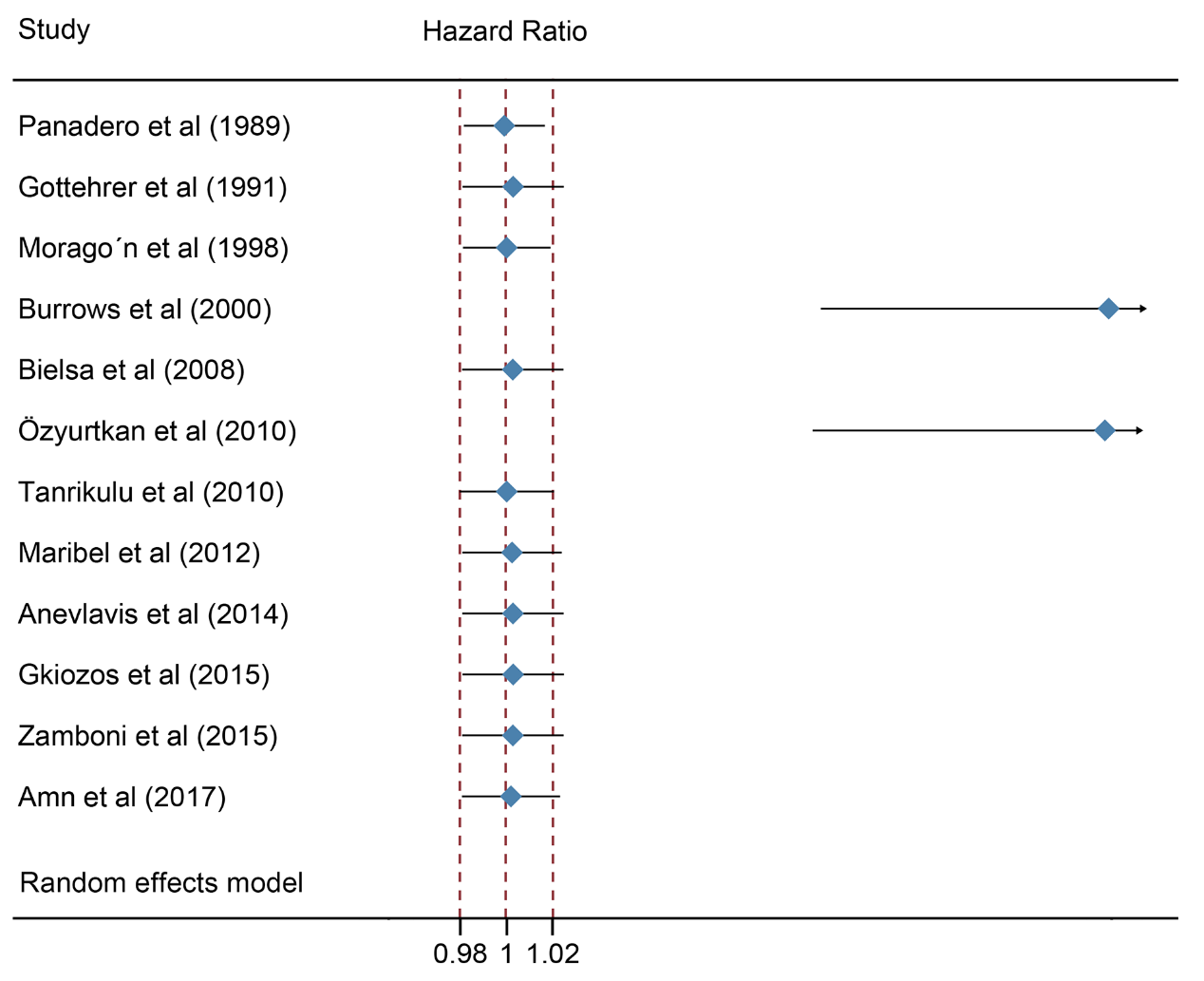


**eFigure 57. Sensitivity analysis of hazard ratios of pleural effusion LDH for overall survival in malignant patients.**


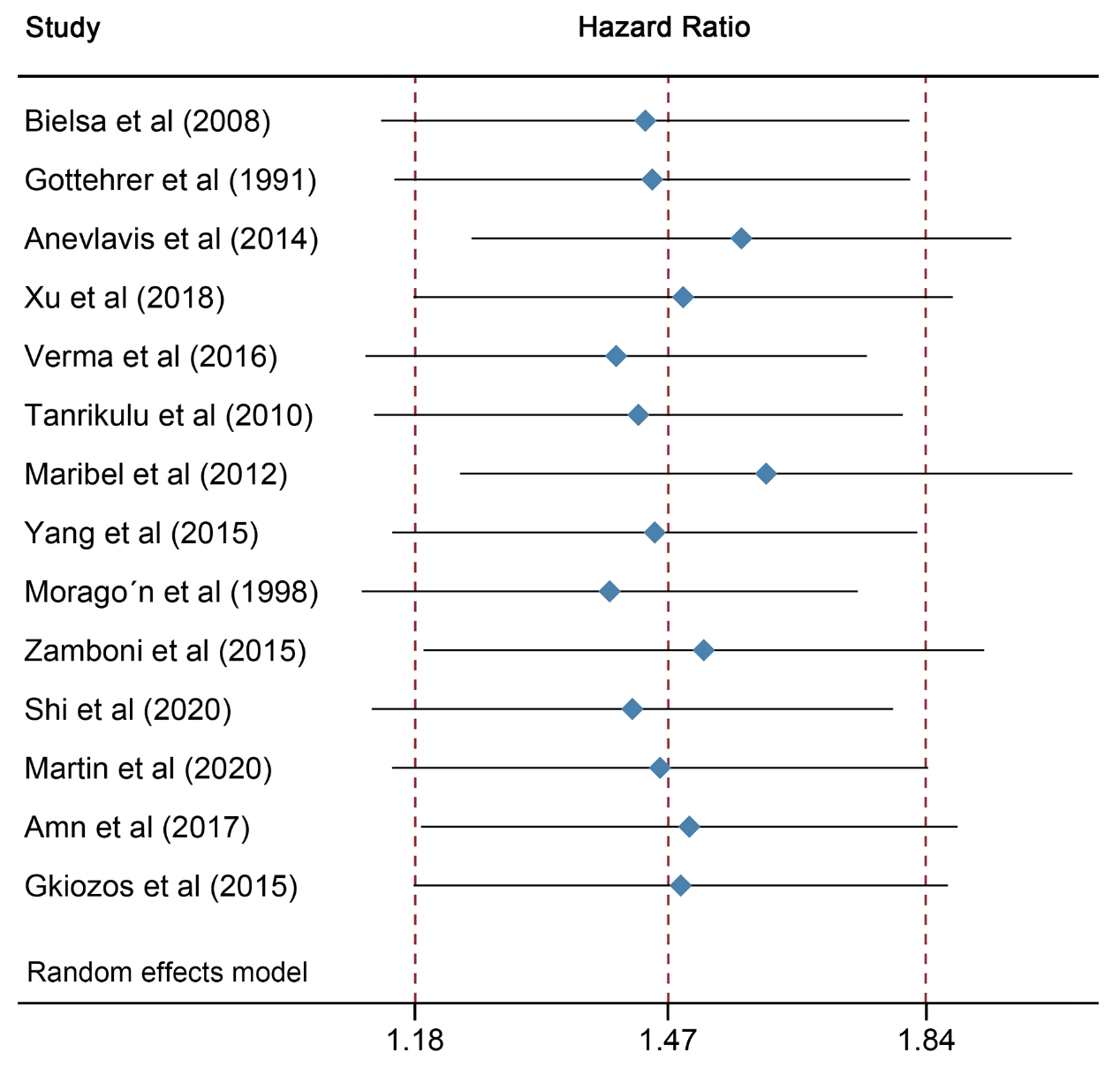


**eFigure 58. Sensitivity analysis of hazard ratios of pleural effusion VEGF for progression-free survival in malignant patients.**


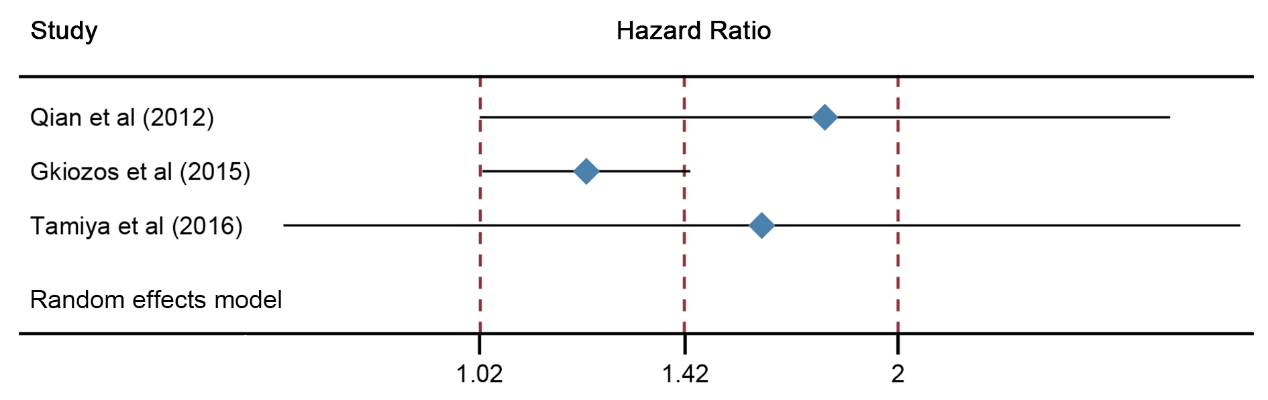


**eFigure 59. Sensitivity analysis of hazard ratios of pleural effusion survivin for overall survival in malignant patients.**


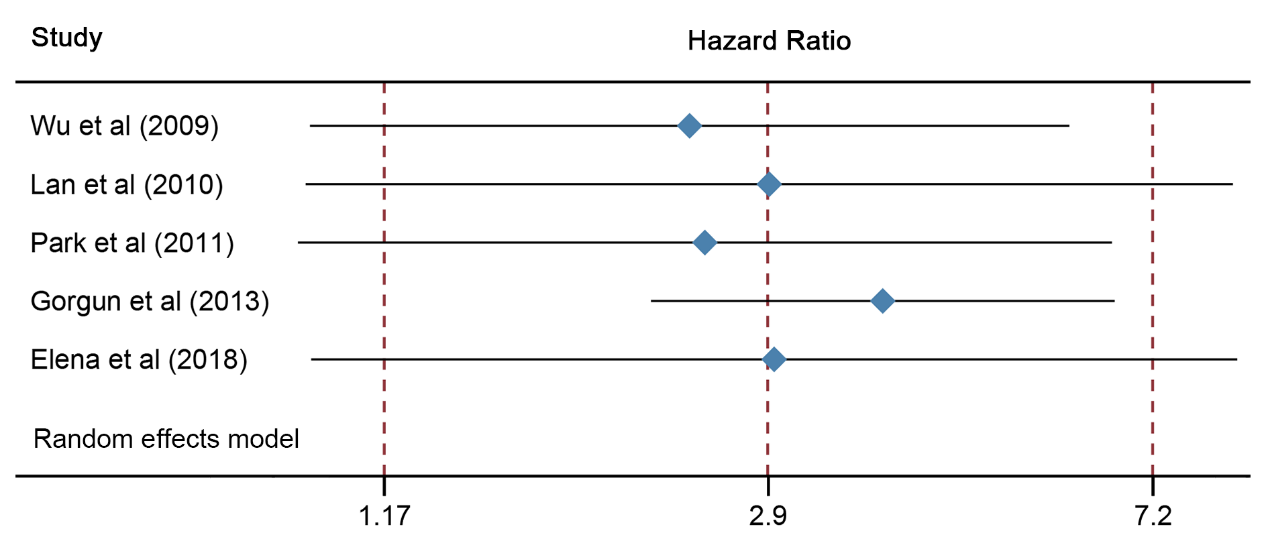


**eFigure 60. Funnel plot and contour-enhanced meta-analysis funnel plot of ln (hazard ratio) of age for overall survival in malignant patients with pleural effusion.**

**
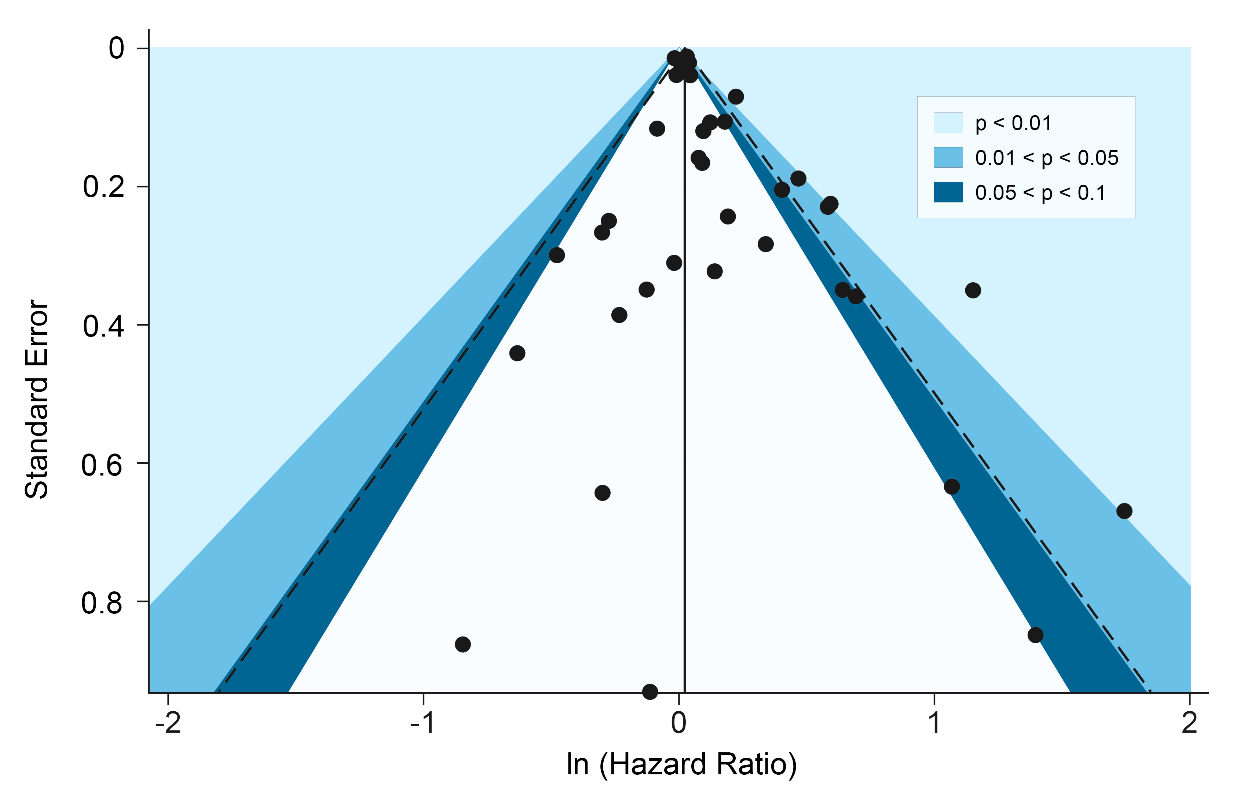
**

**eFigure 61. Funnel plot and contour-enhanced meta-analysis funnel plot of ln (hazard ratio) of gender for overall survival in malignant patients with pleural effusion.**

**
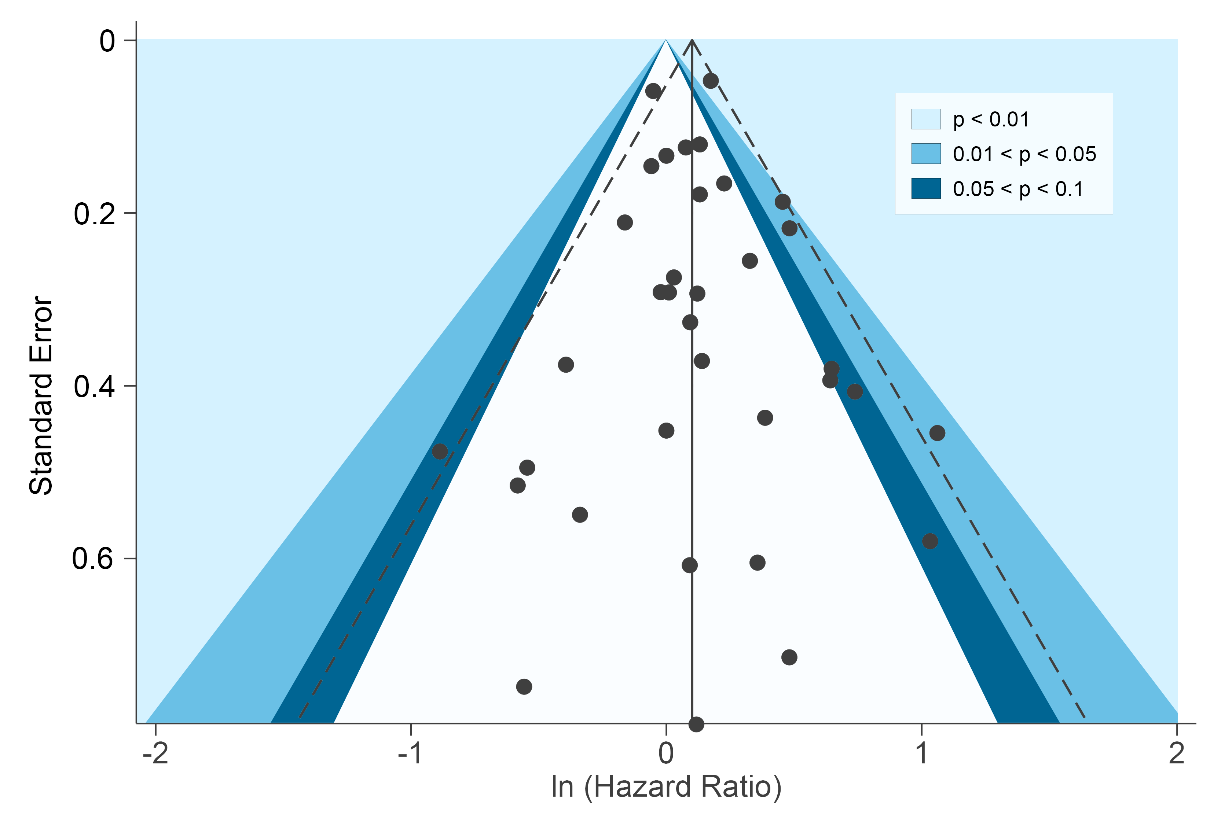
**

**eFigure 62. Funnel plot and contour-enhanced meta-analysis funnel plot of ln (hazard ratio) of smoking status for overall survival in malignant patients with pleural effusion.**

**
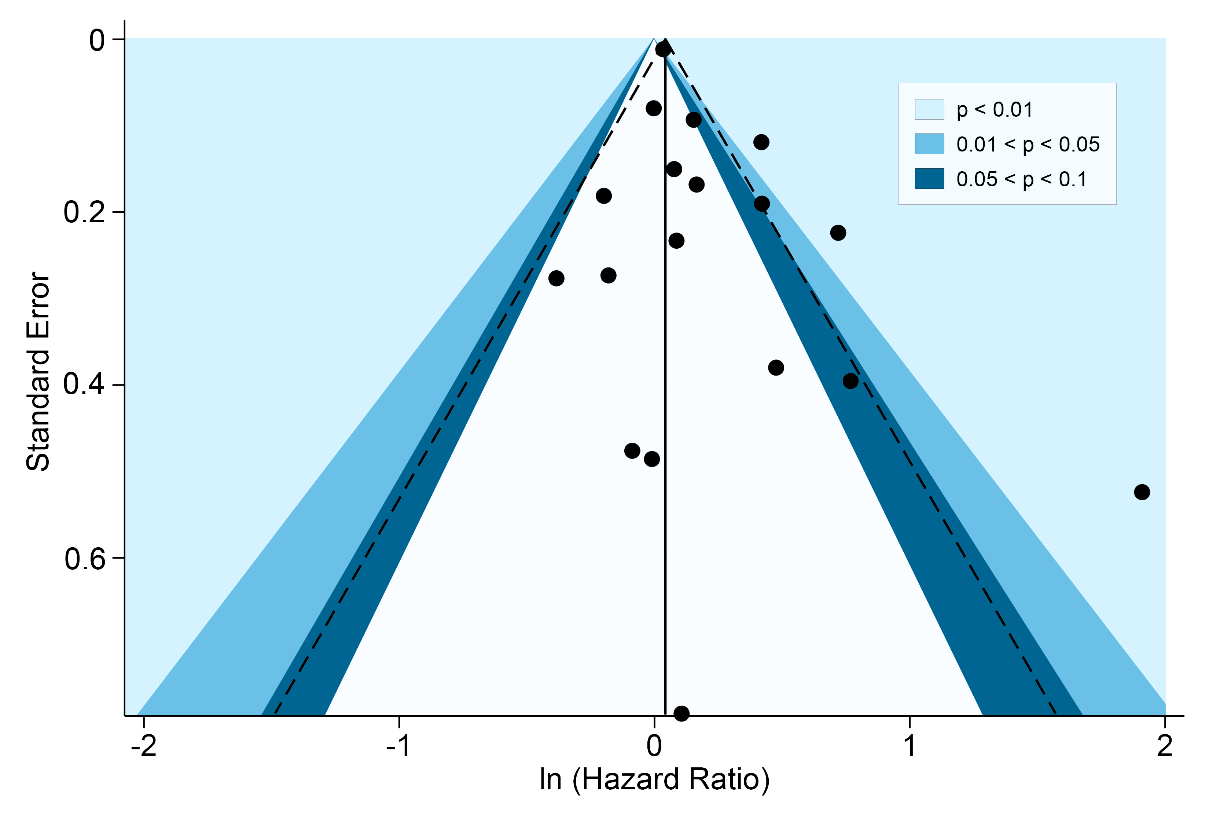
**

**eFigure 63. Funnel plot and contour-enhanced meta-analysis funnel plot of ln (hazard ratio) of ECOG PS for overall survival in malignant patients with pleural effusion.**

**
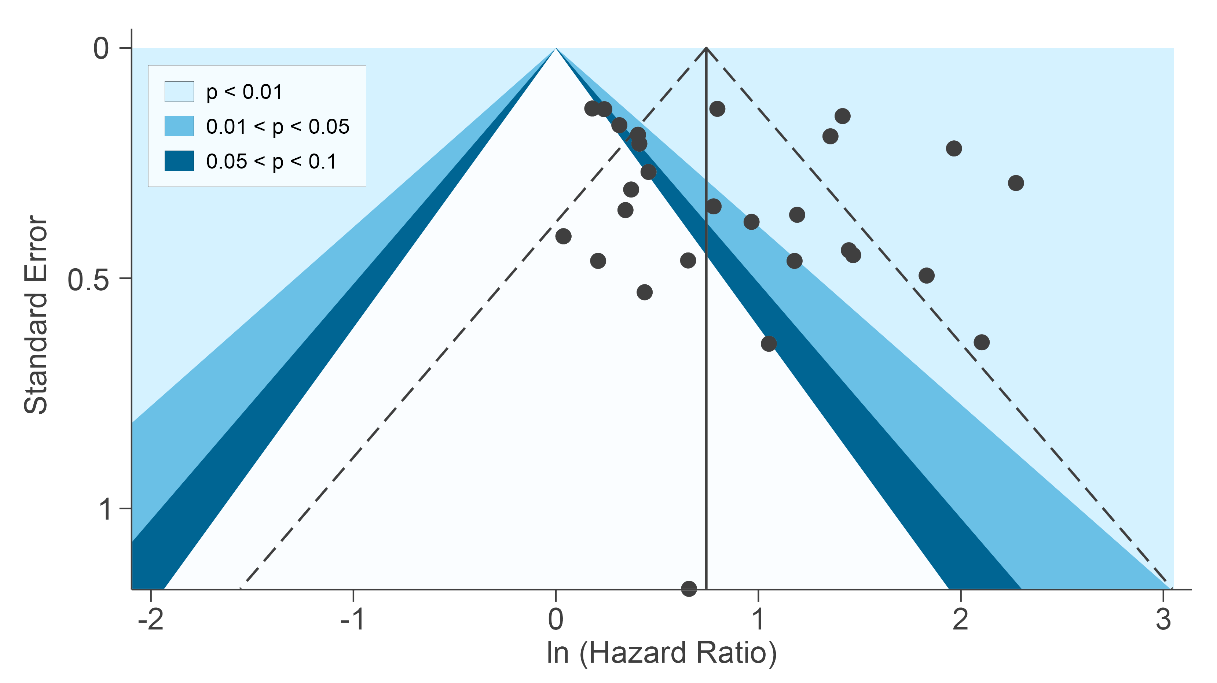
**

**eFigure 64. Funnel plot and contour-enhanced meta-analysis funnel plot of ln (hazard ratio) of stage for overall survival in malignant patients with pleural effusion.**

**
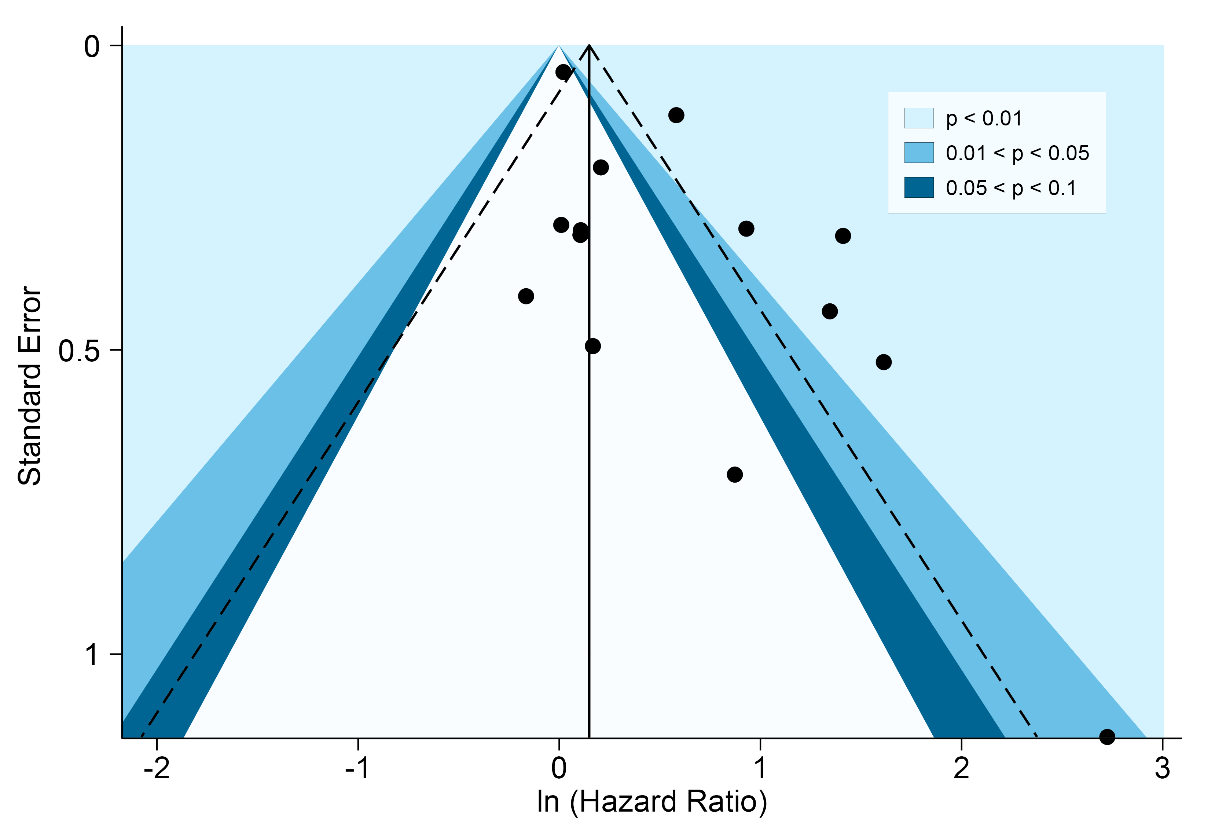
**

**eFigure 65. Funnel plot and contour-enhanced meta-analysis funnel plot of ln (hazard ratio) of age for overall survival in malignant patients with pleural effusion.**

**
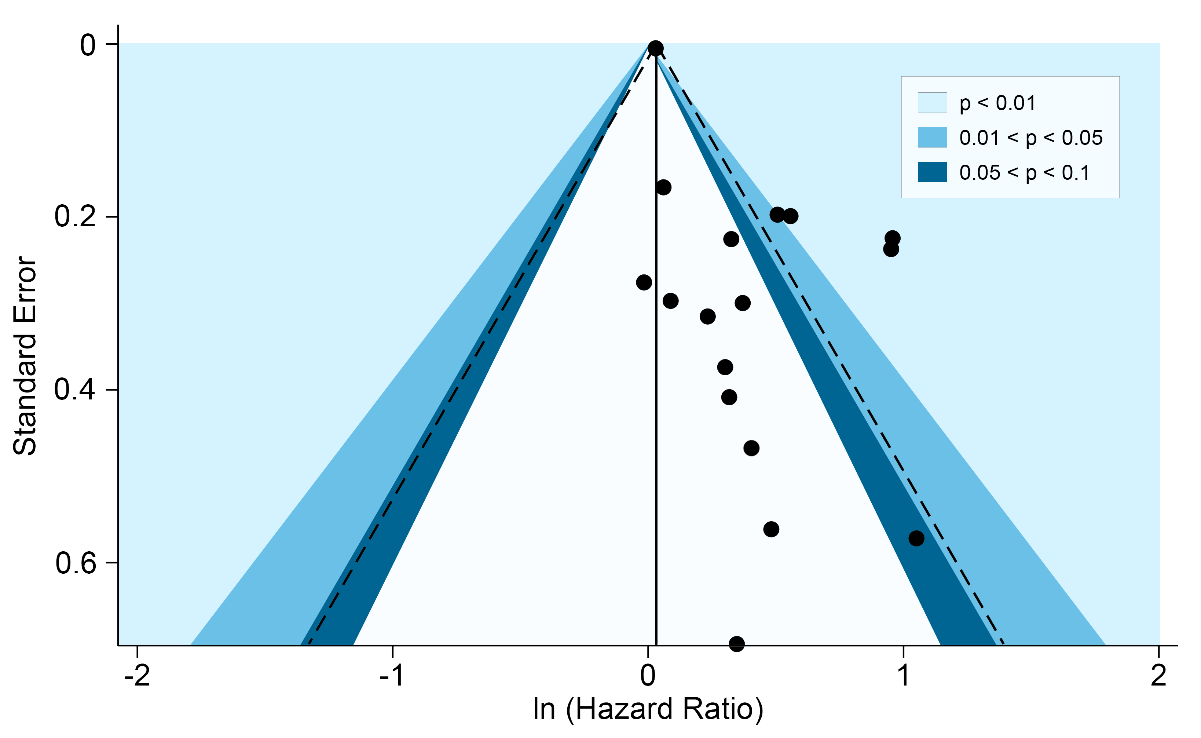
**

**eFigure 66. Funnel plot and contour-enhanced meta-analysis funnel plot of ln (hazard ratio) of distant metastasis for overall survival in malignant patients with pleural effusion.**

**
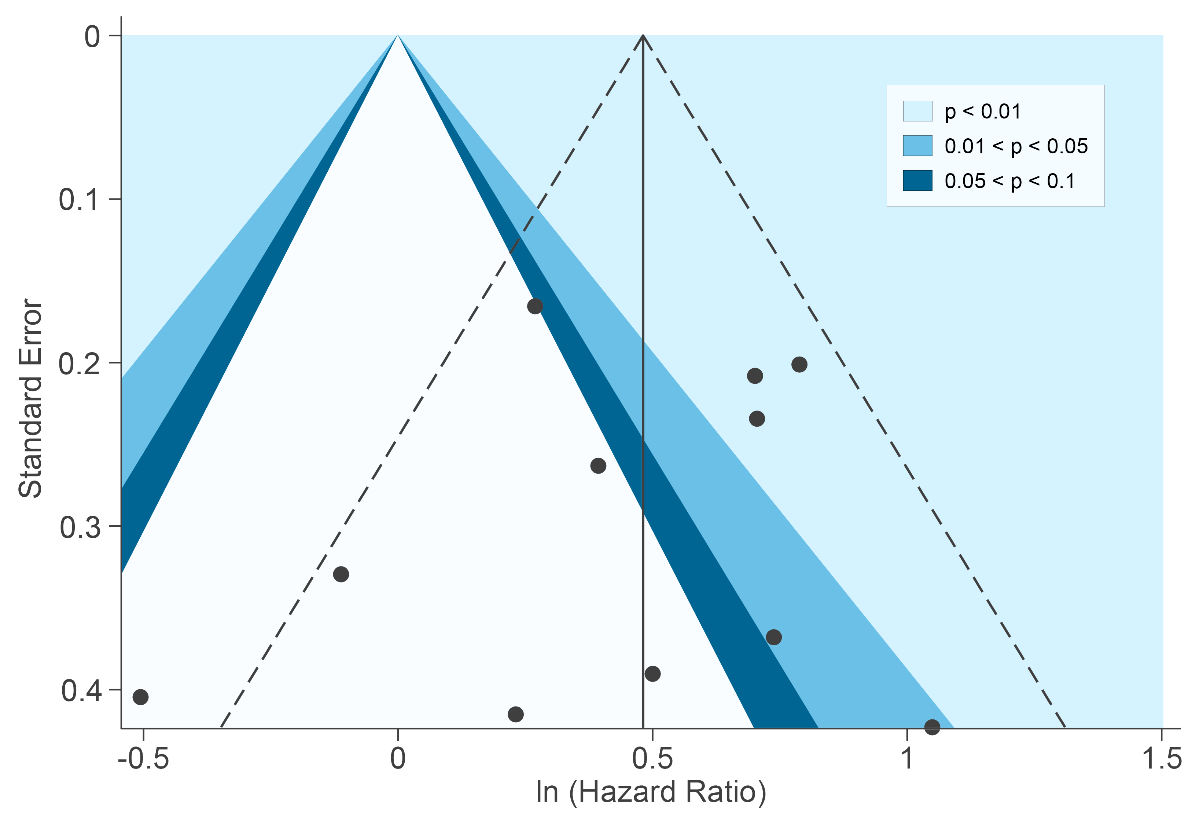
**

**eFigure 67. Funnel plot and contour-enhanced meta-analysis funnel plot of ln (hazard ratio) of pleural effusion PH for overall survival in malignant patients.**

**
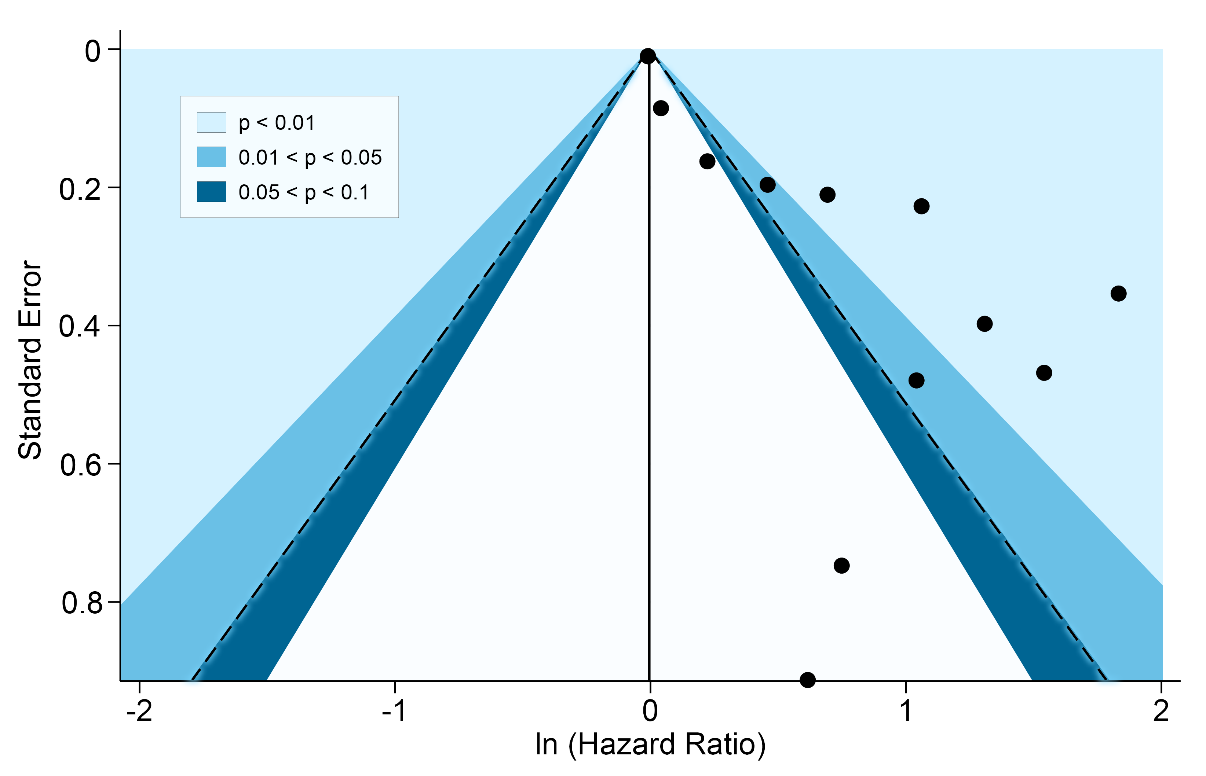
**

**eFigure 68. Funnel plot and contour-enhanced meta-analysis funnel plot of ln (hazard ratio) of pleural effusion total protein for overall survival in malignant patients.**

**
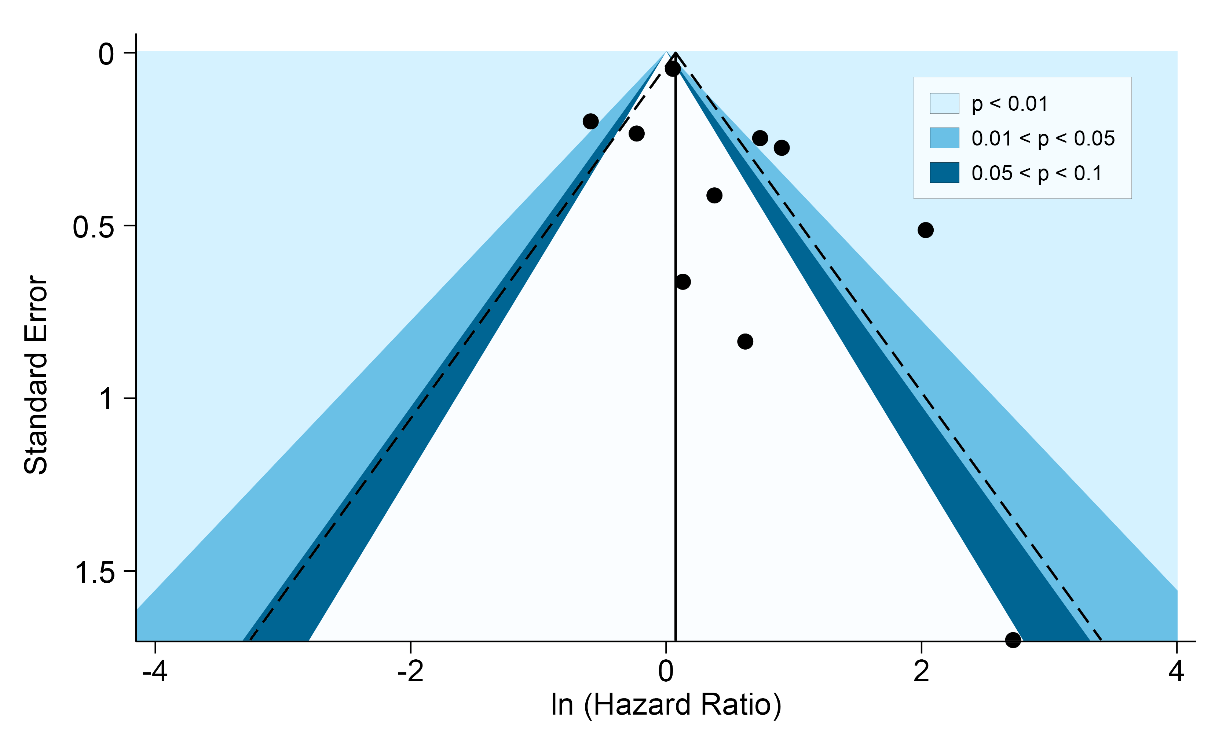
**

**eFigure 69. Funnel plot and contour-enhanced meta-analysis funnel plot of ln (hazard ratio) of pleural effusion glucose for overall survival in malignant patients.**

**
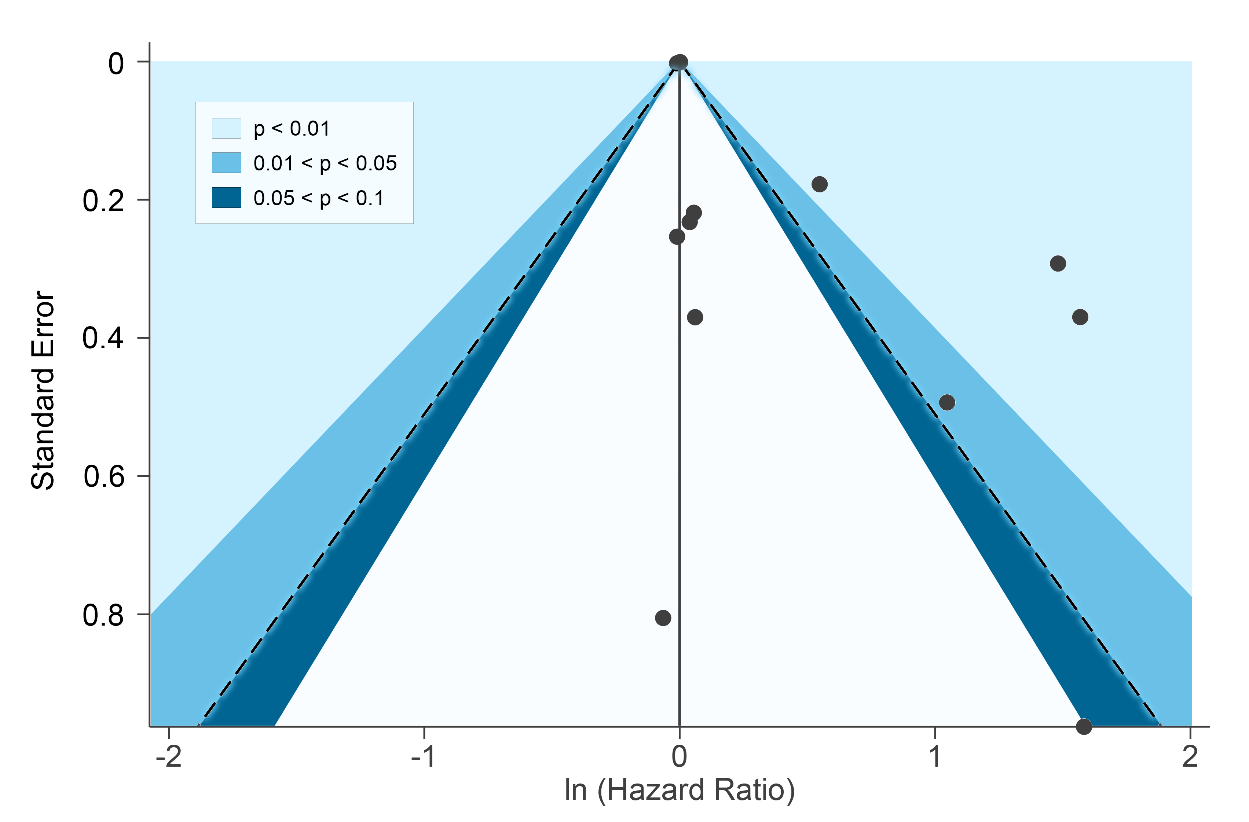
**

**eFigure 70. Funnel plot and contour-enhanced meta-analysis funnel plot of ln (hazard ratio) of pleural effusion LDH for overall survival in malignant patients.**

**
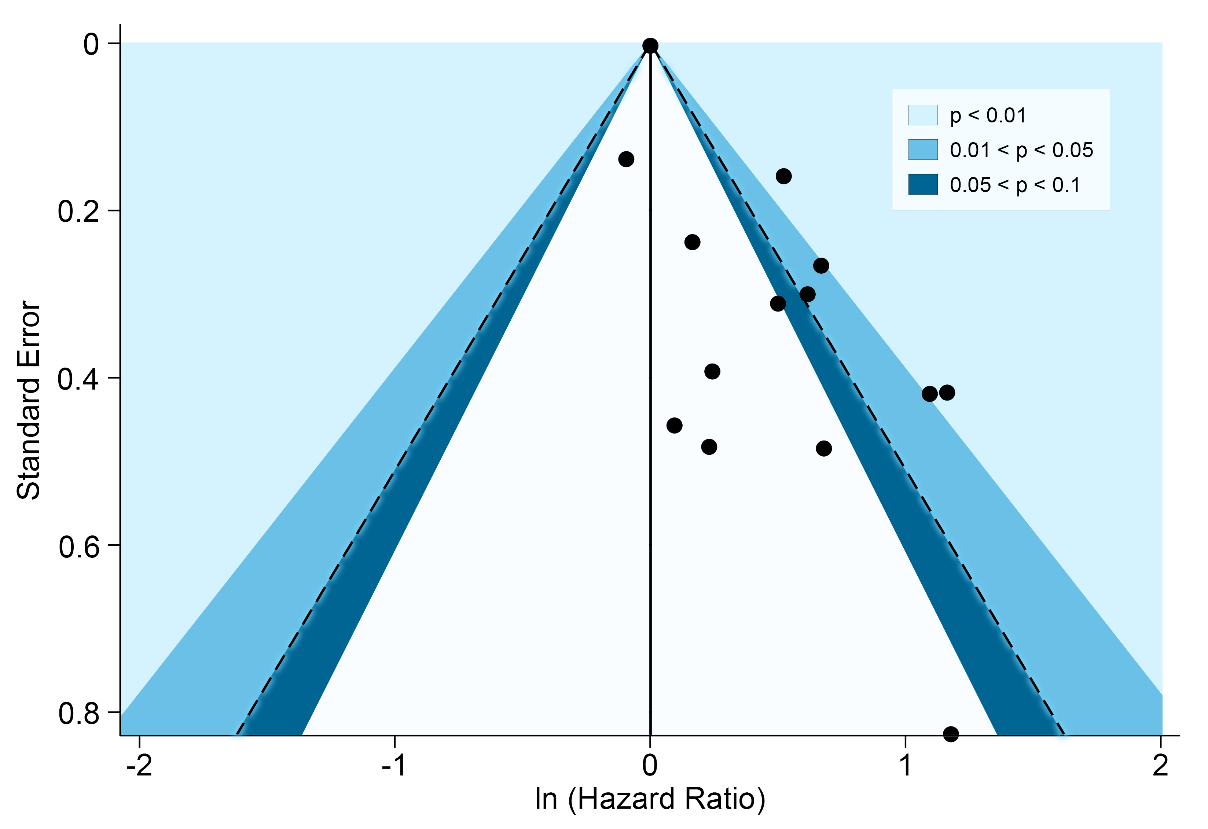
**

**eFigure 71. Funnel plot and contour-enhanced meta-analysis funnel plot of ln (hazard ratio) of pleural effusion VEGF for overall survival in malignant patients.**

**
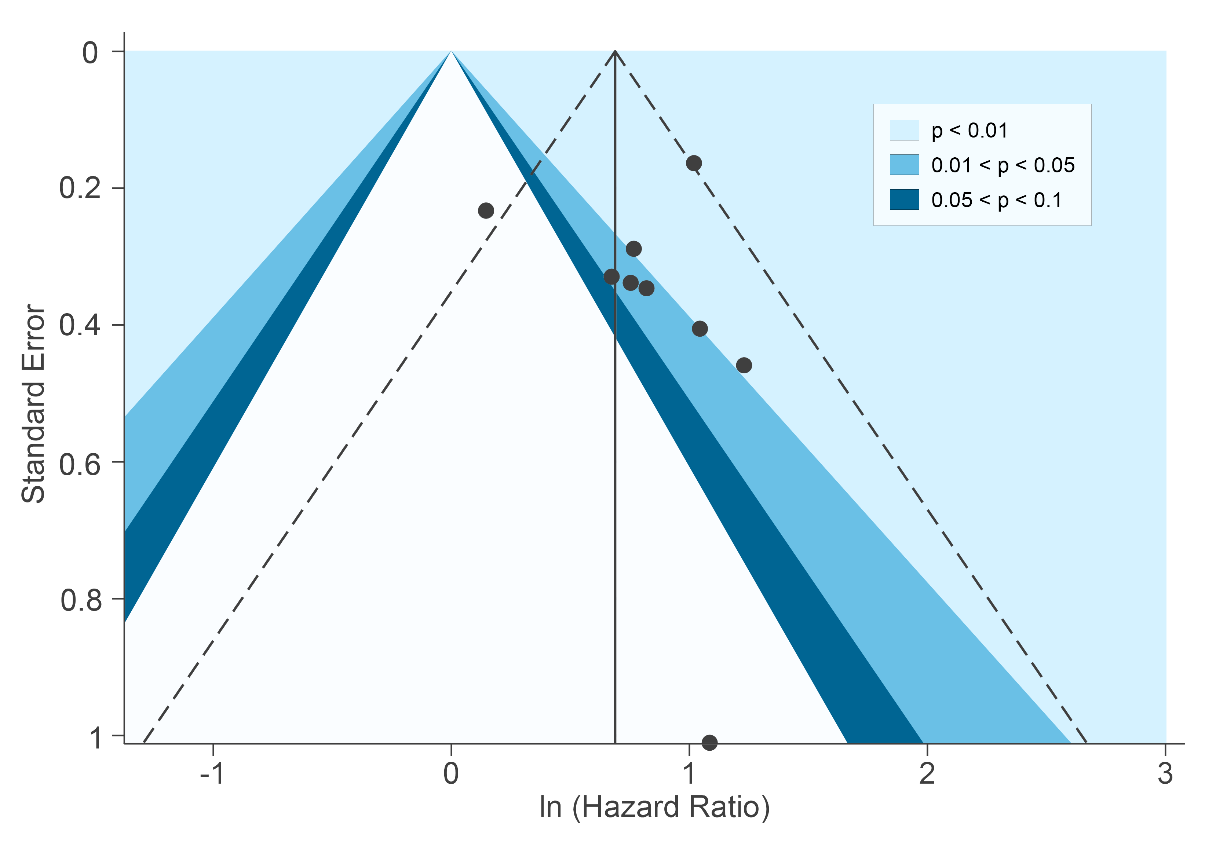
**
